# C=C Dissociative Imination of Styrenes by a Photogenerated Metallonitrene

**DOI:** 10.1021/jacsau.4c00571

**Published:** 2024-09-03

**Authors:** Till Schmidt-Räntsch, Hendrik Verplancke, Annemarie Kehl, Jian Sun, Marina Bennati, Max C. Holthausen, Sven Schneider

**Affiliations:** a Institut für Anorganische Chemie and International Center for Advanced Studies of Energy Conversion, Georg-August-Universität Göttingen, Tammannstraße 4, 37077 Göttingen, Germany; b Institut für Anorganische und Analytische Chemie, Goethe-Universität Frankfurt am Main, Max-von-Laue-Str. 7, 60438 Frankfurt am Main, Germany; c Research Group EPR spectroscopy, Max Planck Institute for Multidisciplinary Sciences, Am Fassberg 11, 37077 Göttingen, Germany; d Institut für Physikalische Chemie, Tammannstraße 6, 37077 Göttingen, Germany

**Keywords:** Aziridination, Nitrene, EPR, Reaction Mechanism, Platinum, Radical Reactivity

## Abstract

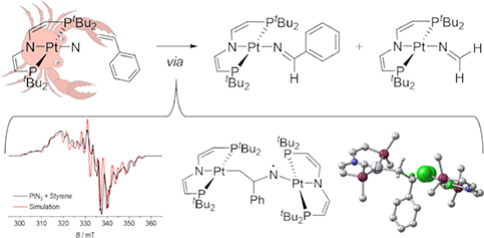

Photolysis of a platinum(II) azide complex in the presence of styrenes enables C=C double bond cleavage upon dissociative olefin imination to aldimido (Pt^II^–N=CHPh) and formimido (Pt^II^–N=CH_2_) complexes as the main products. Spectroscopic and quantum chemical examinations support a mechanism that commences with the decay of the metallonitrene photoproduct (Pt^II^–N) via bimolecular coupling and nitrogen loss as N_2_. The resulting platinum(I) complex initiates a radical chain mechanism via a dinuclear radical-bridged species (Pt^II^–CH_2_CHPhN^•^–Pt^II^) as a direct precursor to C–C scission. The preference for the Pt^I^ mediated route over styrene aziridination is attributed to the distinct nucleophilicity of the triplet metallonitrene.

Nitrene (N–R) transfer methodologies, such as olefin aziridination, have emerged as versatile synthetic strategies for C–N bond formation.^1−3^ Mechanistic scenarios range from direct (photo)generation of free nitrenes or nitrenyl radical anions^4−7^ to catalytic protocols via formal metal imido intermediates with nitrenoid character.^8−13^ C–N bond formation generally relies on olefin addition to these subvalent, electrophilic species.^14,15^ Recently, cobalt catalyzed full C=C bond cleavage of 1,2-diarylalkenes to oxime ethers was also reported,^16^ reflecting more frequently observed dissociative oxygenation of styrenes by high-valent oxo species.^17−20^

In contrast, the exploitation of metal nitrido species for *nitrogen atom transfer* is surprisingly limited.^21^ Carreira’s pioneering work demonstrated the potential of high valent nitrides (Mn^V^) for organic synthesis.^22^ Olefin aziridination with an electrophilic nitrido complex (Ru^VI^) was first reported by Lau.^23^ Smith and co-workers later proposed a two-step radical pathway for styrene addition to an Fe^IV^ nitride (Scheme 1a).^24,25^ Inspired by Brown’s initial report on *N*-atom insertion into C=C double bonds (Scheme 1b),^26,27^ electrophilic nitrides were also utilized by several groups for single N atom editing of cyclic hydrocarbons.^28−30^ While these stoichiometric examples demonstrate intriguing prospects toward aziridination or dissociative C=C imination, more detailed understanding of the reactivity of molecular nitrides is required to facilitate catalytic protocols.

**Scheme 1:**
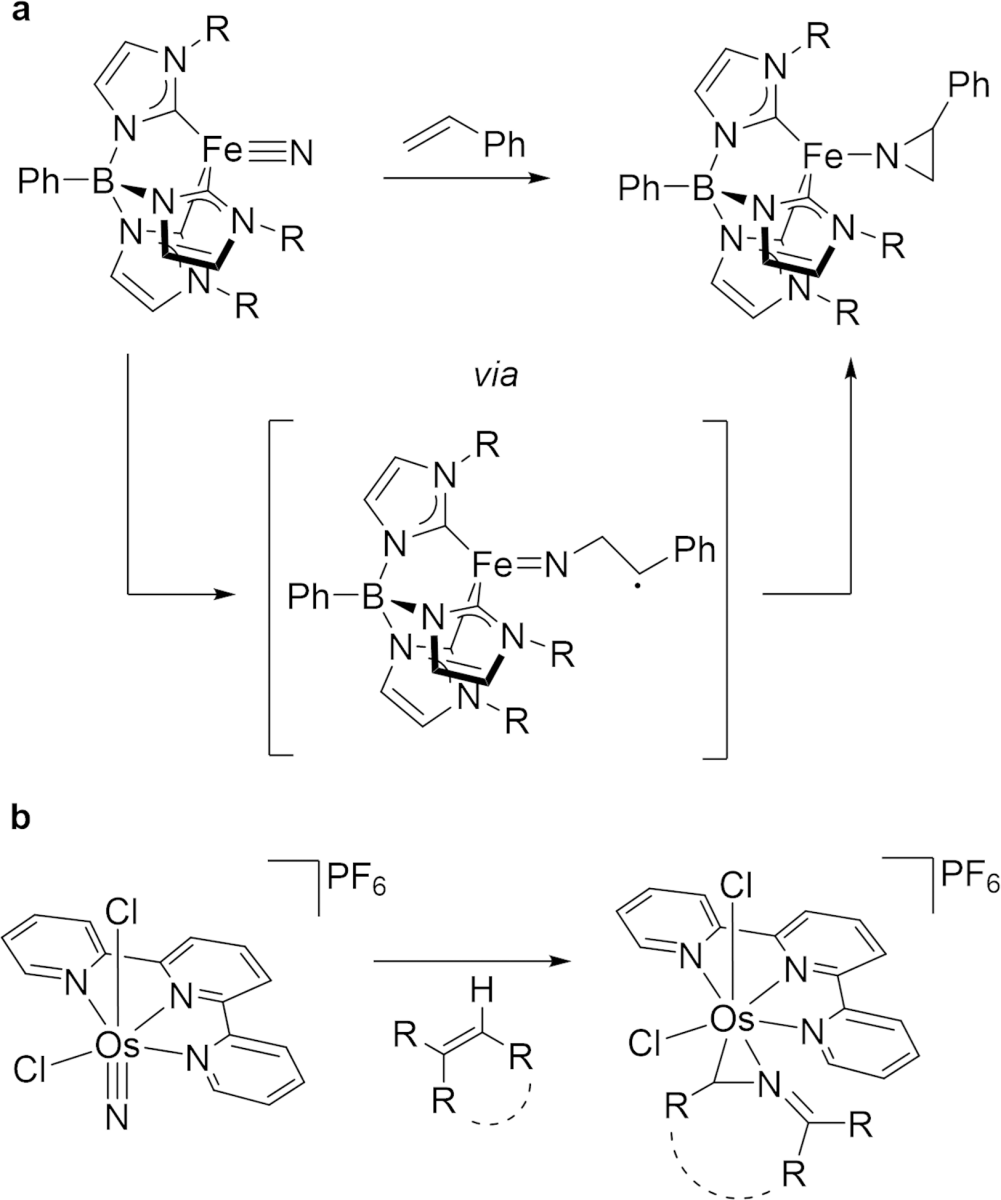
Examples for C–N Bond Formation of Terminal Nitrido Complexes with Olefins^24−27^

Our groups recently reported that the photolysis of the platinum(II) azido complex [Pt(N_3_)(PNP)] (**1**, PNP = N(CHCHP^*t*^Bu_2_)_2_; Scheme 2) gives rise to the formal nitrido complex [Pt(N)(PNP)] (**2**).^31^ Magnetic, crystallographic, and computational data supported the notion of an authentic, metal-substituted nitrene, i.e., a monovalent nitrogen diradical that is bound to a closed-shell platinum(II) fragment. The metallonitrene undergoes a variety of *N*-atom insertion reactions, such as aldehyde amidation.^31,32^ Moving to the Pd analogue enabled the first protocol for catalytic nitrogen atom transfer.^33^ Notably, the C–N bond formation exhibited distinctly nucleophilic character, contrasting with typical subvalent nitrene transfer reagents or high-valent nitrides. The reactivity with styrenes was therefore examined and is presented in this study.

**Scheme 2:**
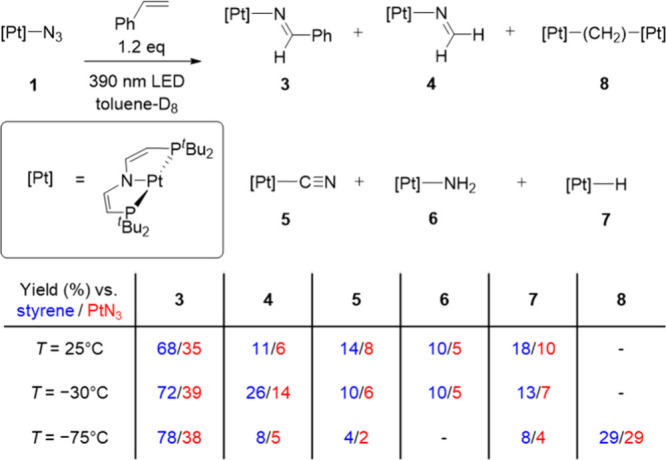
Products from Photolysis of **1** and Styrene at 25, −30, and −75 °C with Respect to Consumed Styrene (Blue) and Pt (Red)

Photolysis of **1** (LED, λ = 390 nm) and styrene (1.2 equiv) in toluene-D_8_ at −30 °C (Scheme 2) gives the aldimido complex [Pt^II^(N=CHPh)(PNP)] (**3**) and formimide [Pt^II^(N=CH_2_)(PNP)] (**4**) in yields of 72% and 26%, respectively, with respect to the olefin. Deuterium labeling confirmed C=C double bond fission as origin of the CHPh and CH_2_ fragments (Figure S72). The significantly lower yield in **4** can be attributed to the formation of cyanido, [Pt^II^(CN)(PNP)] (**5**),^34^ and parent amido, [Pt^II^(NH_2_)(PNP)] (**6**),^31^ complexes as follow-up products. Accordingly, photolysis of a mixture of **1** and **4** gives equal amounts of **5** and **6** and DFT computations confirmed a low effective barrier for the double hydrogen atom transfer (HAT, Δ^‡^*G*_eff_^243^ = 12.0 kcal·mol^–1^; Figure S93). Besides that, minor quantities of [Pt^II^(H)(PNP)] (**7**)^31^ and traces of ethylene (≈1%) were detected. **3** and **4** were also independently prepared and fully characterized.

Excess styrene (1.2 to 100 equiv, Table S2) does not affect the product distribution and is not consumed. The selectivity, however, significantly depends on temperature. At r.t., only slightly lower overall yields are obtained. In contrast, initial photolysis at −75 °C and subsequent warming give all products in similar yields, except **4**. Instead, the methylene bridged diplatinum complex [(CH_2_){Pt^II^(PNP)}_2_] (**8**) is obtained (Scheme 2), which was characterized *in situ* by NMR spectroscopy (see the ESI). Deuterium labeling confirmed styrene parentage of the CH_2_ bridge.

The selectivity determining step(s) were examined by photolysis in the presence of styrene and *para*-substituted derivatives (Figure 1). In all cases, the yields of total aldimides and other products varied only marginally. The aldimido product distributions (log(*k*_X_/*k*_H_)) linearly correlate with Hammett’s substitution parameter σ_p_ with a small, positive reaction parameter (ρ = 1.02 ± 0.06). Notably, this value is close to that reported by Smith for the stepwise radical aziridination of an iron(IV) nitride (ρ = 1.2 ± 0.2; Scheme 1a).^25^ In contrast, electrophilic C=C cleavage by a high-valent Os^VI^ nitride exhibited a negative slope (ρ = −1.5 vs. σ^+^).^29,35^ We note in passing, that the metallonitrene **2** exhibits distinct nucleophilicity (ρ = 4.4) for C–H amidation of aromatic aldehydes.^31^ Finally, reaction with styrene/styrene-D_8_ (1:1) indicated a small, presumably secondary kinetic isotope effect (KIE = 1.16 ± 0.02, Table S4).

**Figure 1:**
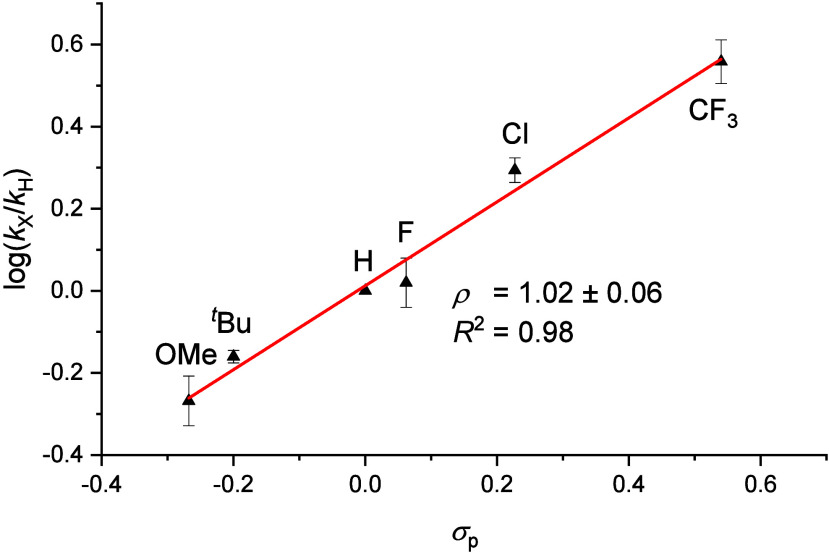
Hammett analysis of the reactivity of **1** with styrene derivatives at −30 °C.

As comparison of the reaction parameter with Smith’s work suggested N-centered radical reactivity, triplet metallonitrene **2** was suspected as key intermediate for styrene activation. In fact, the quantum yield for styrene (Φ = 36 ± 5%) is of similar magnitude as the primary quantum yield for the formation of **2** (Φ = 50 ± 5%) obtained by transient spectroscopy.^36^ Furthermore, the initial photoconversion of azide **1** in frozen solution and subsequent thawing in the presence of styrene gives the same product distribution as solution photolysis at −75 °C. Thus, styrene activation is a thermal process that starts with photoproduct formation. Photoconversion of **1** without substrate in frozen 2-methyltetrahydrofuran (2-MeTHF) at −185 °C afforded the UV/vis signature of the metallonitrene with several distinct bands in the visible region (λ_max_ = 380, 390, 430, 520, and 554 nm; Figure S76). The absence of an X-band EPR signal is in line with the formation of a triplet ground state with sizable zero field splitting, which was shown for **2** by *in situ* SQUID magnetometry (*D* = 85 cm^–1^).^31^ The *S* = 1 ground state is further supported by magnetic characterization in solution at −80 °C with Evans’ method (μ_eff_ = 2.9). Thawing and raising the temperature beyond approximately −70 °C results in decay of the UV/vis signature (Figure S76) and the set of paramagnetically shifted ^1^H NMR signals assignable to **2** (Figure S77).

Surprisingly, DFT computations for the direct reaction of styrene with **2** via C–N radical coupling produced a sizable kinetic barrier (Δ^‡^*G*_eff_^243^ = 17.1 kcal·mol^–1^; Figure S86), which seems too high in light of the low thermal stability of **2**. In search of a different route, styrene activation was examined by *in situ* EPR spectroscopy. For this purpose, photolysis was carried out in frozen 2-MeTHF in the presence of the olefin with subsequent thawing and a rapid freeze quench. This procedure reproducibly afforded a complex signal of an *S* = 1/2 species. Q-band EPR spectroscopy allowed for accurate determination of the *g*-anisotropy (*g* = 2.125, 2.004, and 1.992; Figure S82). Satisfactory simulation of both Q- and X-band (Figure 2) spectra supports dominant hyperfine interactions (HFI) with one {Pt(PNP)} fragment. The ^195^Pt and ^31^P HFI is significantly smaller than for a reported Pt^I^ phosphine complex (*A*_Pt_ = 1900 MHz, *A*_P_ = 700 MHz),^37^ yet close to that of the CN_2_^–^ radical bridged complex [(CN_2_){Pt^II^(PNP)}_2_]^+^.^34^ The notion of a radical-bridged complex is further supported by the large axial ^14^N HFI, which originates from the nitrene group, as shown by ^15^N labeling (Figure S82). Simplification of the spectrum was obtained with CH_2_CDPh as substrate, yet not with the CD_2_CHPh isotopologue, confirming incorporation of the olefin and close proximity of the spin center to the benzylic α-position. The EPR data thus indicate that styrene activation produces an *N*-centered π-radical that originates from the nitrene moiety. Based on the isotopic labeling and quantum-chemical computations that excellently reproduced the EPR data (Figure 2), it can be assigned to the dinuclear, amidyl-bridged complex [(CH_2_CHPhN^•^){Pt^II^(PNP)}_2_] (**9**).

**Figure 2:**
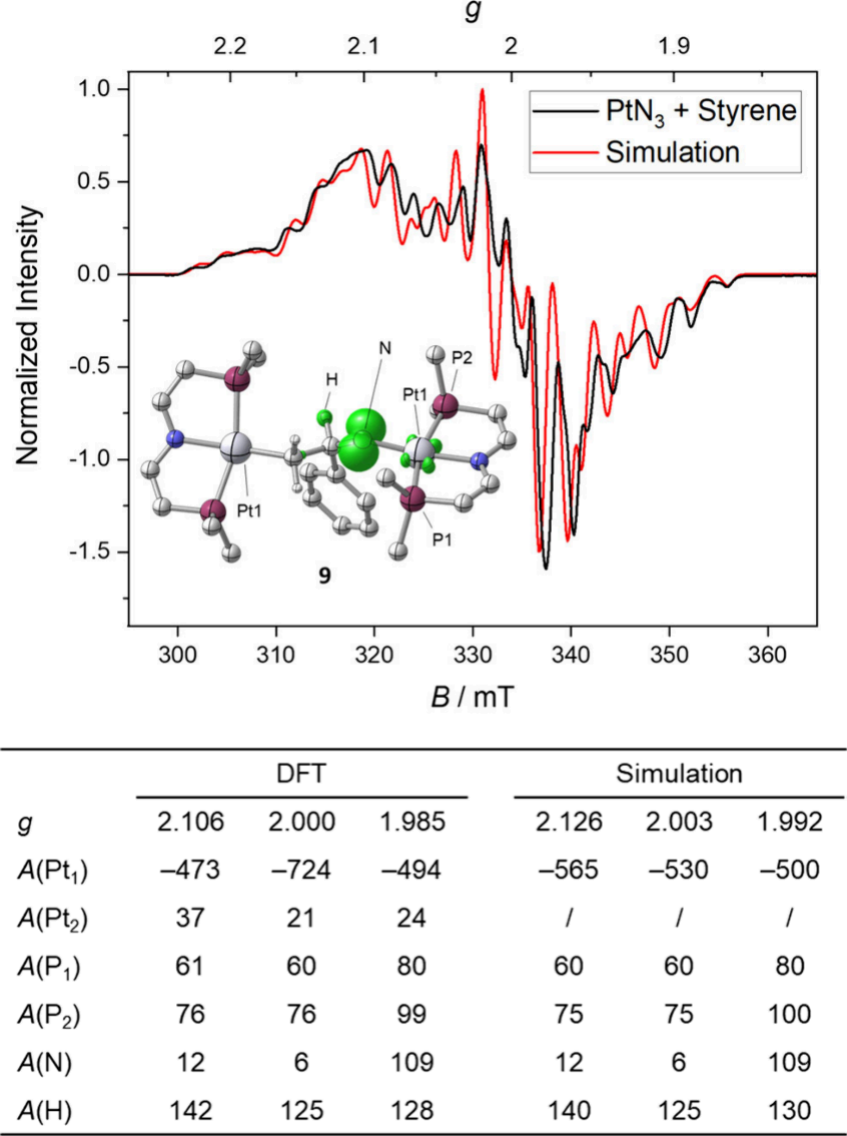
Top: X-band EPR spectrum after photolysis of **1** and styrene in frozen 2-MeTHF, thawing, and rapid freezing to 130 K (inset: computed spin-density plot of **9**, isosurface at 0.01 a_0_^–3^). Bottom: Comparison of the computed (SO-ZORA-PBE0/TZ2P-J) and simulated EPR parameters. Hyperfine couplings *A* are given in MHz.

The generation of **9** implies Pt–C bond formation and thus partial loss of nitrogen. We have previously identified N_2_ formation via N–N coupling as a decay path for a related iridium nitridyl radical complex.^38,39^ In fact, computational evaluation for **2** confirmed strongly exergonic and barrierless dimerization to a diazenido bridged, dinuclear complex, [(N_2_){Pt^II^(PNP)}_2_] (**10**, Figure 3). In contrast to its isolable phosphorus analogue,^40^ N_2_ loss is thermoneutral and rapid as a facile source of platinum(I) as [Pt(PNP)] (**11**). The groups of Ozerov and Chaplin have pioneered the synthesis of two-coordinate Pd(I) and Pt(I) complexes.^41−43^ Gade and co-workers most recently reported a structurally related palladium(I) complex.^44^ Three-coordinate Pt^I^ radicals are generally highly reactive,^37^ providing a potential source for the hydride **7** via HAT. However, the computations also indicated that the coupling of **2** and **11** to the nitridyl bridged dinuclear Pt^II^/Pt^II^ complex **12** is about thermoneutral at −30 °C (Δ*G*^243^ = −2.4 kcal·mol^–1^, Figure S88). A related Rh^I^/Rh^I^ nitridyl complex has been previously reported.^45^ Thus, **12** might serve as a storage state for the fleeting species **2** and **11** upon entropically driven dissociation.

**Figure 3:**
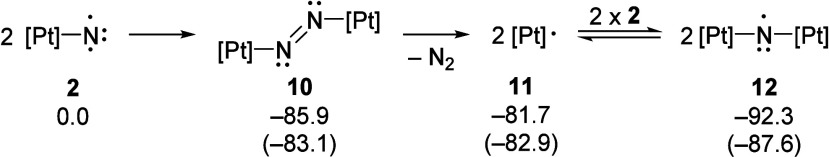
Computed free energies for the decay of **2** via N–N coupling at 203 K (243 K) in kcal**·**mol^–**1**^ ([Pt] = {Pt(PNP)}).

Since the temperature for styrene activation coincides with the decay of **2**, olefin activation by platinum(I) was considered as a potential path. As control reaction, α-methylstyrene was used, which carries an activated methyl group for trapping via HAT (Scheme 3). In that case, neither the analogue of **9**, nor any other C–N coupling product, were observed. Instead, the η^1^-2-phenylallyl complex [(PNP)Pt^II^(CH_2_C(Ph)CH_2_)] (**13**) and **6** are obtained in 2:1 ratio, besides small amounts of hydride **7**. Deuterium labeling (Figure S58) confirmed selective Pt–C bond formation at the vinylic terminus of methylstyrene, disfavoring free allyl radical intermediates. Thus, the control experiment supports styrene activation by platinum(I) complex **11** and subsequent product formation by HAT to nitrene **2**.

**Scheme 3:**
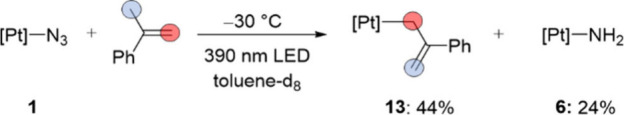
Photolysis of **1** and α-Methylstyrene with Spectroscopic Yields vs Internal Standard [Pt] = {Pt(PNP)}. The colored circles indicate the H/D labelling results. In addition, 4% of hydride **7** was observed.

Based on the spectroscopic and quantum-chemical results, the mechanism shown in Scheme 4 is proposed. Styrene activation by Pt^I^ (**11**) was computed to exhibit a minute activation barrier (5.2 kcal·mol^–1^) and give a Pt^II^ alkyl complex with a remote, benzylic radical, [(PNP)Pt(CH_2_C^•^HPh)]. The redox activity of the olefin reflects results by de Bruin for ethylene activation by Ir^II^.^46,47^ Formation of key intermediate **9** is completed by barrierless and highly exergonic recombination with free nitrene **2**. We thus attribute the moderately positive Hammett slope to the charge flow that is associated with the selectivity-determining addition of olefin to the Pt^I^ radical. The preference for olefin activation by Pt^I^ over that by triplet metallonitrene is attributed to the distinct nucleophilicity of the latter.

**Scheme 4:**
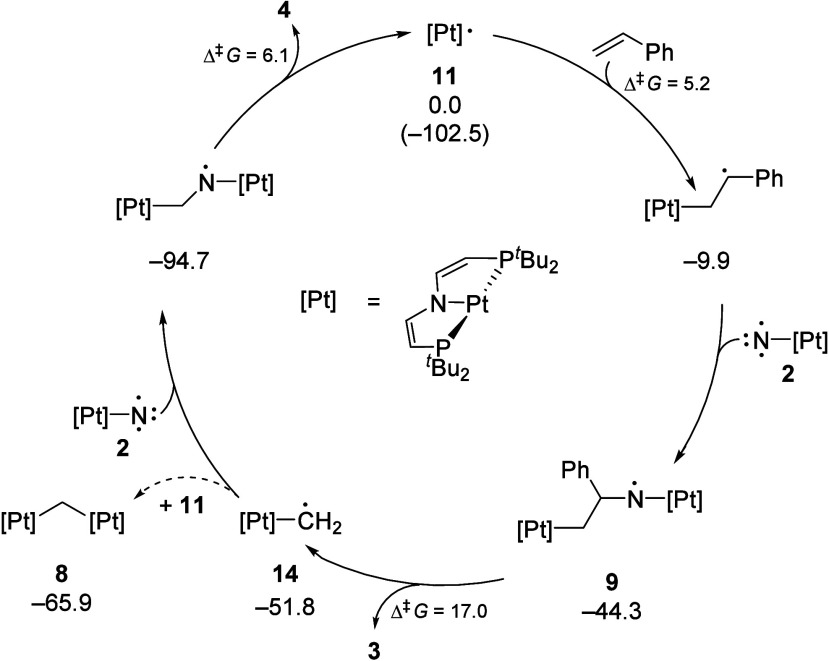
Computed Radical Chain Mechanism for Dissociative Olefin Photoimination Δ*G*^243^ in kcal**·**mol^–**1**^; activation barriers Δ^‡^*G* for individual elementary steps are given relative to the respective preceding minimum.

Subsequent dissociation of **9** into the aldimido product **3** and the methylene complex [(PNP)Pt(CH_2_)] (**14**) exhibits the highest overall kinetic barrier (17.0 kcal·mol^–1^ at *T* = 243 K), which is in line with the spectroscopic detection of **9**. Notably, **14** exhibits predominant Pt^II^ alkyl radical, rather than Pt^III^ alkylidene character. Thus, recombination with triplet nitrene **2** is barrierless and highly exergonic. The dinuclear amidyl radical [(CH_2_N){Pt(PNP)}_2_] is a direct precursor to the formimido product **4** and Pt^I^ species **11**, closing the redox cycle with a minute kinetic barrier (6.1 kcal·mol^–1^).

Formation of **8** instead of **4** after initial photolysis at −75 °C indicates trapping of **14** by Pt^I^ species **11** instead of nitrene **2** (Scheme 2). This path requires high Pt^I^ steady state concentrations, as a consequence of rapid, bimolecular N_2_ loss at high metallonitrene concentrations. At low temperatures, Pt^I^ can accumulate within dinuclear species **12**, which dissociates upon warming.

In summary, photolysis of platinum(II) azide **1** in the presence of styrenes results in dissociative imination of the olefin. Full C=C bond cleavage of styrenes is frequently observed in oxidation catalysis, but mechanistically generally not well understood. Our results indicate that the immediate photoproduct, metallonitrene **2**, does not directly activate the alkene. Instead, Hammett analysis, spectroscopic identification of key intermediates, control experiments, and quantum-chemical evaluation, support a mechanism that relies on a platinum(I) mediated radical chain, which is initiated by nitrene decay via bimolecular N–N coupling. The full C=C scission therefore results from joint reactivity of platinum(I) and the triplet nitrene. Conceptually, they can be considered a “frustrated radical pair”, providing a potential starting point for future approaches to dissociative oxidation and imination of olefins.

In turn, styrene radical activation exclusively by nitrene **2** cannot kinetically compete, explaining the absence of aziridination products. At the current point, we thus associate the selectivity with the distinct nucleophilicity of **2**. The nucleophilic character of the metallonitrene is a defining difference as compared to formal metal imido intermediates in nitrene transfer catalysis or high valent nitrido complexes with nitrenoid character, which exhibit electrophilic reactivity.

## Supporting Information Available

Experimental details and spectroscopic, crystallographic, and computational data (PDF)
Crystallographic data for **3**-CF_3_ (CIF)
Crystallographic data for **3**-OMe (CIF)
Crystallographic data for **4** (CIF)
xyz coordinates (TXT)

## Supplementary Material

au4c00571_si_001.pdf

au4c00571_si_002.cif

au4c00571_si_003.cif

au4c00571_si_004.cif

## Accession Codes

CCDC 2339414 (**4**), CCDC 2339415 (**3**-CF_3_), and CCDC 2339416 (**3**-OMe) contain the supplementary crystallographic data for this paper. These data can be acquired free of charge via http://www.ccdc.cam.ac.uk/data_request/cif, or by emailing data_request@ccdc.cam.ac.uk, or by contacting The Cambridge Crystallographic Data Centre, 12 Union Road, Cambridge CB21EZ, UK; fax: + 44 1223 336033.

## Abbreviations

EPR: electron paramagnetic resonance
DFT: density functional theory
NMR: nuclear magnetic resonance
