# Supplementary material for: C=C Dissociative Imination of Styrenes by a Photogenerated Metallonitrene
Source: JACS Au. 2024 Sep 3;4(9):3421–6. doi: 10.1021/jacsau.4c00571 (PMC11423323; doi:10.1021/jacsau.4c00571)
Supplement: Supplementary file 1 — au4c00571_si_001.pdf [file au4c00571_si_001.pdf]

Supporting Information

C=C Dissociative Imination of Styrenes by a Photogenerated Metallonitrene

Till Schmidt-Räntsch,[a,‡] Hendrik Verplancke,[b,‡] Annemarie Kehl,[c] Jian Sun,[a] Marina Bennati,[c] Max C. Holthausen,[b,*] Sven Schneider[a,*]

[a] Georg-August-Universität Göttingen, Institut für Anorganische Chemie, Tammannstraße 4, 37077 Göttingen, Germany.

[b] Institute of Inorganic and Analytical Chemistry, Johann Wolfgang Goethe-Universität Frankfurt am Main, Max-von-Laue-Str. 7, 60438 Frankfurt am Main, Germany.

[c] Research Group EPR spectroscopy, Max Planck Institute for Multidisciplinary Sciences, Am Fassberg 11, 37077 Göttingen, Germany.

* max.holthausen@chemie.uni-frankfurt.de; sven.schneider@chemie.uni-goettingen.de

Table of Content

1 Experimental ... 4
1.1 Materials and Synthetic Methods ... 4
1.1.1 Analytical Methods ... 4
1.1.2 Photolysis Setup ... 4
1.2 Syntheses and Spectroscopic Characterization ... 5
1.2.1 [Pt{NC(H)(C_6H_5)}{N(CHCHP^{t}Bu$_2$)$_2$}] (**3**) ... 5
1.2.2 [Pt{NC(H)(C_6H_4-*p*-OMe)}{N(CHCHP^{t}Bu$_2$)$_2$}] (**3**-OMe) ... 8
1.2.3 [Pt{NC(H)(C_6H_4-*p*-Cl)}{N(CHCHP^{t}Bu$_2$)$_2$}] (**3**-Cl) ... 11
1.2.4 [Pt{NC(H)(C_6H_4-*p*-CF_3)}{N(CHCHP^{t}Bu$_2$)$_2$}] (**3**-CF_3) ... 14
1.2.5 [Pt{NC(H)(C_6H_4-*p*-F)}{N(CHCHP^{t}Bu$_2$)$_2$}] (**3**-F) ... 17
1.2.6 [Pt{NC(H)(C_6H_4-^{t}Bu)}{N(CHCHP^{t}Bu$_2$)$_2$}] (**3**-^{t}Bu) ... 20
1.2.7 [Pt(NCH_2){N(CHCHP^{t}Bu$_2$)$_2$}] (**4**) ... 23
1.2.8 1-Phenyl-1-CD_3-ethene[4,5] ... 26
1.3 Photolysis of **1** and styrene at –30 °C ... 29
1.4 Photolysis of **1** and styrene at –75 °C ... 30
1.5 Photolysis of **1** and styrene in frozen solution ... 36
1.6 Photolysis of **1** and *α*-methylstyrene ... 37
1.6.1 Photolysis of **1** and D_3-*α*-methylstyrene ... 42
2 Mechanistic Examinations ... 44
2.1 Light source ... 44
2.2 Quantum Yield ... 46
2.3 Hammett Analysis ... 47
2.4 Variation of Styrene Concentration ... 53
2.5 Irradiation at room temperature (25 °C) ... 53
2.6 Selective deuteration of styrene ... 53
2.7 Kinetic Isotope Effect ... 55
2.8 Photolysis of **1** at low temperatures ... 57
2.8.1 UV/Vis spectroscopy ... 57
2.8.2 NMR spectroscopy ... 57
2.9 Photolysis of **1** in presence of **4** ... 59
2.10 Photolysis of **1** in the absence of substrate ... 60
3 EPR Spectroscopy ... 62
4 Crystallographic Details ... 64
4.1 Crystal Structure of [(PNP)Pt(NCH(C_6H_4OMe)] (**3**-OMe) ... 65
4.2 Crystal Structure of [(PNP)Pt(NCH(C_6H_4OMe)] (**3**-CF_3) ... 69

4.3 Crystal Structure of [(PNP)Pt(NCH_2)] (**4**) 73
5 Computational Details 77
Additional Figures 77
Cartesian Coordinates 82
References 82

1 Experimental

1.1 Materials and Synthetic Methods

All experiments were carried out under inert conditions (Argon, Linde 5.0) using standard Schlenk and glove-box techniques (argon atmosphere). All solvents were purchased in HPLC quality (Sigma Aldrich) and dried using an MBRAUN Solvent Purification System. Deuterated solvents were obtained from Eurisotop GmbH, dried over Na/K alloy (THF-D_8, Tol-D_8, C_6D_6) and distilled by trap-to-trap transfer *in vacuo*. [Pt(OTf)(PNP)], [Pt(N_3)(PNP)] (**1**), [Pt(CN)(PNP)] (**5**), [Pt(NH_2)(PNP)] (**6**) and [PtH(PNP)] (**7**) have been reported and characterized previously [1,2] All other chemicals were purchased from chemical vendors and used as received unless otherwise stated.

1.1.1 Analytical Methods

NMR spectra were recorded in J-Young tubes on Bruker Avance III HD 300, Avance III HD 400, Avance Neo 400, Avance III HD 500, or Avance Neo 600 NMR spectrometers at a temperature of 298 K, unless stated otherwise. ^{1}H NMR spectra were referenced to the residual solvent signal (C_6D_6: δ_H = 7.16 ppm; THF-D_8: δ_H = 3.58 ppm; Tol-D_8: δ_H = 2.09 ppm) as internal standard. Chemical shifts for ^{13}C, ^{15}N, ^{31}P, and ^{195}Pt are given *vs*. Me_4Si, H_3PO_4, $MeNO_2$, and Na_2PtCl_6, respectively. All heteronuclei were referenced *via* the proton spectrum by using the Ξ value, as recommended by IUPAC.[3] Signal multiplicities are abbreviated as s (singlet), d (doublet), t (triplet), q (quartet), quint (quintet), m (multiplet), dd (doublet of doublets), dt (doublet of triplets), td (triplet of doublets), vt (virtual triplet), or br (broad). Brackets were used for ^{195}Pt satellites (33.8 % abundance). MestReNova 14.3 (Mestrelab Research S.L., Santiago de Compostela, ESP) was used for analysis of NMR spectra. **Elemental analyses** were obtained from the Analytisches Labor, Georg-August-Universität, Göttingen, using an Elementar Vario EL 3 analyzer. **HR-ESI-MS** (Bruker maXis QTOF) and **LIFDI-MS** (JEOL AccuTOF JMS-T100GCV; inert conditions) were measured by the Zentrale Massenabteilung, Fakultät für Chemie, Georg-August-Universität, Göttingen. **IR spectra** were recorded using a Bruker ALPHA FT-IR spectrometer with Platinum ATR module. **UV/vis spectra** were recorded on an Agilent Cary 8454 spectrometer with VT unit (+100 to –195 °C; USP-203 Series, Unisoku, Osaka, JPN) using quartz cuvettes with airtight caps, or for measurements below –180 °C reinforced quartz cuvettes (Hellma, Müllheim, GER) capped with a rubber septum. All UV/vis samples were prepared in a glovebox and transferred to the spectrometer outside the box prior to measurement.

1.1.2 Photolysis Setup

Photolysis experiments were performed using Kessil (DiCon Fiberoptics & DiCon Lighting, Richmond, CA, USA) PR160L LEDs with 370 nm or 390 nm wavelengths or a 150 W Hg(Xe) arc lamp with a lamp housing and arc lamp power supply from Quantum Design GmbH (Pfungstadt, Hessen, GER). In the case of the Hg(Xe) arc lamp, a white-glass filter with a cutoff wavelength of 305 nm was used. Irradiation inside the NMR spectrometer was carried out by using a commercial setup from Mountain Photonics (Landsberg am Lech, GER), consisting of a 380 nm LED and a fiber optic, both from Prizmatix (Holon, IL). The fiber optic was sanded at the lower 40 mm, allowing irradiation of the sample around the fiber optic. Instead of a J-Young NMR tube, a combination of inner tube (3 mm), surrounding the fiber optic, and outer tube (5 mm), containing the sample solution, was used. The inner tube is inserted into the outer tube through a screw-cap septum, which is further air-tightened by parafilm.

1.2 Syntheses and Spectroscopic Characterization

1.2.1 [Pt{NC(H)(C_6H_5)}{N(CHCHP^{t}Bu$_2$)$_2$}] (**3**)

[Pt(OTf){N(CHCHP^{t}Bu$_2$)$_2$}] (26.7 mg, 0.038 mmol, 1.0 eq.) and PhCN (5.6 µL, 0.054 mmol, 1.4 eq.) are dissolved in THF (2 mL). The mixture is stirred at 25 °C for 16 hours, leading to formation of a yellow suspension. After removal of the solvent, the residue is suspended in THF (1 mL) and a solution of $KBHEt_3$ in THF (1 M, 41 µL, 0.041 mmol, 1.1 eq.) is added at –36 °C. The mixture is warmed to 25 °C and stirred for additional 20 minutes. Removal of the solvent and extraction with Et_2O gives a pale yellow residue after solvent evaporation. Extraction with pentane and recrystallization from pentane at –36 °C yields the product as crystalline solid (11.4 mg, 46%).

^{1}H{^{31}P} NMR: (C_6D_6, 500 MHz, [ppm]): δ = 10.49 (A(X), s+d, $^3J_{HPt}$ = 88.5 Hz, 1H, NC(*H*)Ph), 7.91 (AB, d, $^3J_{HH}$ = 7.8 Hz, 2H, *o*-H), 7.32 (ABC, t, $^3J_{HH}$ = 7.8 Hz, 2H, *m*-H), 7.14 (AB$_2$, t, $^3J_{HH}$ = 7.8 Hz, 1H, *p*-H), 6.98 (AB(X), d+dd, $^3J_{HH}$ = 5.4 Hz, $^3J_{HPt}$ = 49.4 Hz, 2H, NC*H*), 4.07 (AB(X), d+dd, $^3J_{HH}$ = 5.4 Hz, $^3J_{HPt}$ = 35.6 Hz, 2H, PC*H*), 1.31 (s, 36H, ^{t}Bu).

^{13}C{^{1}H} NMR (C_6D_6, 126 MHz, [ppm]): δ = 161.9 (vt+dvt, $^2J_{CPt}$ = 63.7 Hz, $^2J_{CP}$ = 7.6 Hz, N*C*H), 160.6 (vt, $^3J_{CP}$ = 2.1 Hz, N*C*(H)Ph), 142.9 (br s, *i*-C), 128.4 (s, *m*-C), 127.3 (s, *p*-C), 126.1 (s, *o*-C), 82.1 (vt, $^1J_{CP}$ = 23.1 Hz, P*C*H), 36.1 (vt, $^1J_{CP}$ = 13.1 Hz, *C*(CH_3)$_3$), 28.9 (vt, $^2J_{CP}$ = 2.9 Hz, C(*C*H$_3$)$_3$).

^{31}P{^{1}H] NMR (C_6D_6, 203 MHz, [ppm]): δ = 60.5 (s+d, $^1J_{PPt}$ = 2940 Hz).

^{195}Pt{^{1}H] NMR (C_6D_6, 108 MHz, [ppm]): δ = –3723 (t, $^1J_{PtP}$ = 2940 Hz).

ESI-HR-MS m/z found (calc) [$C_{27}H_{46}N_2P_2Pt+H$]$^+$: 656.2870 (656.2862).

Elem. Anal. found (calc) for $C_{27}H_{46}N_2P_2Pt$: C 49.09 (49.46); H 7.49 (7.07); N 4.16 (4.27).

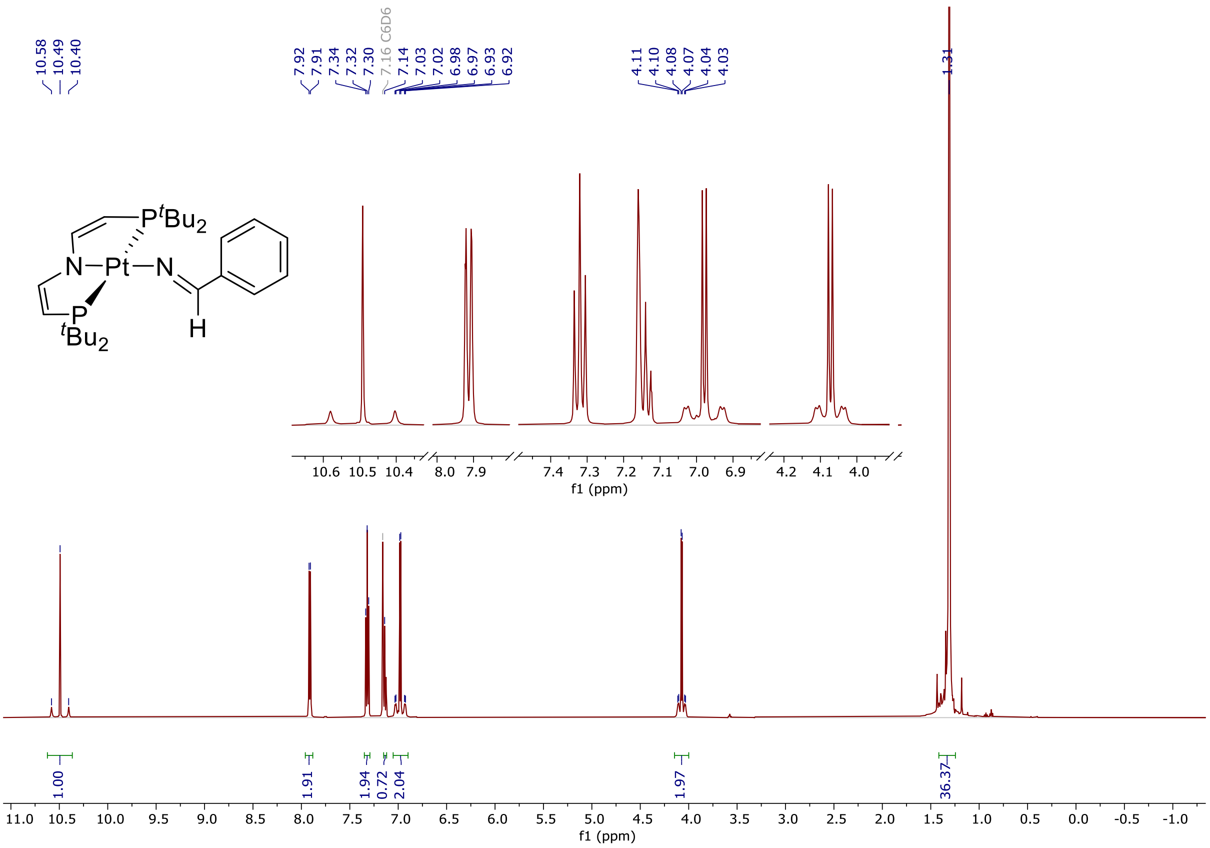

Figure S1: ^{1}H{^{31}P}-NMR spectrum of **3** in C_6D_6.

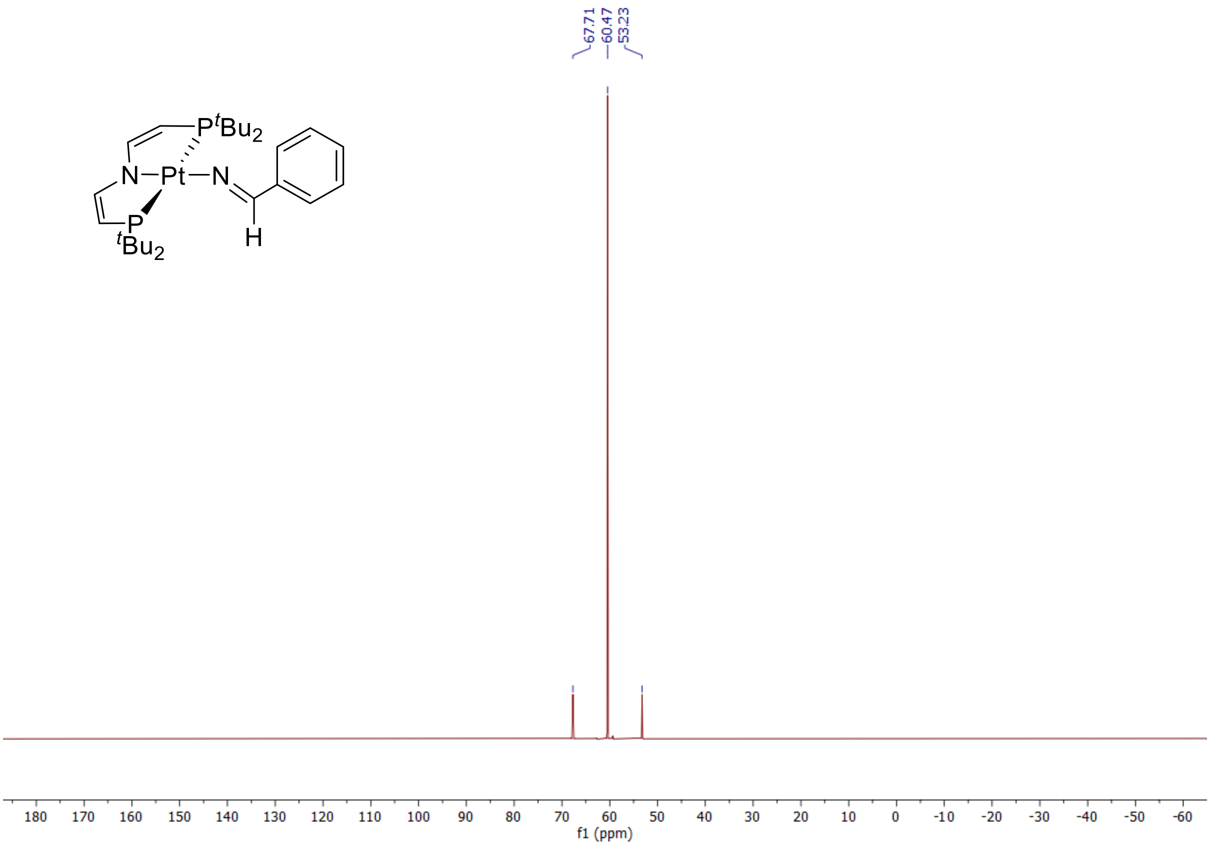

Figure S2: $^{31}P\{^1H\}$-NMR spectrum of **3** in C_6D_6.

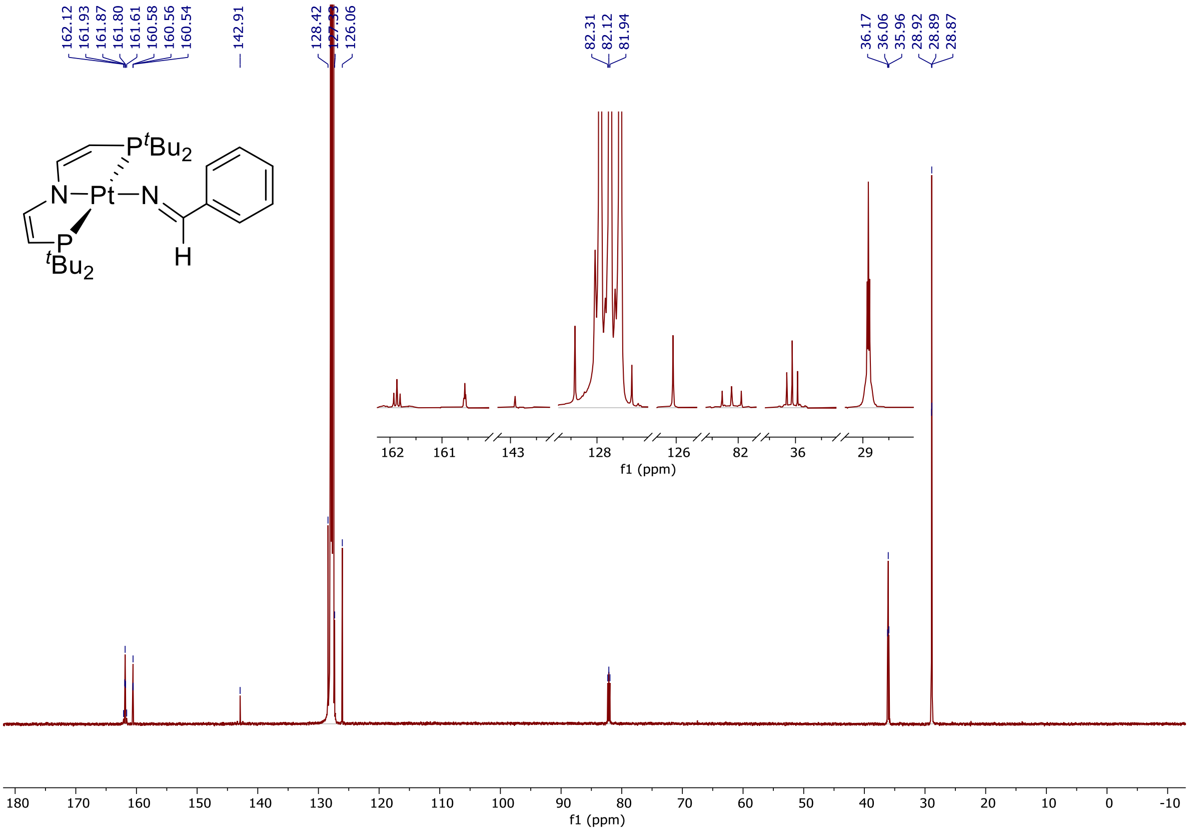

Figure S3: $^{13}C\{^1H\}$-NMR spectrum of **3** in C_6D_6.

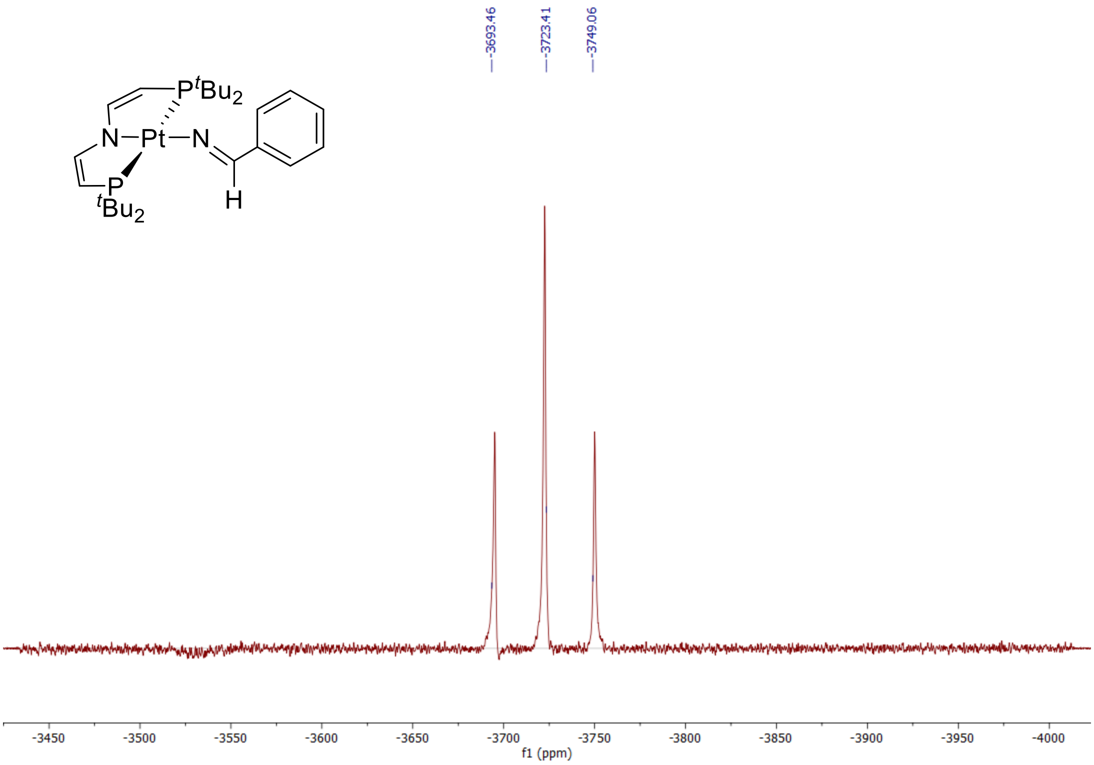

Figure S4: ^{195}Pt{^{1}H}-NMR spectrum of **3** in C_6D_6.

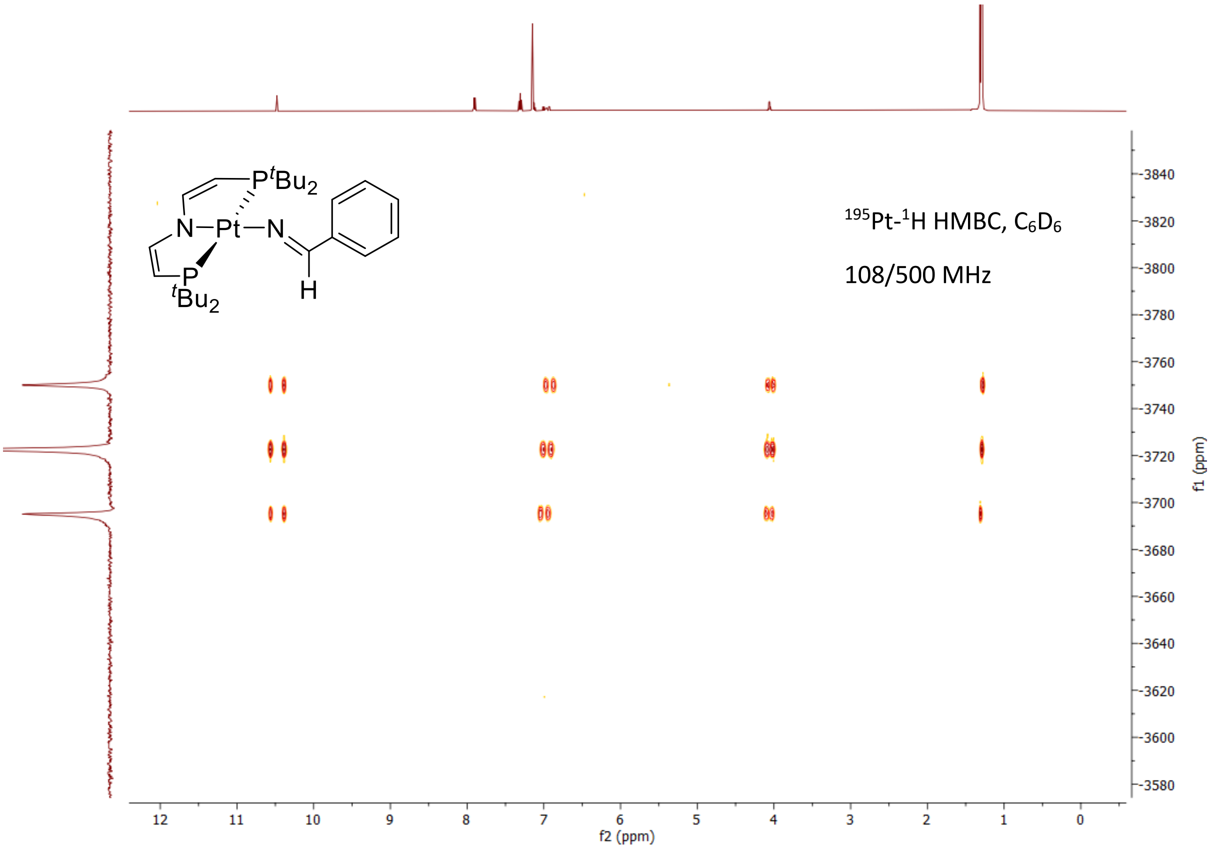

Figure S5: ^{195}Pt-^{1}H-HMBC-spectrum of **3** in C_6D_6.

1.2.2 [Pt{NC(H)(C_6H_4-*p*-OMe)}{N(CHCHP^{t}Bu$_2$)$_2$}] (**3**-OMe)

[Pt(OTf){N(CHCHP^{t}Bu$_2$)$_2$}] (23.0 mg, 0.033 mmol, 1.0 eq.) and 4-methoxybenzonitrile (4.9 mg, 0.037 mmol, 1.1 eq.) are dissolved in THF (2 mL). The mixture is stirred at 25 °C for 24 hours, followed by addition of a solution of $KBEt_3H$ in THF (1 M, 41 µL, 0.041 mmol, 1.1 eq.) at –36 °C. The mixture is warmed to 25 °C and stirred for another 4 hours. Removal of the solvent and extraction with Et_2O and pentane gives a pale yellow solid after solvent evaporation. The product is washed with cold pentane and diethylether and dried *in vacuo*. (5.6 mg, 52%).

^{1}H{^{31}P} NMR: (C_6D_6, 500 MHz, [ppm]): δ = 10.42 (A(X), s+d, $^3J_{HPt}$ = 88.9 Hz, 1H, NC(*H*)Ph), 7.85 (AB, d, $^3J_{HH}$ = 8.8 Hz, 2H, *o*-H), 7.00 (AB(X), d+dm, $^3J_{HH}$ = 5.4 Hz, 2H, NC*H*), 6.92 (AB, d, $^2J_{HH}$ = 8.8 Hz, 2H, *m*-H), 4.09 (AB(X), d+dd, $^3J_{HH}$ = 5.4 Hz, $^3J_{HPt}$ = 35 Hz, 2H, PC*H*), 3.29 (s, 3H, OC*H*$_3$), 1.34 (s, 36H, ^{t}Bu).

^{13}C{^{1}H} NMR (C_6D_6, 126 MHz, [ppm]): δ = 162.2 (vt+dvt, $^2J_{CPt}$ = 62 Hz, $^2J_{CP}$ = 7.6 Hz, N*C*H), 160.0 (vt, $^3J_{CP}$ = 1.9 Hz, N*C*(H)Ph), 159.9 (s, *p*-C), 137.1 (s, *i*-C), 127.4 (s, *o*-C), 114.3 (s, *m*-C), 82.5 (vt, $^1J_{CP}$ = 23.1 Hz, P*C*H), 54.8 (s, O*C*H$_3$), 36.4 (vt, $^1J_{CP}$ = 13.0 Hz, *C*(CH$_3$)$_3$), 29.2 (vt, $^2J_{CP}$ = 2.9 Hz, C(*C*H$_3$)$_3$).

^{31}P{^{1}H] NMR (C_6D_6, 203 MHz, [ppm]): δ = 60.2 (s+d, $^1J_{PPt}$ = 2940 Hz).

^{195}Pt{^{1}H] NMR (C_6D_6, 108 MHz, [ppm]): δ = −3715 (t, $^1J_{PtP}$ = 2940 Hz).

LIFDI-MS m/z found (calc) [$C_{27}H_{46}N_2P_2Pt$]$^+$: 685.2 (685.3).

Elem. Anal. found (calc) for $C_{28}H_{48}N_2OP_2Pt$: C 48.86 (49.04); H 7.04 (7.06); N 3.99 (4.06).

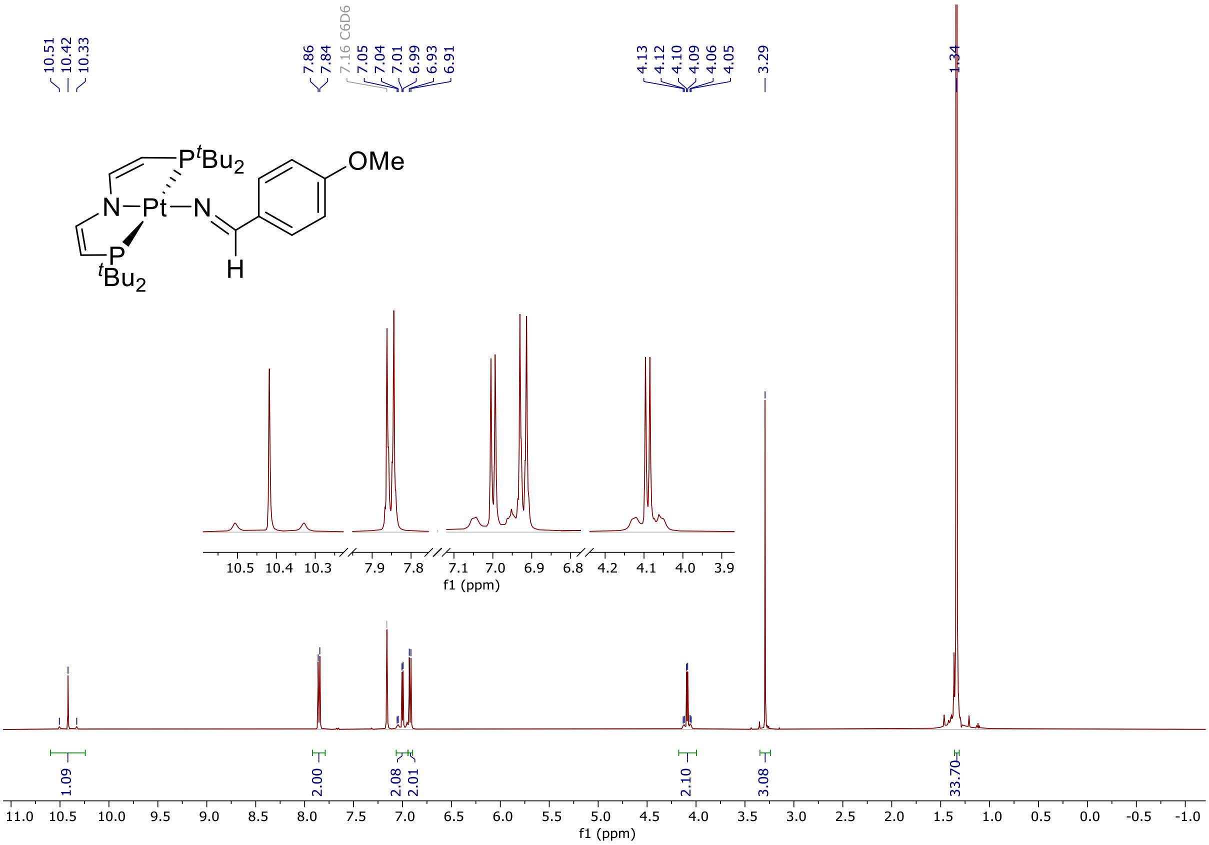

Figure S6: ^{1}H{^{31}P}-NMR spectrum of **3**-OMe in C_6D_6.

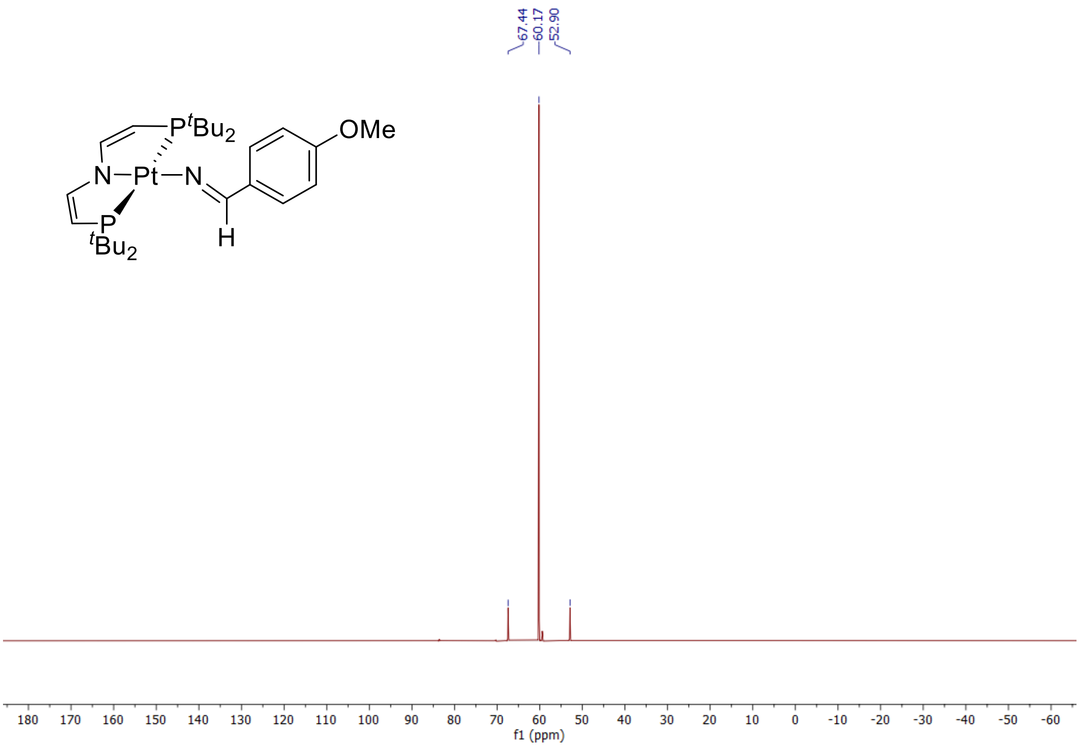

Figure S7: $^{31}P\{^1H\}$-NMR spectrum of **3**-OMe in C_6D_6.

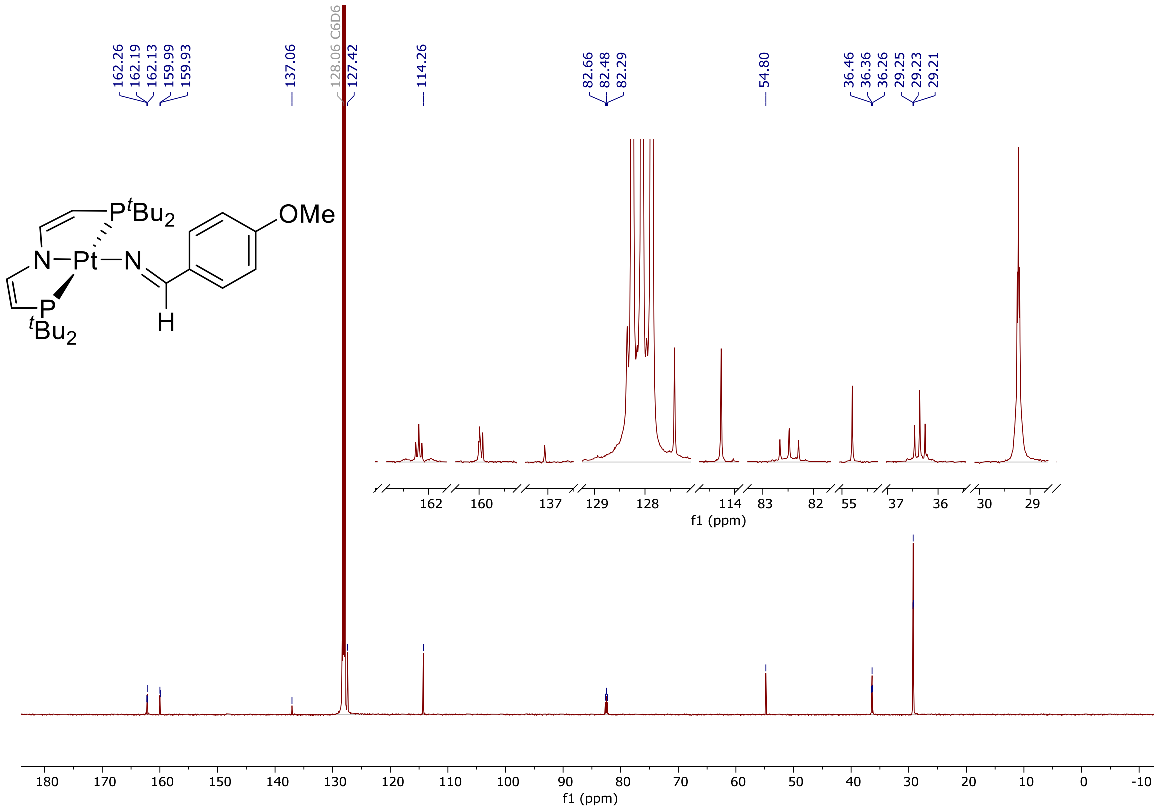

Figure S8: $^{13}C\{^1H\}$-NMR spectrum of **3**-OMe in C_6D_6.

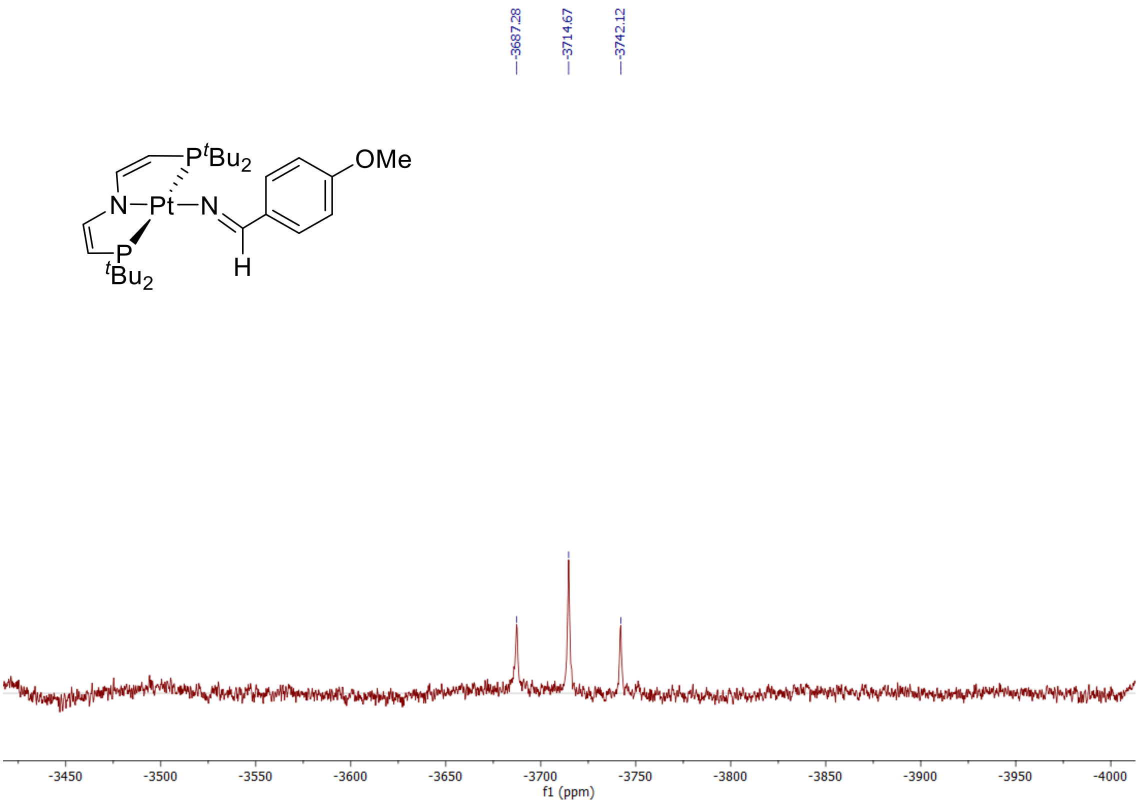

Figure S9: ^{195}Pt{^{1}H}-NMR spectrum of **3**-OMe in C_6D_6.

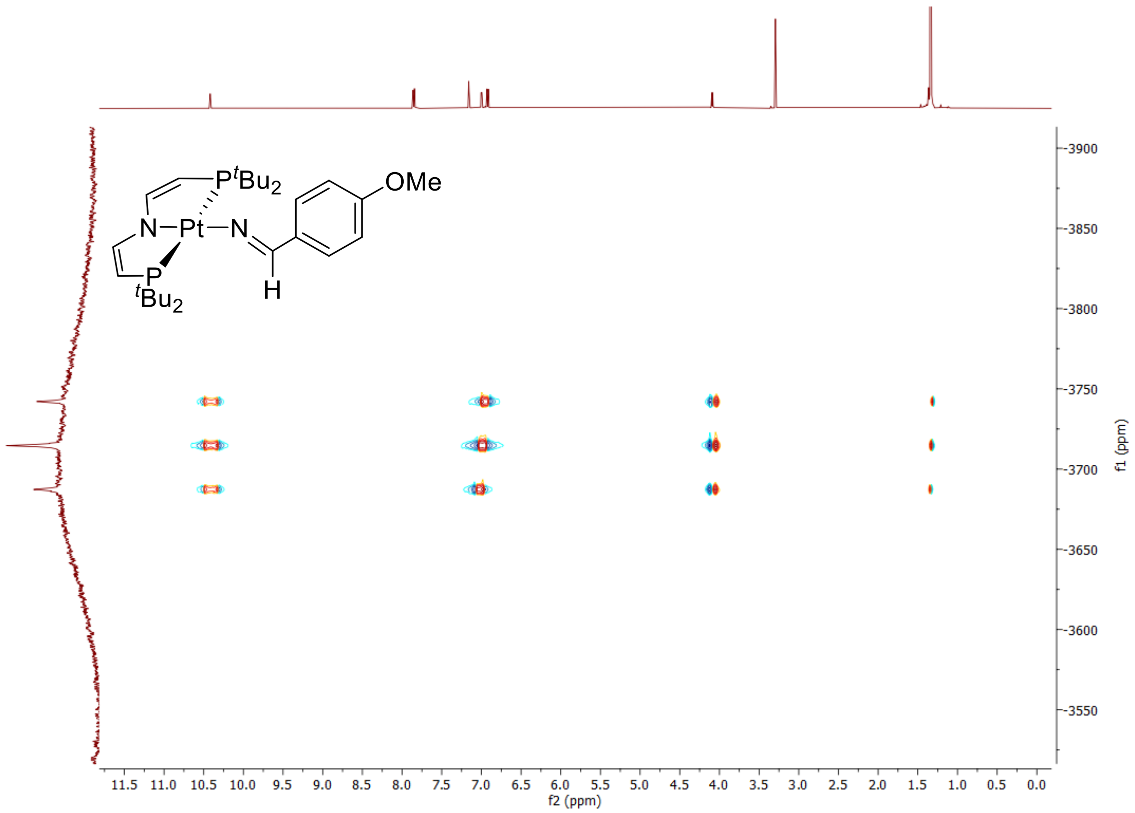

Figure S10: ^{195}Pt-^{1}H-HMBC spectrum (108/500 MHz) of **3**-OMe in C_6D_6.

1.2.3 [Pt{NC(H)(C_6H_4-*p*-Cl)}{N(CHCHP^{t}Bu$_2$)$_2$}] (**3**-Cl)

[Pt(OTf){N(CHCHP^{t}Bu$_2$)$_2$}] (20.5 mg, 0.029 mmol, 1.0 eq.) and 4-chlorobenzonitrile (4.6 mg, 0.033 mmol, 1.1 eq.) are dissolved in THF (1.5 mL). The mixture is stirred at 25 °C for 24 hours, followed by addition of a solution of $KBEt_3H$ in THF (1 M, 30 µL, 0.030 mmol, 1.0 eq.) at –36 °C. The mixture is warmed to 25 °C and stirred for another 2 hours. Removal of the solvent and extraction with pentane (2 mL) gives a yellow solid after solvent evaporation. Crystallization from pentane at – 36°C gives a crystalline, yellow solid (13.8 mg, 68%).

^{1}H{^{31}P} NMR: (C_6D_6, 500 MHz, [ppm]): δ = 10.33 (A(X), s+d, $^3J_{HPt}$ = 88.2 Hz, 1H, NC(*H*)Ph), 7.63 (AB, d, $^3J_{HH}$ = 8.5 Hz, 2H, *o*-H), 7.26 (AB, d, $^2J_{HH}$ = 8.5 Hz, 2H, *m*-H), 6.96 (AB(X), d+dd, $^3J_{HH}$ = 5.4 Hz, $^3J_{HPt}$ = 49 Hz, 2H, NC*H*), , 4.06 (AB(X), d+dd, $^3J_{HH}$ = 5.6 Hz, $^3J_{HPt}$ = 35 Hz, 2H, PC*H*), 1.29 (s, 36H, ^{t}Bu).

^{13}C{^{1}H} NMR (C_6D_6, 126 MHz, [ppm]): δ = 162.2 (vt+dvt, $^2J_{CPt}$ = 65 Hz, $^2J_{CP}$ = 7.6 Hz, N*C*H), 159.3 (vt, $^3J_{CP}$ = 2.1 Hz, N*C*(H)Ph), 141.4 (s+d, *i*-C, $^3J_{CPt}$ = 116 Hz), 133.4 (s, *p*-C), 129.0 (s, *m*-C), 127.4 (s, *o*-C), 82.4 (vt, $^1J_{CP}$ = 23.3 Hz, P*C*H), 36.4 (vt, $^1J_{CP}$ = 13.1 Hz, *C*(CH_3)$_3$), 29.2 (vt, $^2J_{CP}$ = 2.9 Hz, C(*C*H$_3$)$_3$).

^{31}P{^{1}H] NMR (C_6D_6, 203 MHz, [ppm]): δ = 60.8 (s+d, $^1J_{PPt}$ = 2920 Hz).

^{195}Pt{^{1}H] NMR (C_6D_6, 108 MHz, [ppm]): δ = −3707 (t, $^1J_{PtP}$ = 2920 Hz).

LIFDI-MS m/z found (calc) [$C_{27}H_{45}ClN_2P_2Pt$]$^+$: 689.2 (689.2).

Elem. Anal. found (calc) for $C_{27}H_{45}ClN_2P_2Pt$: C 47.11 (46.99); H 6.26 (6.57); N 3.94 (4.06).

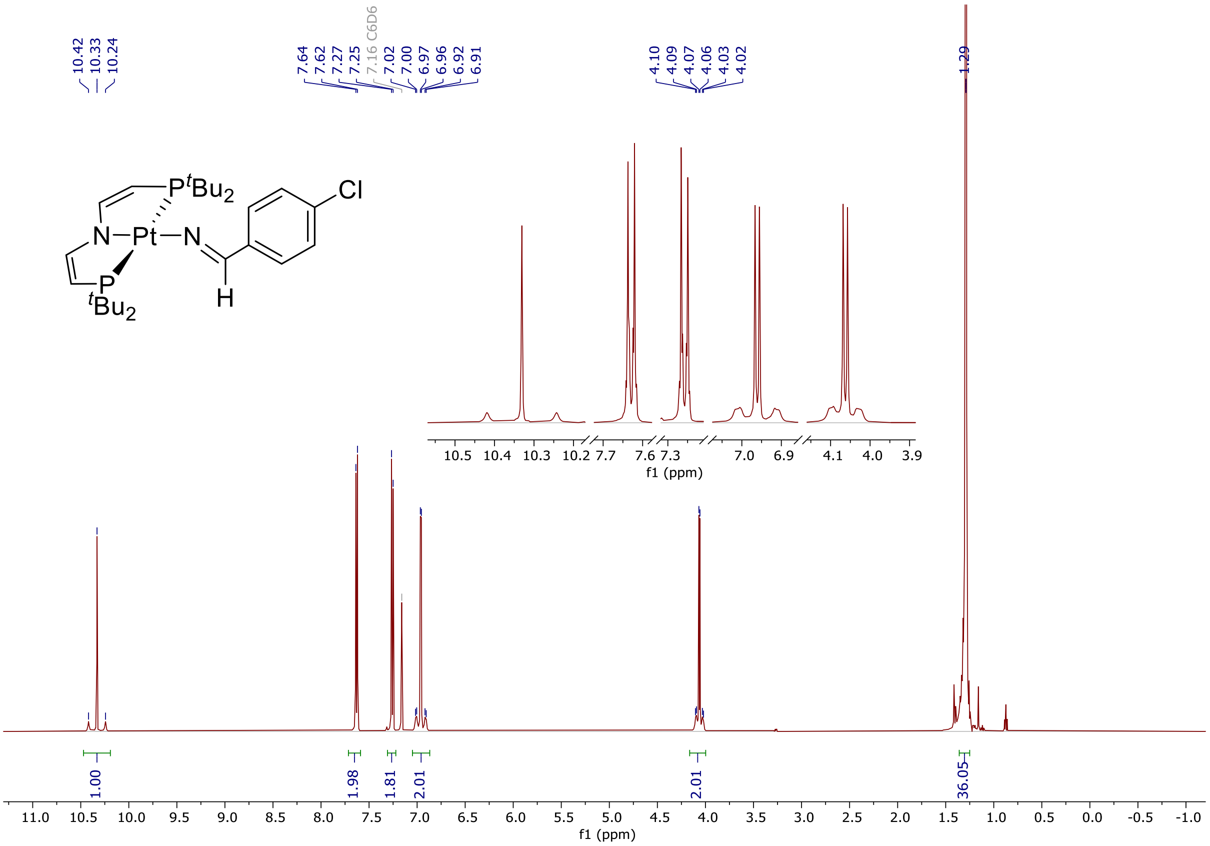

Figure S11: ^{1}H{^{31}P}-NMR spectrum of **3**-Cl in C_6D_6.

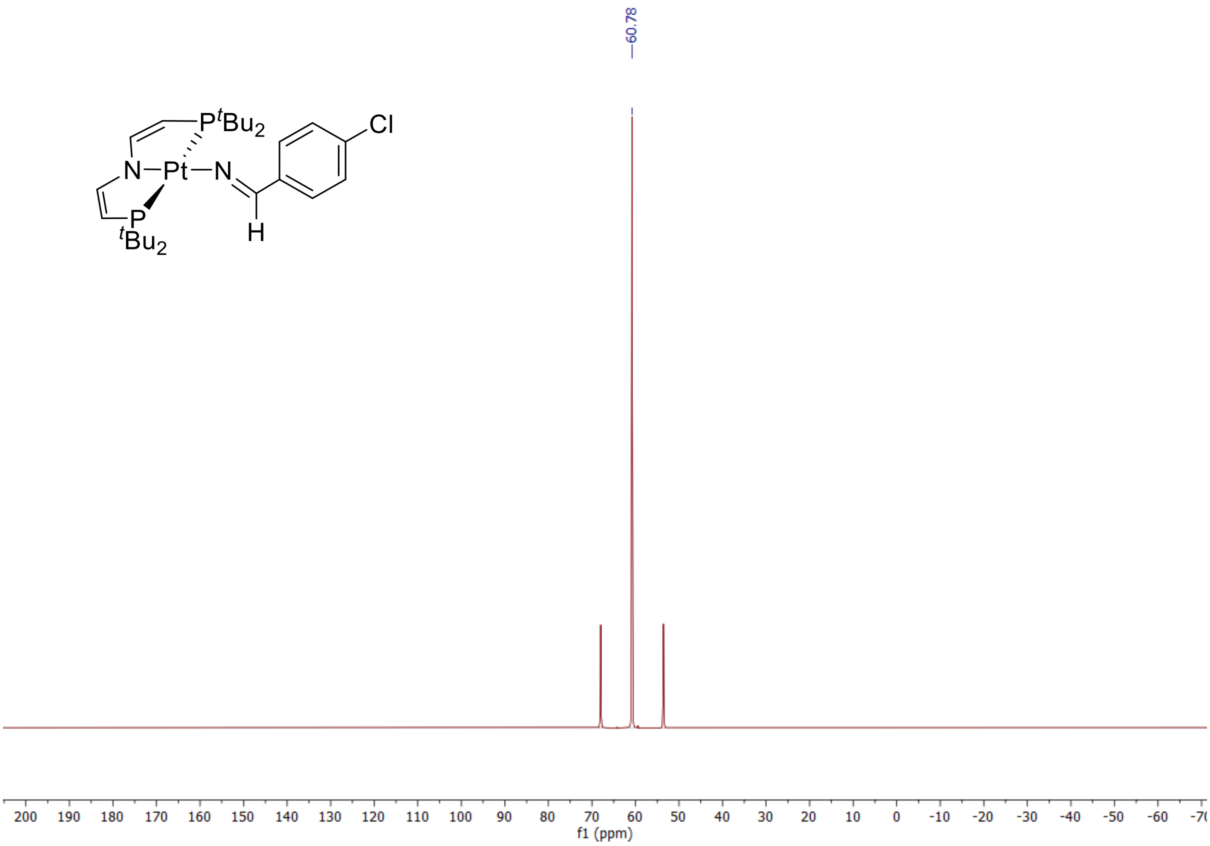

Figure S12: $^{31}P\{^1H\}$-NMR spectrum of **3**-Cl in C_6D_6.

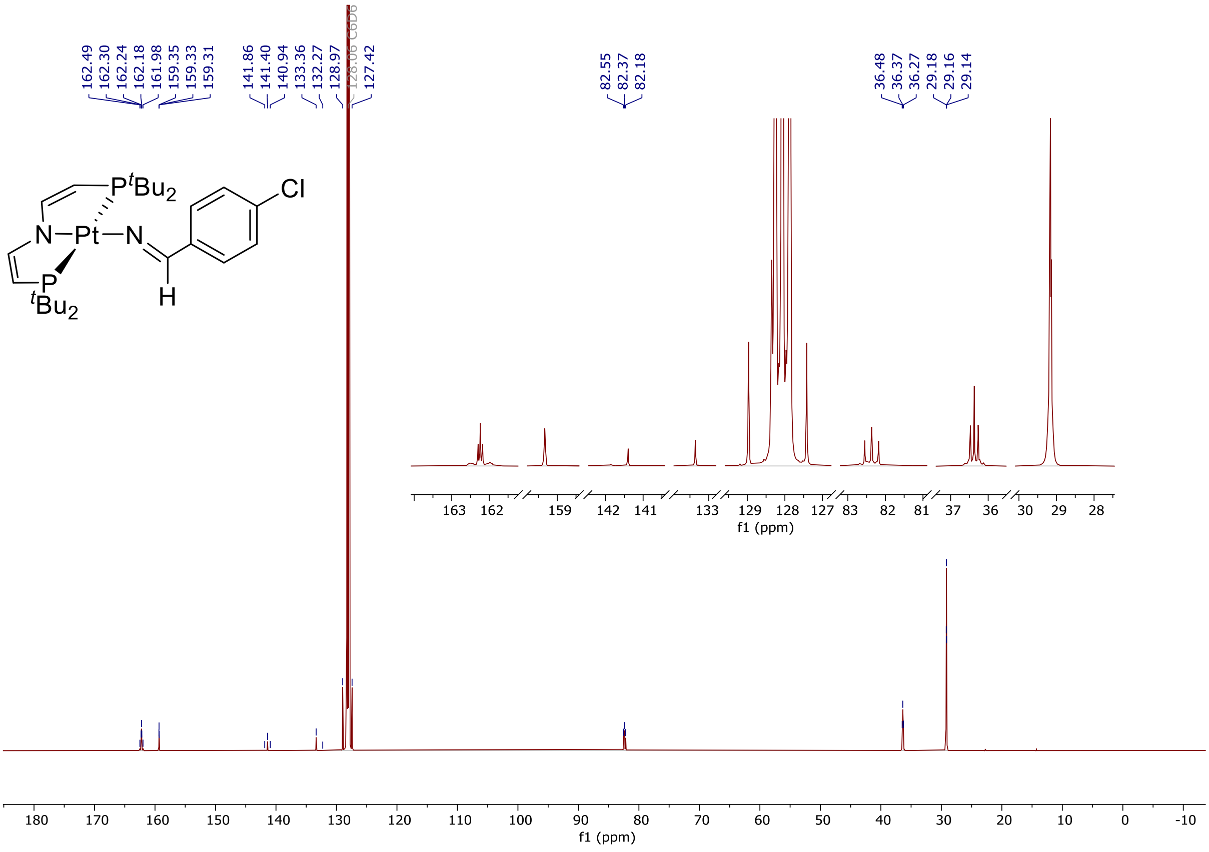

Figure S13: $^{13}C\{^1H\}$-NMR spectrum of **3**-Cl in C_6D_6.

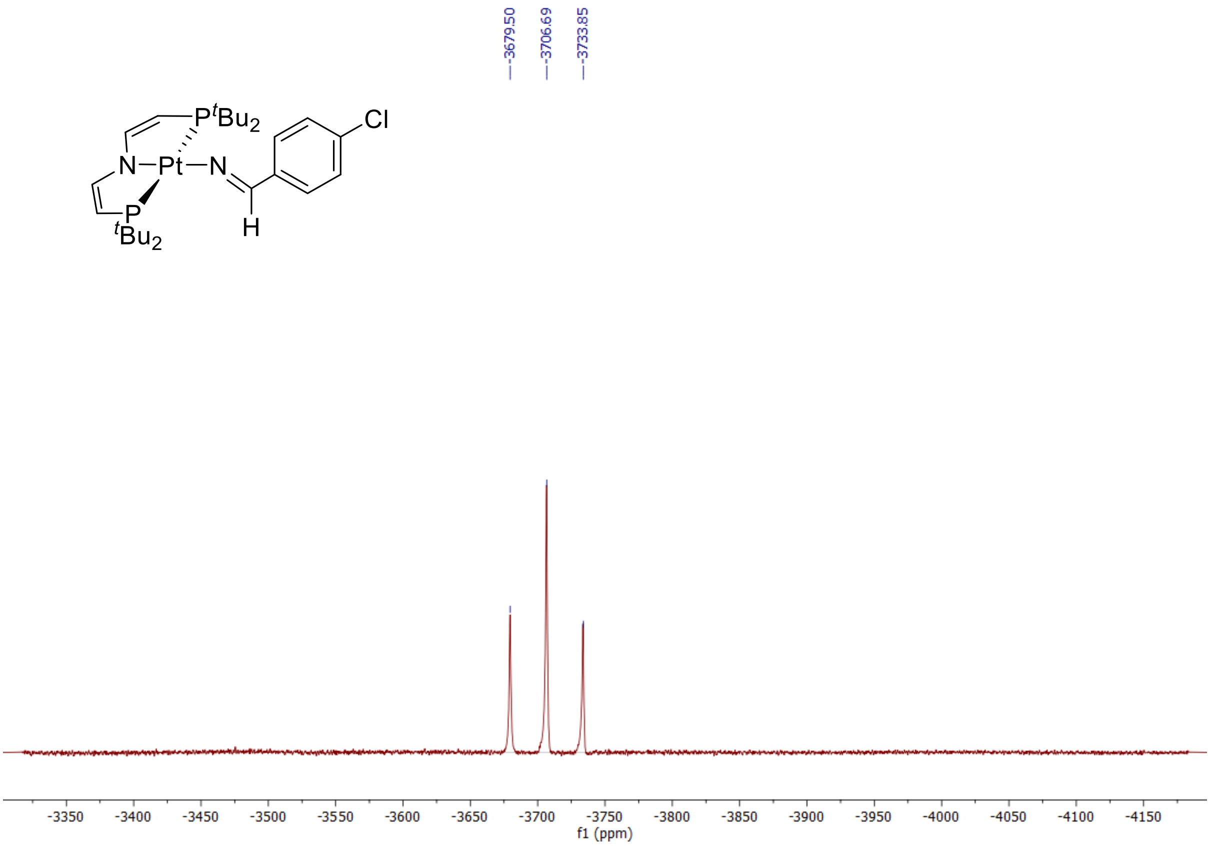

Figure S14: ^{195}Pt{^{1}H}-NMR spectrum of **3**-Cl in C_6D_6.

1.2.4 [Pt{NC(H)(C_6H_4-*p*-CF_3)}{N(CHCHP^{t}Bu$_2$)$_2$}] (**3**-CF_3)

[Pt(OTf){N(CHCHP^{t}Bu$_2$)$_2$}] (17.6 mg, 0.025 mmol, 1.0 eq.) and 4-trifluoromethylbenzonitrile (4.9 mg, 0.029 mmol, 1.1 eq.) are dissolved in THF (1.5 mL). The mixture is stirred at 25 °C for 24 hours, followed by addition of a solution of $KBEt_3H$ in THF (1 M, 26 µL, 0.026 mmol, 1.0 eq.) at –36 °C. The mixture is warmed to 25 °C and stirred for another 2 hours. Removal of the solvent and extraction with pentane (2 mL) gives a pale orange solid after solvent evaporation. Crystallization from pentane at –36°C gives a crystalline, yellow solid (10.9 mg, 60%).

^{1}H{^{31}P} NMR: (C_6D_6, 500 MHz, [ppm]): δ = 10.43 (A(X), s+d, $^3J_{HPt}$ = 88.1 Hz, 1H, NC(*H*)Ph), 7.73 (AB, d, $^3J_{HH}$ = 7.9 Hz, 2H, *o*-H), 7.51 (AB, d, $^2J_{HH}$ = 7.9 Hz, 2H, *m*-H), 6.95 (AB(X), d+dd, $^3J_{HH}$ = 5.5 Hz, $^3J_{HPt}$ = 50 Hz, 2H, NC*H*), , 4.06 (AB(X), d+dd, $^3J_{HH}$ = 5.5 Hz, $^3J_{HPt}$ = 35 Hz, 2H, PC*H*), 1.28 (s, 36H, ^{t}Bu).

^{13}C{^{1}H} NMR (C_6D_6, 126 MHz, [ppm]): δ = 162.3 (vt+dvt, $^2J_{CPt}$ = 66 Hz, $^2J_{CP}$ = 7.5 Hz, N*C*H), 159.5 (vt, $^3J_{CP}$ = 2.3 Hz, N*C*(H)Ph), 145.3 (s+d, $^3J_{CPt}$ = 116 Hz, *i*-C), 129.2 (q, $^2J_{CF}$ = 31.9 Hz, *p*-C), 126.3 (*o*-C), 125.8 (q, *m*-C, $^2J_{CF}$ = 3.7 Hz), 125.4 (q, $^1J_{CF}$ = 272 Hz, *C*F_3), 82.3 (vt, $^1J_{CP}$ = 23.2 Hz, P*C*H), 36.4 (vt, $^1J_{CP}$ = 13.1 Hz, *C*(CH_3)$_3$), 29.1 (vt, $^2J_{CP}$ = 3.0 Hz, C(*C*H_3)$_3$).

^{31}P{^{1}H] NMR (C_6D_6, 203 MHz, [ppm]): δ = 61.2 (s+d, $^1J_{PPt}$ = 2910 Hz).

^{19}F{^{1}H] NMR (C_6D_6, 471 MHz, [ppm]): δ = –61.9 (s).

^{195}Pt{^{1}H] NMR (C_6D_6, 108 MHz, [ppm]): δ = –3698 (t, $^1J_{PtP}$ = 2910 Hz).

LIFDI-MS m/z found (calc) [$C_{28}H_{45}F_3N_2P_2Pt$]$^+$: 723.2 (723.3).

Elem. Anal. found (calc) for $C_{28}H_{45}F_3N_2P_2Pt$: C 46.10 (46.47); H 6.21 (6.27); N 3.71 (3.87).

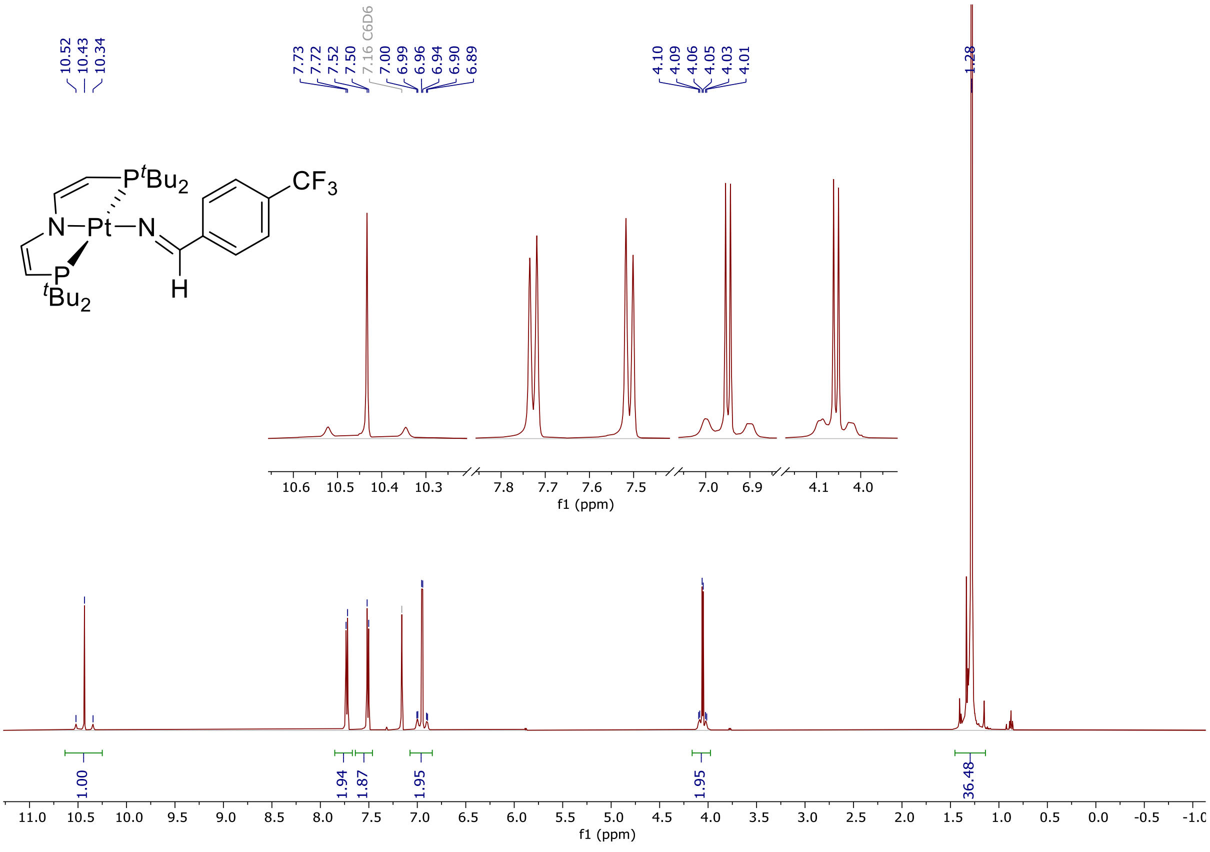

Figure S15: ^{1}H{^{31}P}-NMR spectrum of **3**-CF_3 in C_6D_6.

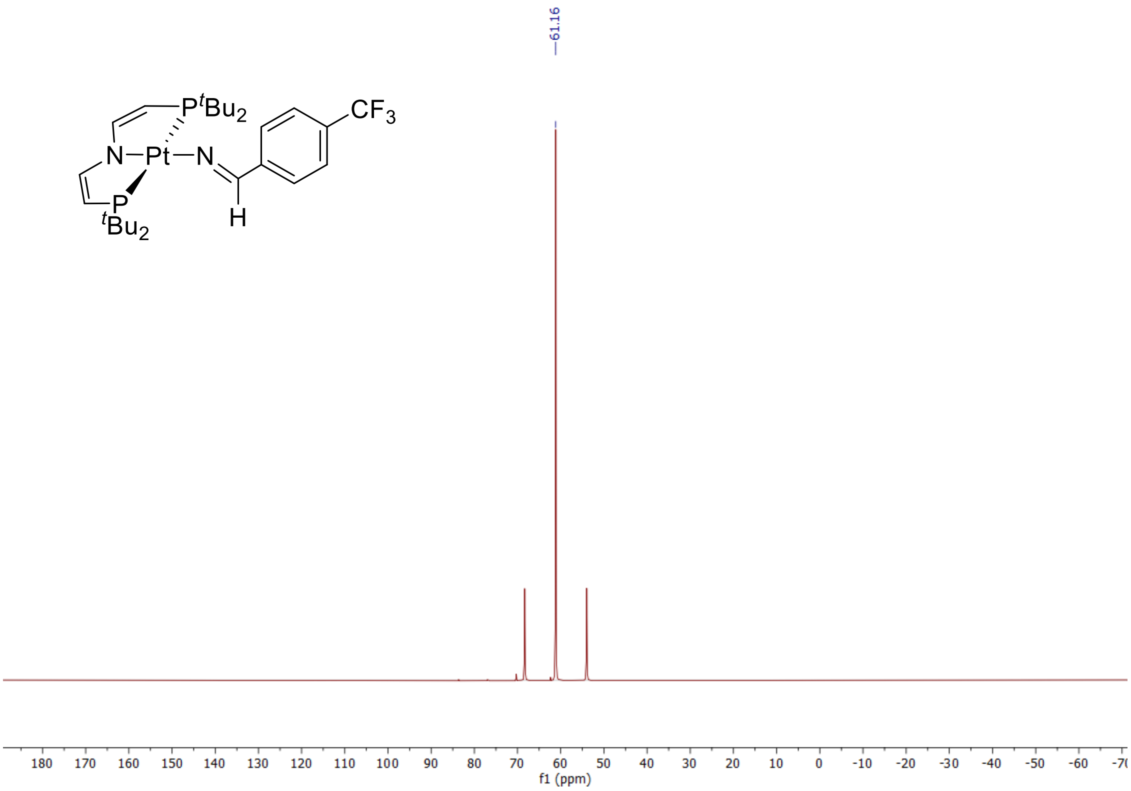

Figure S16: $^{31}P\{^1H\}$-NMR spectrum of **3**-CF_3 in C_6D_6.

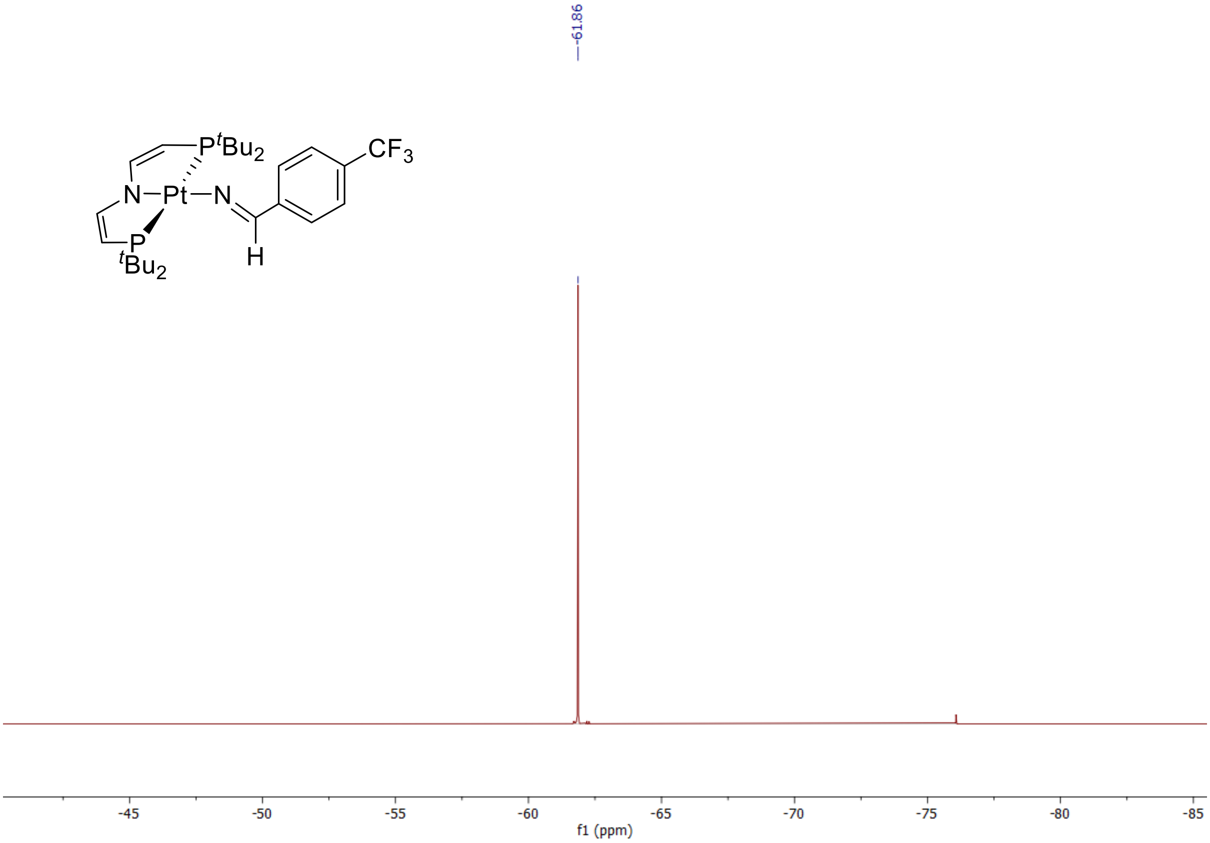

Figure S17: $^{19}F\{^1H\}$-NMR spectrum of **3**-CF_3 in C_6D_6.

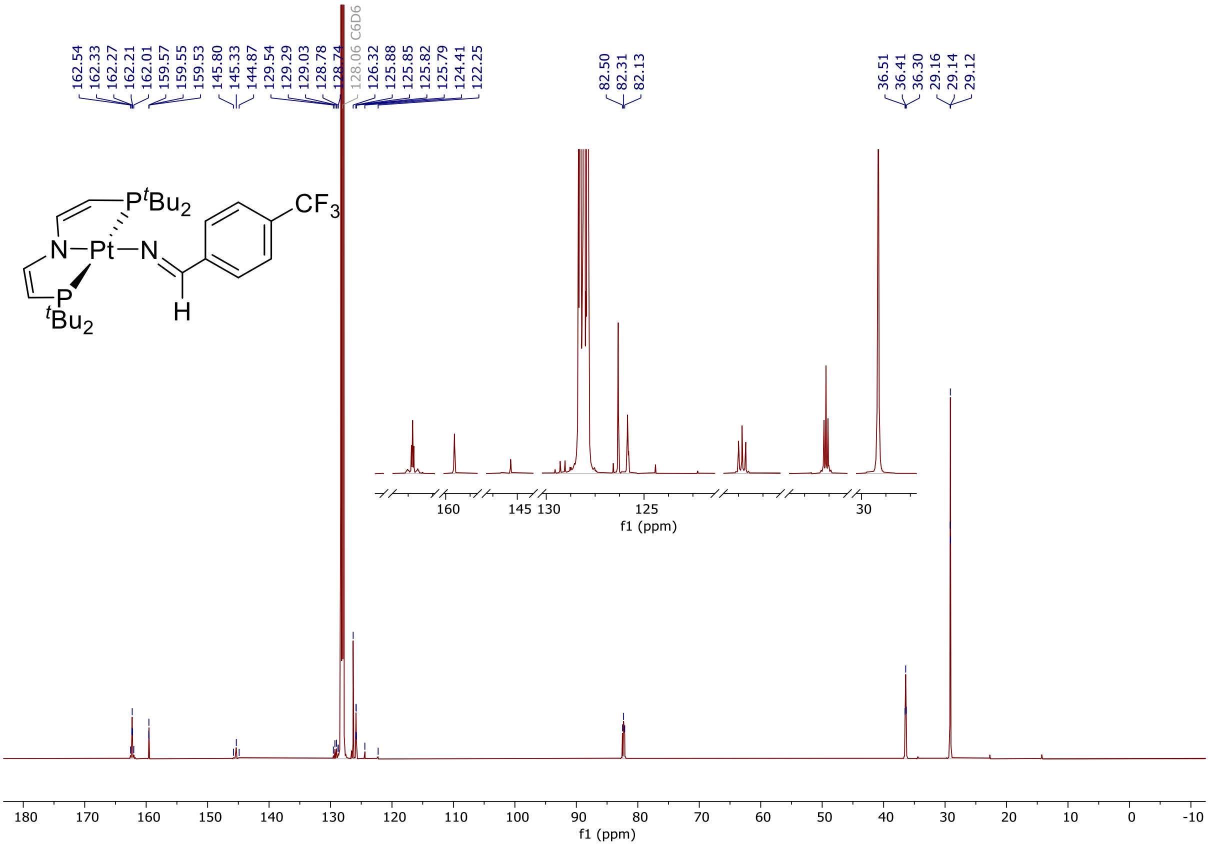

Figure S18: $^{13}C\{^1H\}$-NMR spectrum of **3**-CF_3 in C_6D_6.

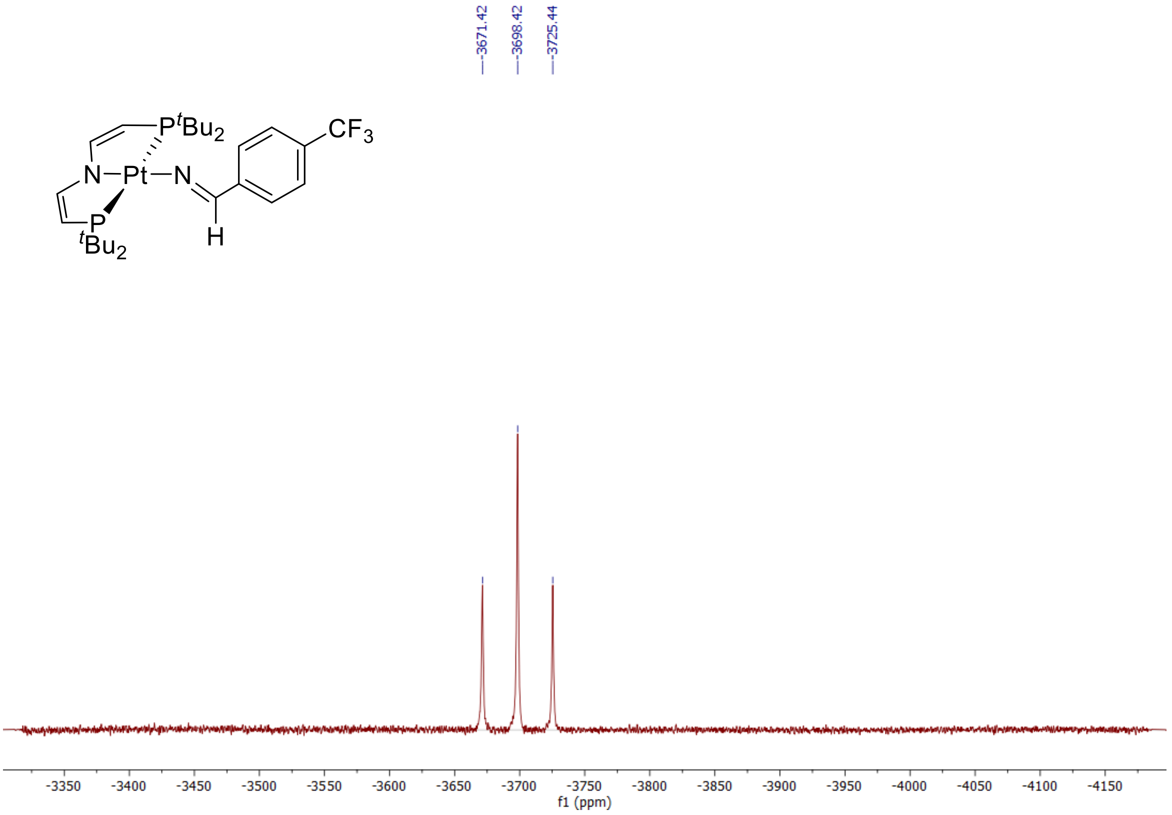

Figure S19: $^{195}Pt\{^1H\}$-NMR spectrum of **3**-CF_3 in C_6D_6.

1.2.5 [Pt{NC(H)(C_6H_4-*p*-F)}{N(CHCHP^{t}Bu$_2$)$_2$}] (**3**-F)

[Pt(OTf){N(CHCHP^{t}Bu$_2$)$_2$}] (18.7 mg, 0.027 mmol, 1.0 eq.) and 4-fluorobenzonitrile (3.6 mg, 0.030 mmol, 1.1 eq.) are dissolved in THF (1.5 mL). The mixture is stirred at 25 °C for 24 hours, followed by addition of a solution of $KBEt_3H$ in THF (1 M, 27.5 µL, 0.028 mmol, 1.0 eq.) at –36 °C. The mixture is warmed to 25 °C and stirred for another 6 hours. Removal of the solvent and extraction with pentane (3 mL) gives a pale yellow solid after solvent evaporation. Crystallization from pentane at –36°C gives a crystalline, yellow solid (9.1 mg, 51%).

^{1}H{^{31}P} NMR: (C_6D_6, 500 MHz, [ppm]): δ = 10.34 (A(X), s+d, $^3J_{HPt}$ = 88.7 Hz, 1H, NC(*H*)Ph), 7.70 (ABX, dd, $^3J_{HH}$ = 8.6 Hz, $^4J_{HF}$ = 8.6 Hz, 2H, *o*-H), 6.97 (AB(X), d+dd, $^3J_{HH}$ = 5.5 Hz, $^3J_{HPt}$ = 50 Hz, 2H, NC*H*), 6.95 (AB, d, $^2J_{HH}$ = 8.6 Hz, 2H, *m*-H), 4.07 (AB(X), d+dd, $^3J_{HH}$ = 5.5 Hz, $^3J_{HPt}$ = 35 Hz, 2H, PC*H*), 1.30 (s, 36H, ^{t}Bu).

^{13}C{^{1}H} NMR (C_6D_6, 126 MHz, [ppm]): δ = 163.0 (d, *p*-C, $^1J_{CF}$ = 245 Hz), 162.2 (vt+dvt, $^2J_{CPt}$ = 66 Hz, $^2J_{CP}$ = 7.6 Hz, N*C*H), 159.1 (vt, $^3J_{CP}$ = 1.8 Hz, N*C*(H)Ph), 139.6 (d+dd, $^3J_{CPt}$ = 116 Hz, $^4J_{CF}$ = 2.7 Hz, *i*-C), 127.6(d, $^3J_{CF}$ = 7.9 Hz, *o*-C), 115.5 (d, $^2J_{CF}$ = 21.5 Hz, *m*-C), 82.4 (vt, $^1J_{CP}$ = 23.2 Hz, P*C*H), 36.4 (vt, $^1J_{CP}$ = 13.1 Hz, *C*(CH_3)$_3$), 29.2 (vt, $^2J_{CP}$ = 3.0 Hz, C(*C*H_3)$_3$).

^{31}P{^{1}H] NMR (C_6D_6, 203 MHz, [ppm]): δ = 60.6 (s+d, $^1J_{PPt}$ = 2930 Hz).

^{19}F{^{1}H] NMR (C_6D_6, 471 MHz, [ppm]): δ = −115.6 (s).

^{195}Pt{^{1}H] NMR (C_6D_6, 108 MHz, [ppm]): δ = −3711 (t, $^1J_{PtP}$ = 2930 Hz).

LIFDI-MS m/z found (calc) [$C_{27}H_{45}FN_2P_2Pt$]$^+$: 673.3 (673.3).

Elem. Anal. found (calc) for $C_{27}H_{45}FN_2P_2Pt$: C 47.99 (48.14); H 6.77 (6.73); N 3.99 (4.16).

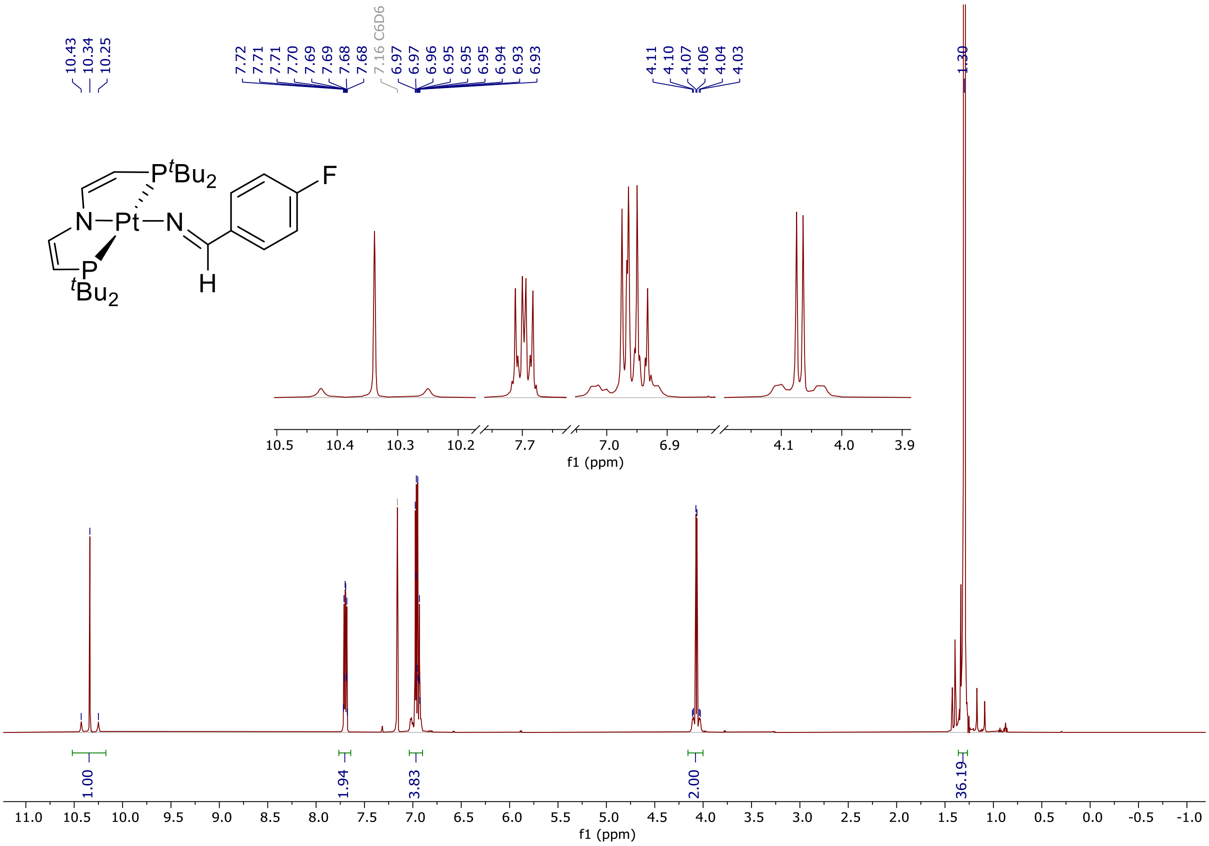

Figure S20: ^{1}H{^{31}P}-NMR spectrum of **3**-F in C_6D_6.

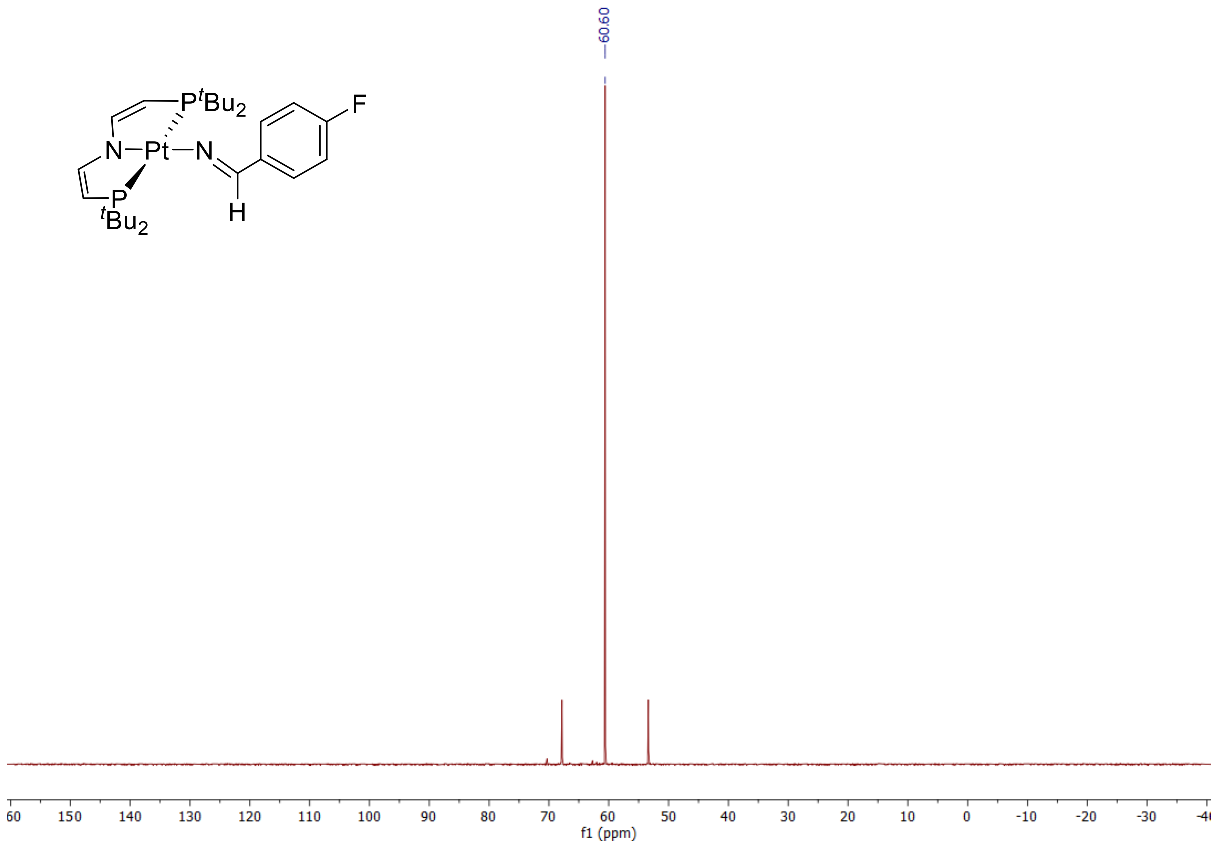

Figure S21: $^{31}P\{^1H\}$-NMR spectrum of **3**-F in C_6D_6.

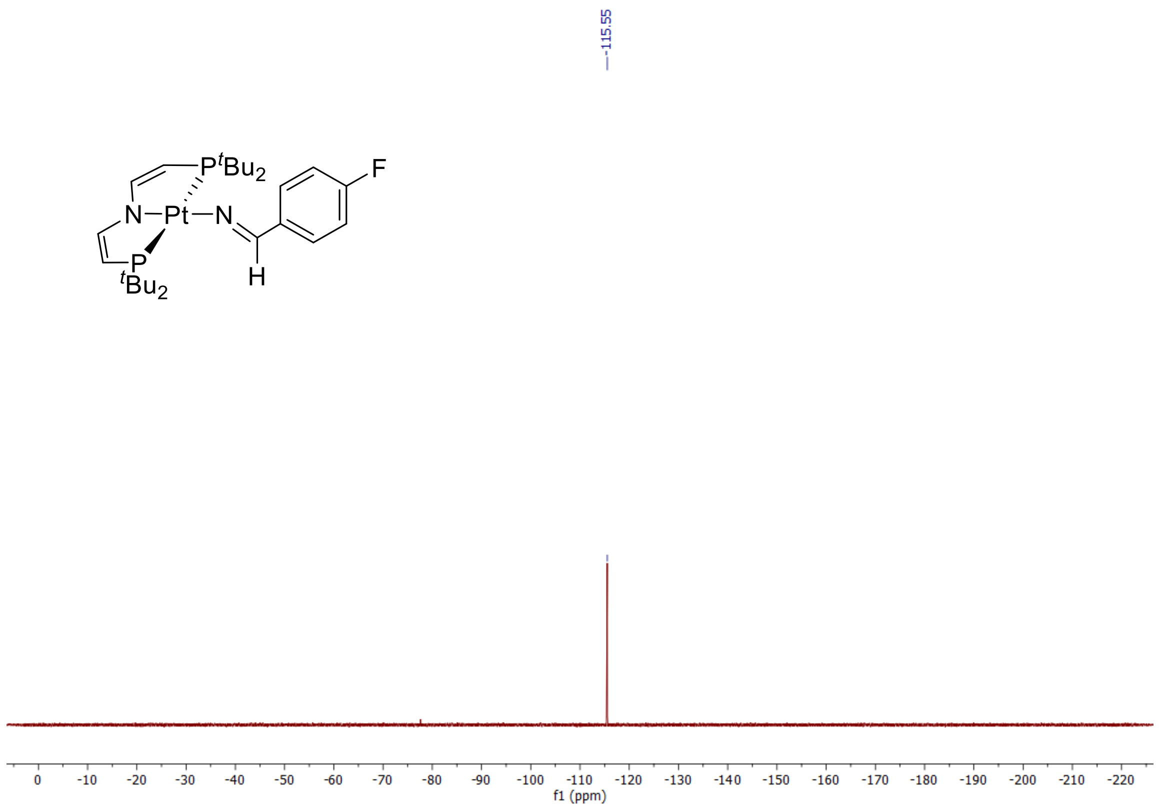

Figure S22: $^{19}F\{^1H\}$ NMR-spectrum of **3**-F in C_6D_6.

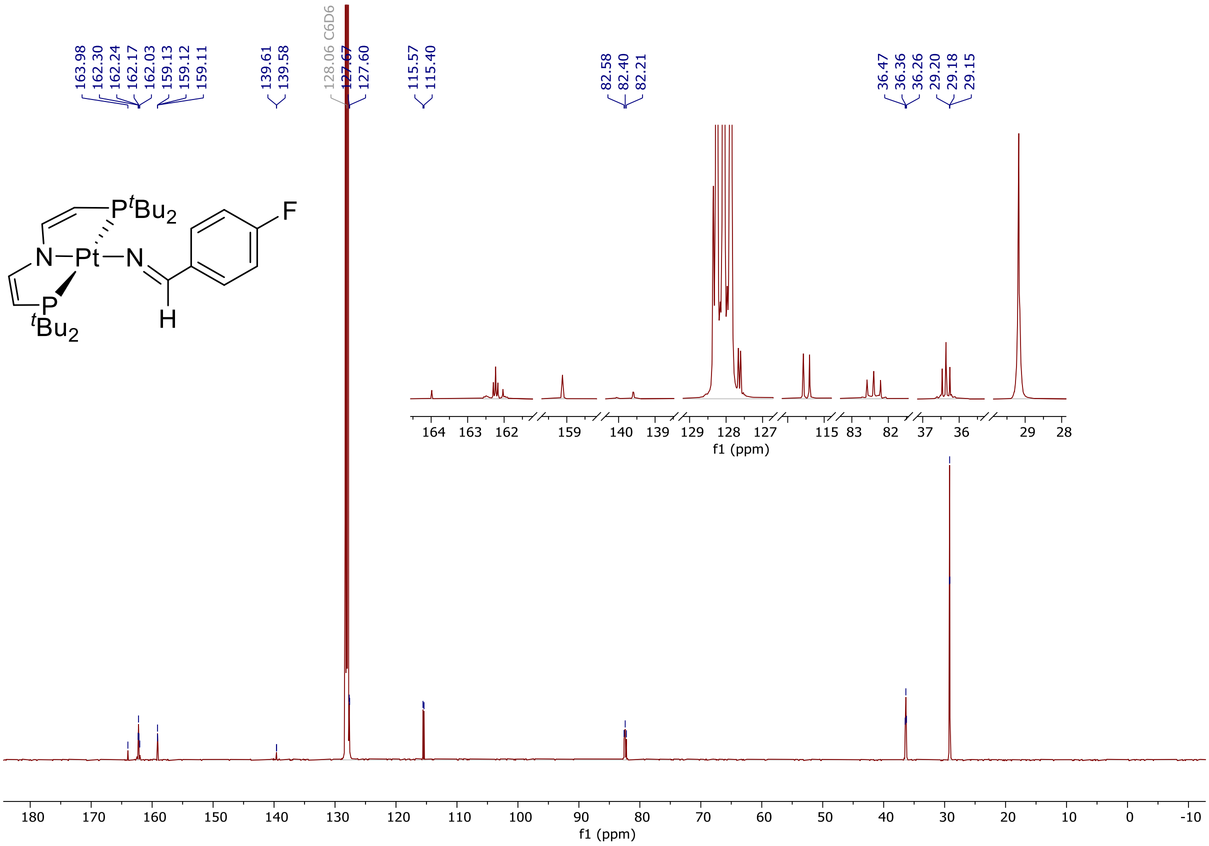

Figure S23: $^{13}C\{^{1}H\}$-NMR spectrum of **3**-F in C_6D_6.

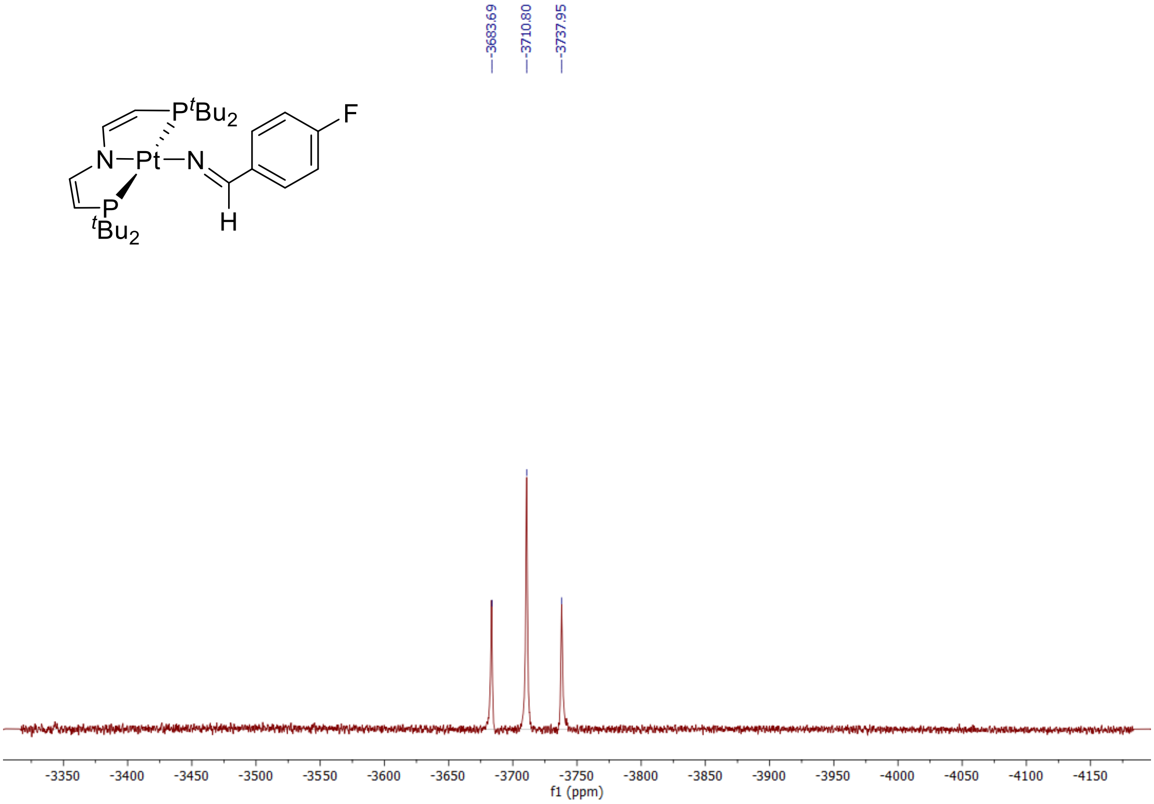

Figure S24: $^{195}Pt\{^{1}H\}$-NMR spectrum of **3**-F in C_6D_6.

1.2.6 [Pt{NC(H)(C_6H_4-^{t}Bu)}{N(CHCHP^{t}Bu$_2$)$_2$}] (**3**-^{t}Bu)

[Pt(OTf){N(CHCHP^{t}Bu$_2$)$_2$}] (17.0 mg, 0.024 mmol, 1.0 eq.) and 4-tert-butylbenzonitrile (5.0 µL, 0.030 mmol, 1.2 eq.) are dissolved in THF (1.5 mL). The mixture is stirred at 25 °C for 24 hours, followed by addition of a solution of $KBEt_3H$ in THF (1 M, 25.5 µL, 0.026 mmol, 1.1 eq.) at –36 °C. The mixture is warmed to 25 °C and stirred for another 40 minutes. Removal of the solvent and extraction with pentane (2 mL) gives a pale yellow solid after solvent evaporation. Crystallization from pentane at –36°C gives a crystalline, yellow solid (11.1 mg, 64%).

^{1}H{^{31}P} NMR: (C_6D_6, 500 MHz, [ppm]): δ = 10.52 (A(X), s+d, $^3J_{HPt}$ = 88.8 Hz, 1H, NC(*H*)Ph), 7.91 (AB, dd, $^3J_{HH}$ = 8.5 Hz, 2H, *o*-H), 7.42 (AB, d, $^2J_{HH}$ = 8.5 Hz, 2H, *m*-H), 6.99 (AB(X), d+dd, $^3J_{HH}$ = 5.6 Hz, $^3J_{HPt}$ = 50 Hz, 2H, NC*H*), 4.08 (AB(X), d+dd, $^3J_{HH}$ = 5.6 Hz, $^3J_{HPt}$ = 35 Hz, 2H, PC*H*), 1.33 (s, 36H, ^{t}Bu), 1.20 (s, 9H, ^{t}Bu).

^{13}C{^{1}H} NMR (C_6D_6, 126 MHz, [ppm]): δ = 162.2 (vt+dvt, $^2J_{CPt}$ = 66 Hz, $^2J_{CP}$ = 7.6 Hz, N*C*H), 160.8 (vt, $^3J_{CP}$ = 1.9 Hz, N*C*(H)Ph), 150.1 (*p*-C), 141.0 (*i*-C), 126.1(s, *o*-C), 125.7 (s, *m*-C), 82.5 (vt, $^1J_{CP}$ = 23.1 Hz, P*C*H), 36.4 (vt, $^1J_{CP}$ = 13.1 Hz, *C*(CH$_3$)$_3$), 34.6 (s, Ph-*C*(CH$_3$)$_3$), 31.5 (s, Ph-*C*(CH$_3$)$_3$), 29.2 (vt, $^2J_{CP}$ = 3.0 Hz, C(*C*H$_3$)$_3$).

^{31}P{^{1}H] NMR (C_6D_6, 203 MHz, [ppm]): δ = 60.3 (s+d, $^1J_{PPt}$ = 2940 Hz).

^{195}Pt{^{1}H] NMR (C_6D_6, 108 MHz, [ppm]): δ = –3714 (t, $^1J_{PtP}$ = 2940 Hz).

LIFDI-MS m/z found (calc) [$C_{31}H_{54}N_2P_2Pt$]$^+$: 711.3 (711.3).

Elem. Anal. found (calc) for $C_{31}H_{54}N_2P_2Pt$: C 52.42 (52.31); H 7.68 (7.65); N 3.89 (3.94).

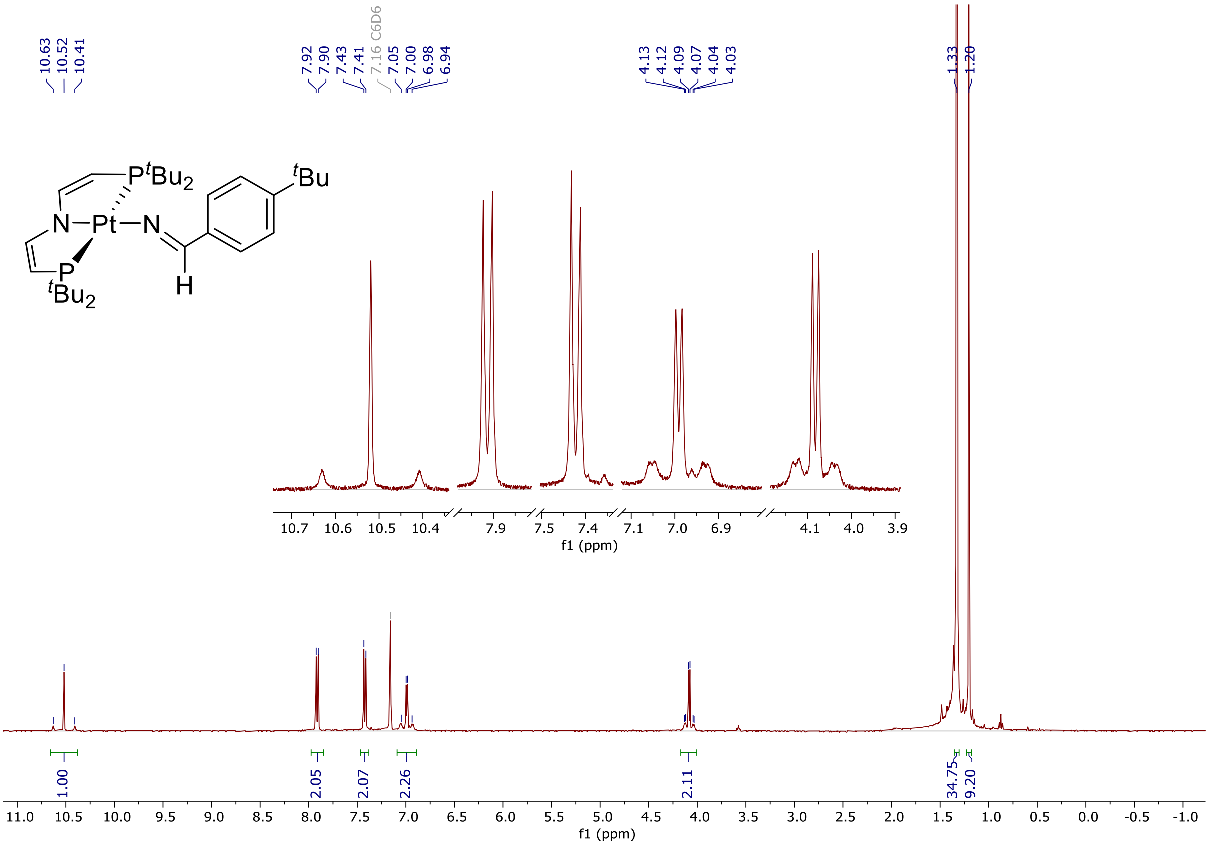

Figure S25: ^{1}H{^{31}P}-NMR spectrum of **3**-^{t}Bu in C_6D_6.

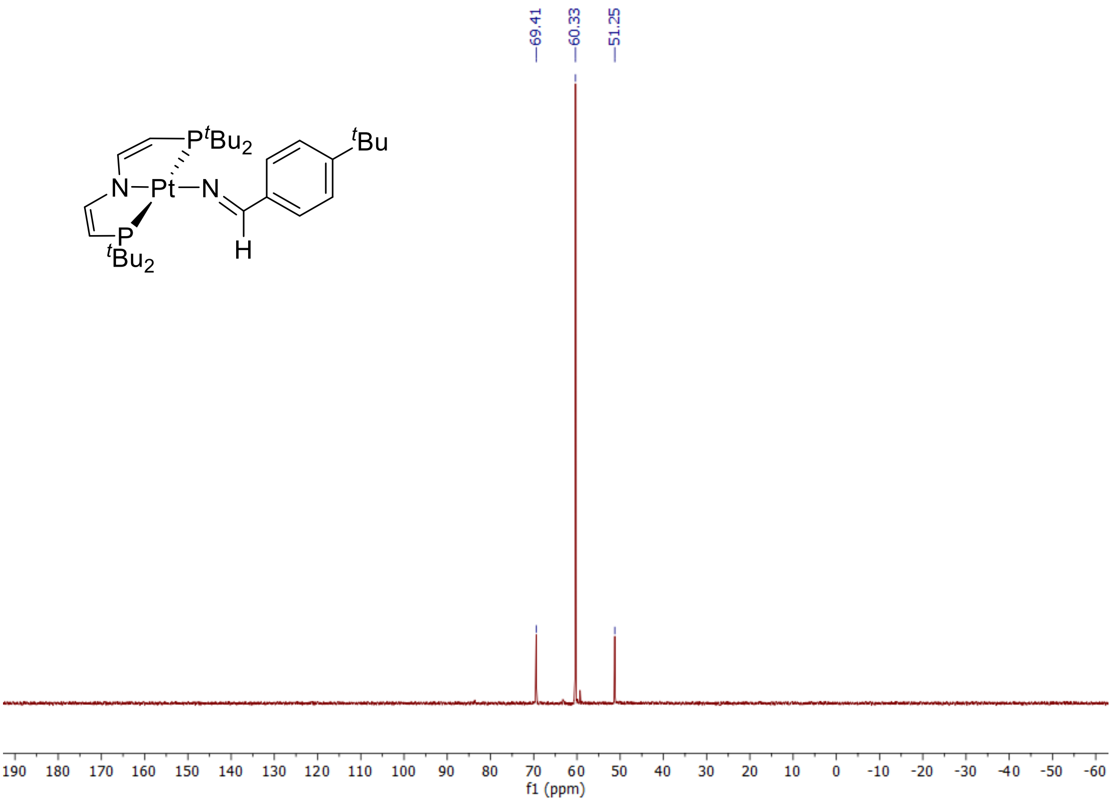

Figure S26: ^{31}P{^{1}H}-NMR spectrum of **3**-^{t}Bu in C_6D_6.

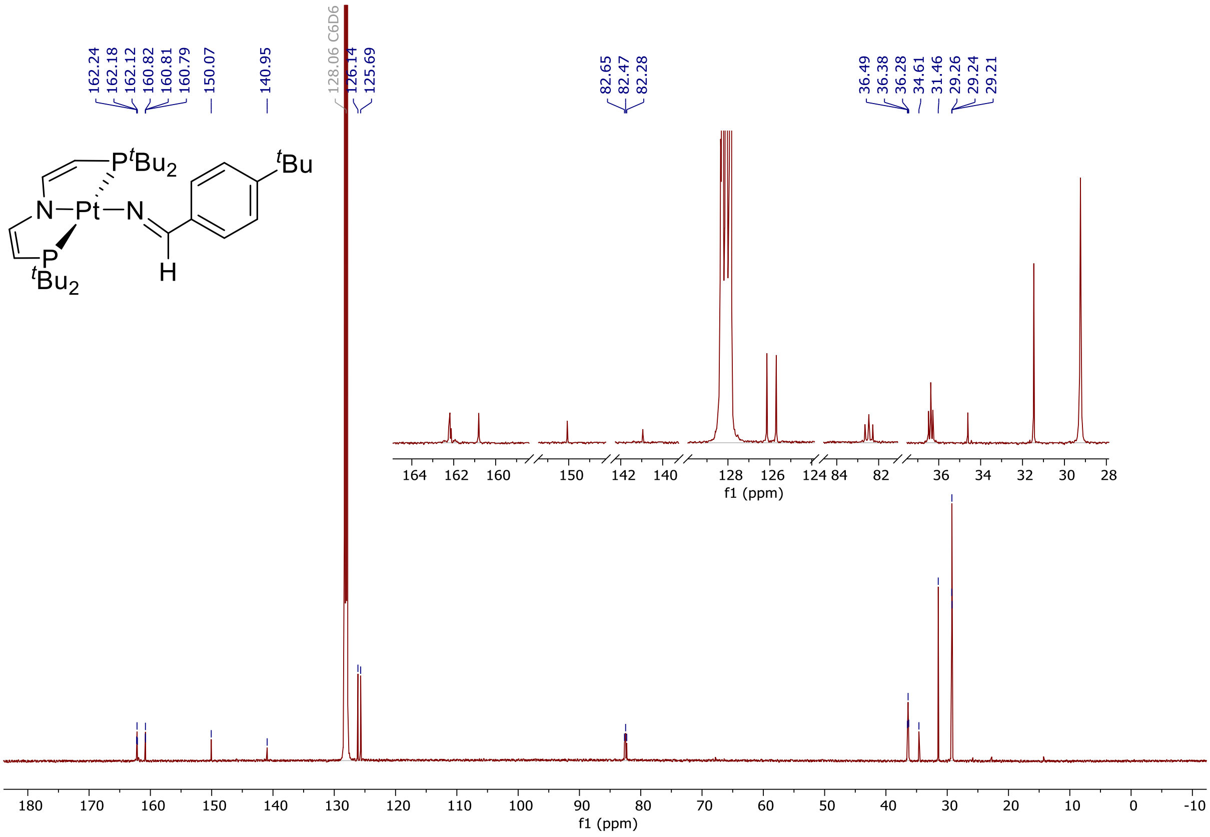

Figure S27: ^{13}C{^{1}H}-NMR spectrum of **3**-^{t}Bu in C_6D_6.

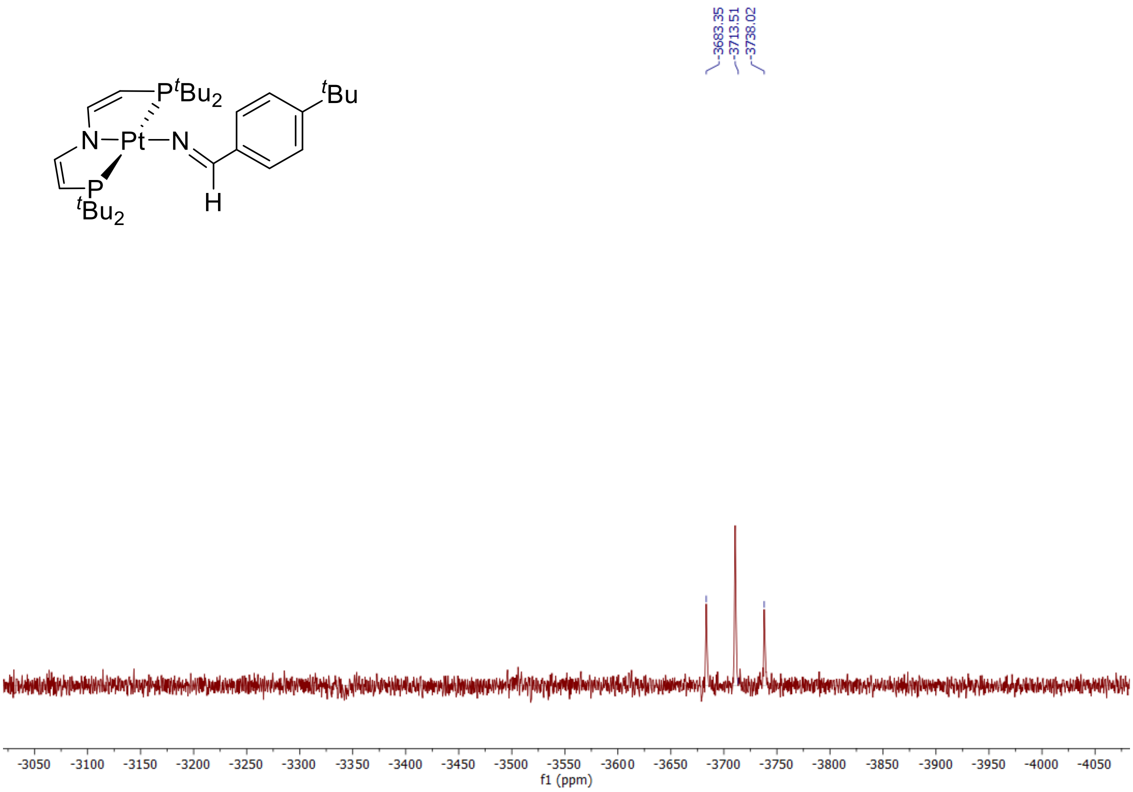

Figure S28: ^{195}Pt{^{1}H}-NMR spectrum of **3**-^{t}Bu in C_6D_6.

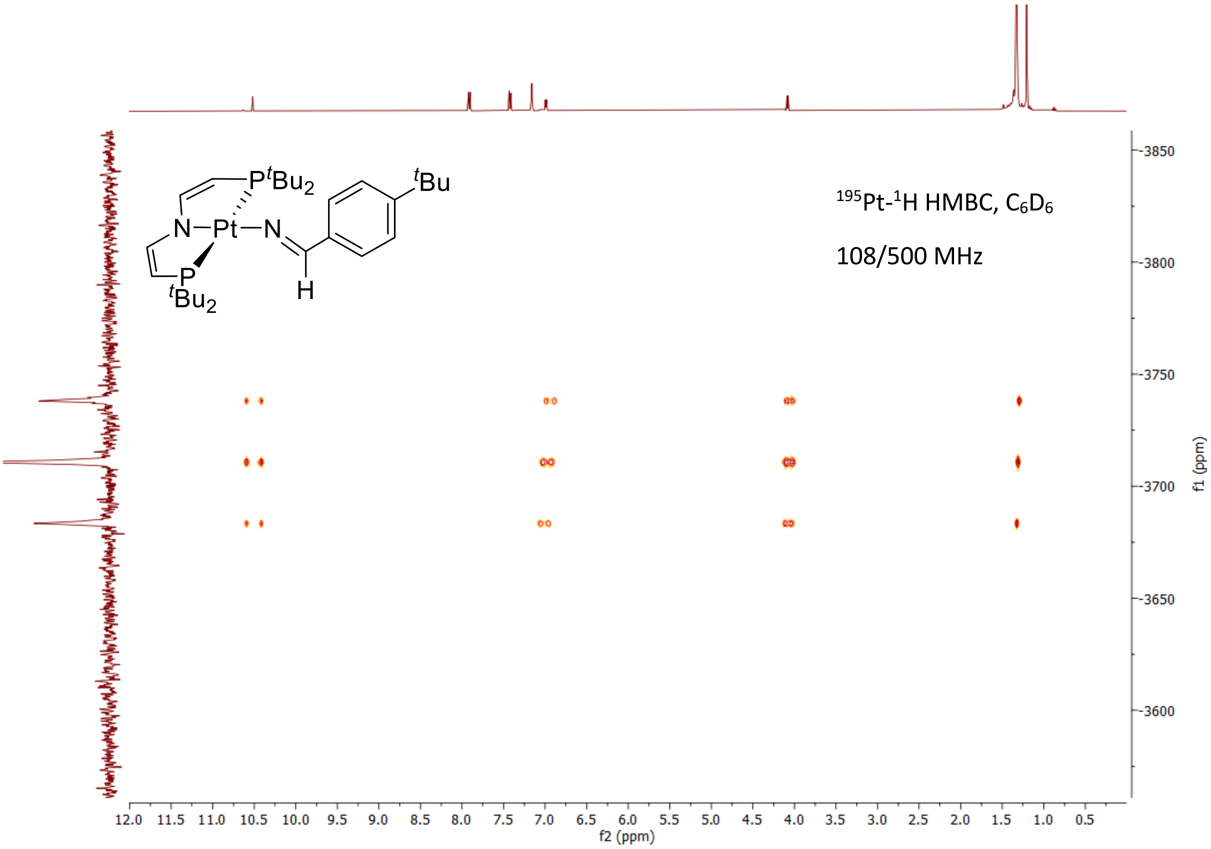

Figure S29: ^{195}Pt-^{1}H-HMBC spectrum (108/500 MHz) of **3**-^{t}Bu in C_6D_6.

1.2.7 [Pt(NCH$_2$){N(CHCHP^{t}Bu$_2$)$_2$}] (**4**)

[Pt(NH$_2$){N(CHCHP^{t}Bu$_2$)$_2$}] (10.0 mg, 0.0176 mmol, 1.0 eq.) is dissolved in 1 mL 1,4-dioxane. Paraformaldehyde (0.8 mg, 0.026 mmol, 1.5 eq.) is added and after stirring for one hour all volatiles are removed *in vacuo*. The residue is extracted with pentane (3 mL). After removal of solvent *in vacuo*, the product is obtained as a colorless solid (9.5 mg, 93%).

^{1}H{^{31}P} NMR: (C$_6$D$_6$, 400 MHz, [ppm]): δ = 9.76 (A(X), s+d, $^3J_{HPt}$ = 128 Hz, 2H, NC*H*$_2$), 6.95 (AB(X), d+dd, $^3J_{HH}$ = 5.5 Hz, $^3J_{HPt}$ = 49 Hz, 2H, NC*H*), 4.06 (AB(X), d+dd, $^3J_{HH}$ = 5.5 Hz, $^3J_{HPt}$ = 36.5 Hz, 2H, PC*H*), 1.34 (s, 36H, ^{t}Bu).

^{13}C{^{1}H} NMR (C$_6$D$_6$, 101 MHz, [ppm]): δ = 161.7 (vt+dvt, $^2J_{CPt}$ = 64 Hz, $^2J_{CP}$ = 7.6 Hz, N*C*H), 154.3 (vt, $^3J_{CP}$ = 1.9 Hz, N*C*H$_2$), 82.1 (vt, $^1J_{CP}$ = 23.3 Hz, P*C*H), 36.4 (vt, $^1J_{CP}$ = 13.1 Hz, *C*(CH$_3$)$_3$), 28.9 (vt, $^2J_{CP}$ = 3.1 Hz, C(*C*H$_3$)$_3$).

^{31}P{^{1}H] NMR (C$_6$D$_6$, 121 MHz, [ppm]): δ = 59.2 (s+d, $^1J_{PPt}$ = 2980 Hz).

^{195}Pt{^{1}H] NMR (C$_6$D$_6$, 162 MHz, [ppm]): -4668 (t, $^1J_{PtP}$ = 2980 Hz).

ESI-HR-MS m/z found (calc) [C$_{21}$H$_{42}$N$_2$P$_2$Pt+H]$^+$: 580.2546 (580.2546).

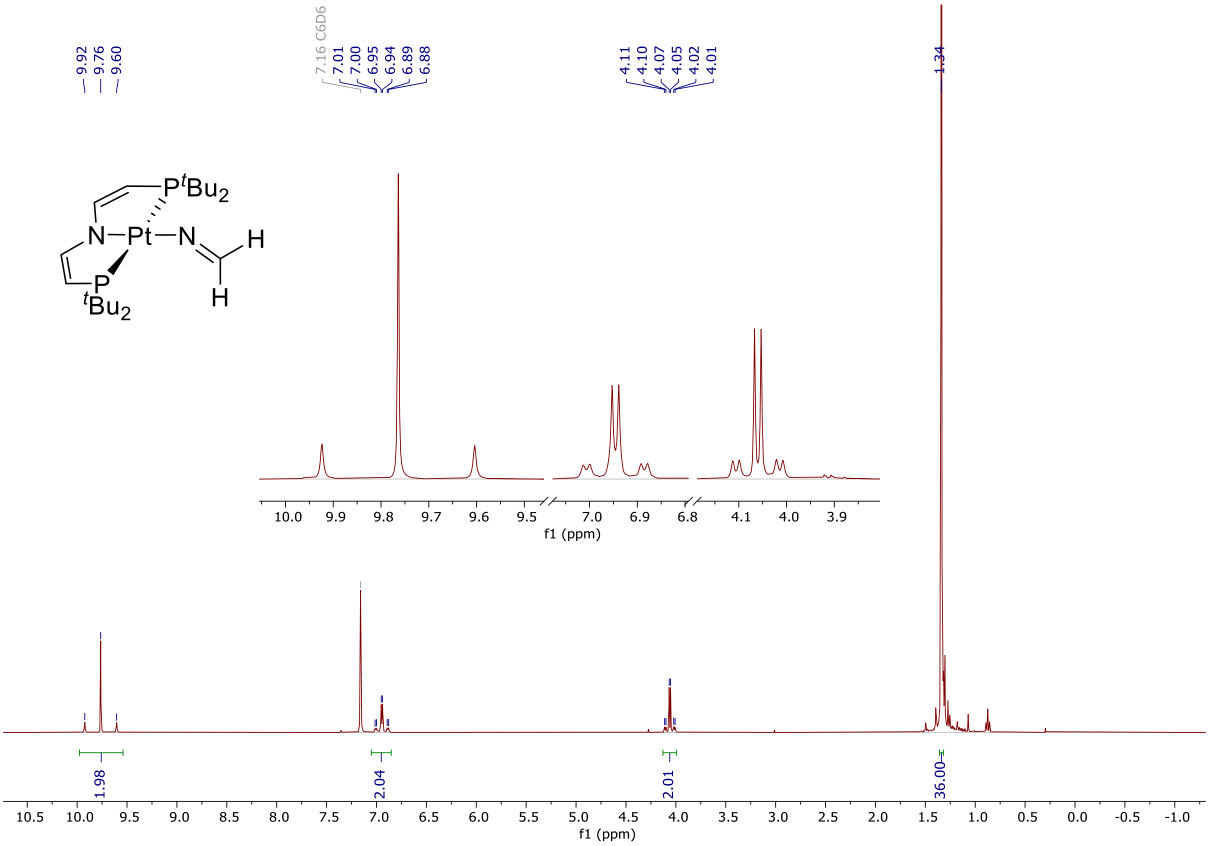

Figure S30: ^{1}H{^{31}P}-NMR spectrum of **4** in C$_6$D$_6$.

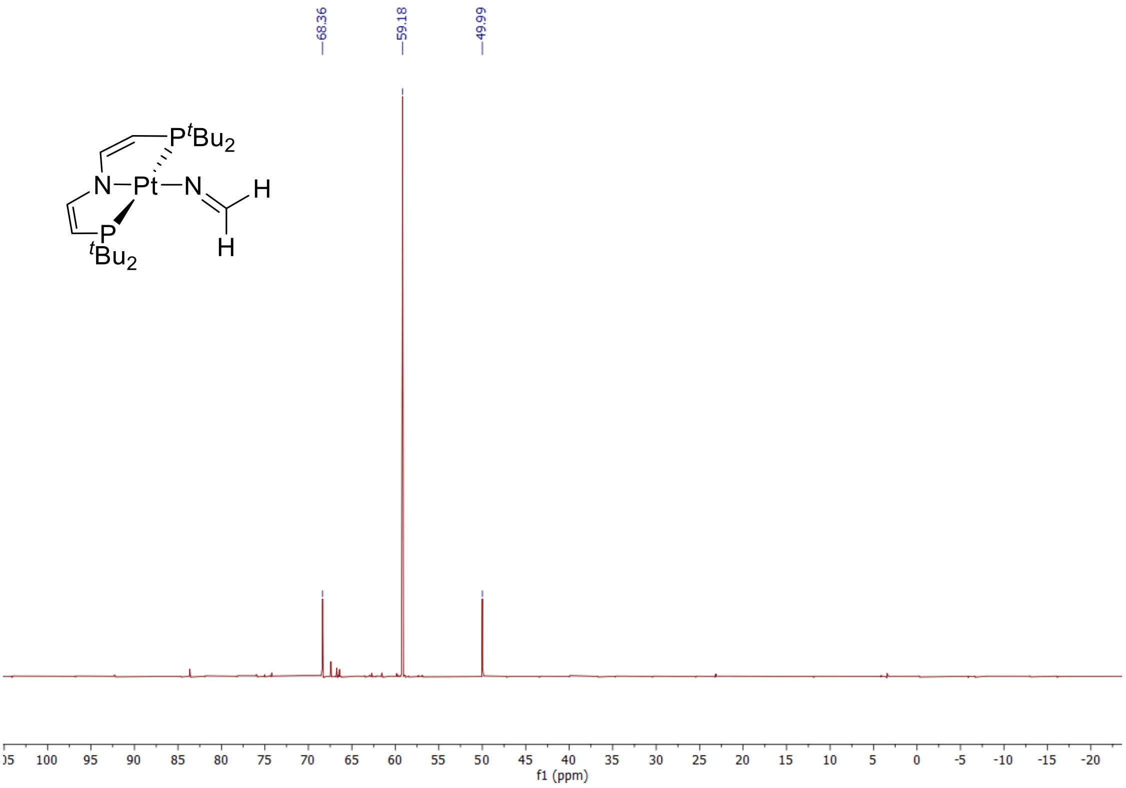

Figure S31: $^{31}P\{^1H\}$-NMR spectrum of **4** in C_6D_6.

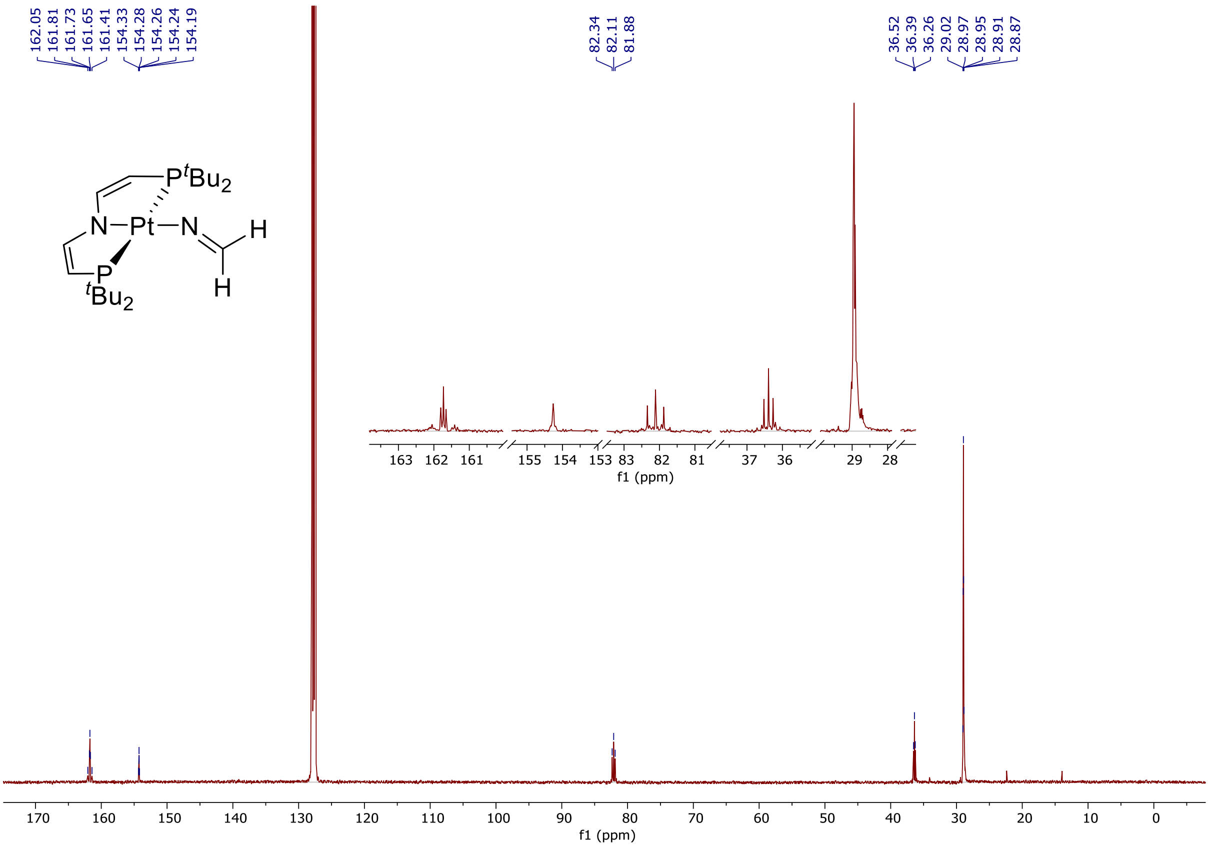

Figure S32: $^{13}C\{^1H\}$-NMR spectrum of **4** in C_6D_6.

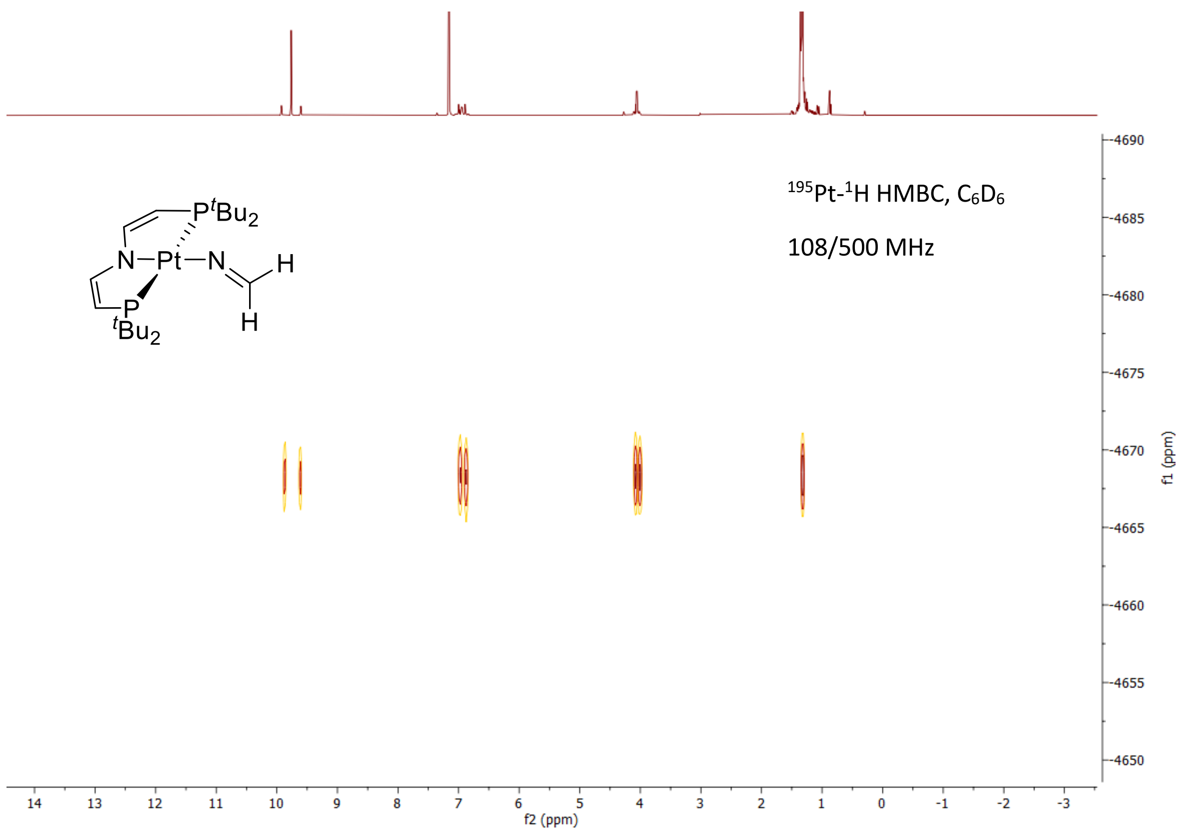

Figure S33: ^{195}Pt-^{1}H-HMBC spectrum (108/500 MHz) of **4** in C_6D_6.

1.2.8 1-Phenyl-1-CD_3-ethene[4,5]

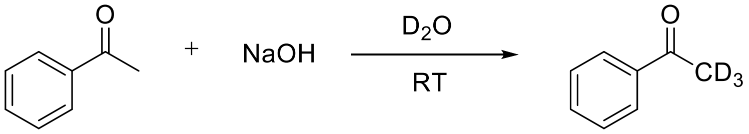

The synthetic protocol was adapted from ref. 4. A mixture of acetophenone (2.9 g, 24 mmol), KOH (0.10 g, 2.0 mmol), and D_2O (99.95% D, 16 mL, 800 mmol) is stirred at room temperature for 24 h under Ar. Afterwards, the mixture is diluted with dry diethyl ether (20 mL). After phase separation, the combined organic layers are dried with anhydrous $MgSO_4$, filtered, and concentrated. The crude product is purified by column chromatography using hexane/ethylacetate (95/5). D_3-acetophenone is obtained with 97 % deuteration as calculated by the ratio of residual CH_3 ^1H NMR signal vs. aromatic protons.

^{1}H NMR: ($CDCl_3$, 400 MHz, [ppm]): δ = 7.96 (d, $^3J_{HH}$ = 7.5 Hz, 2H, *o*-H), 7.57 (t, $^3J_{HH}$ = 7.5 Hz, 1H, *p*-H), 7.47 (t, $^3J_{HH}$ = 7.5 Hz, 1H, *m*-H), 2.57 (s, 0.1H, CH_3).

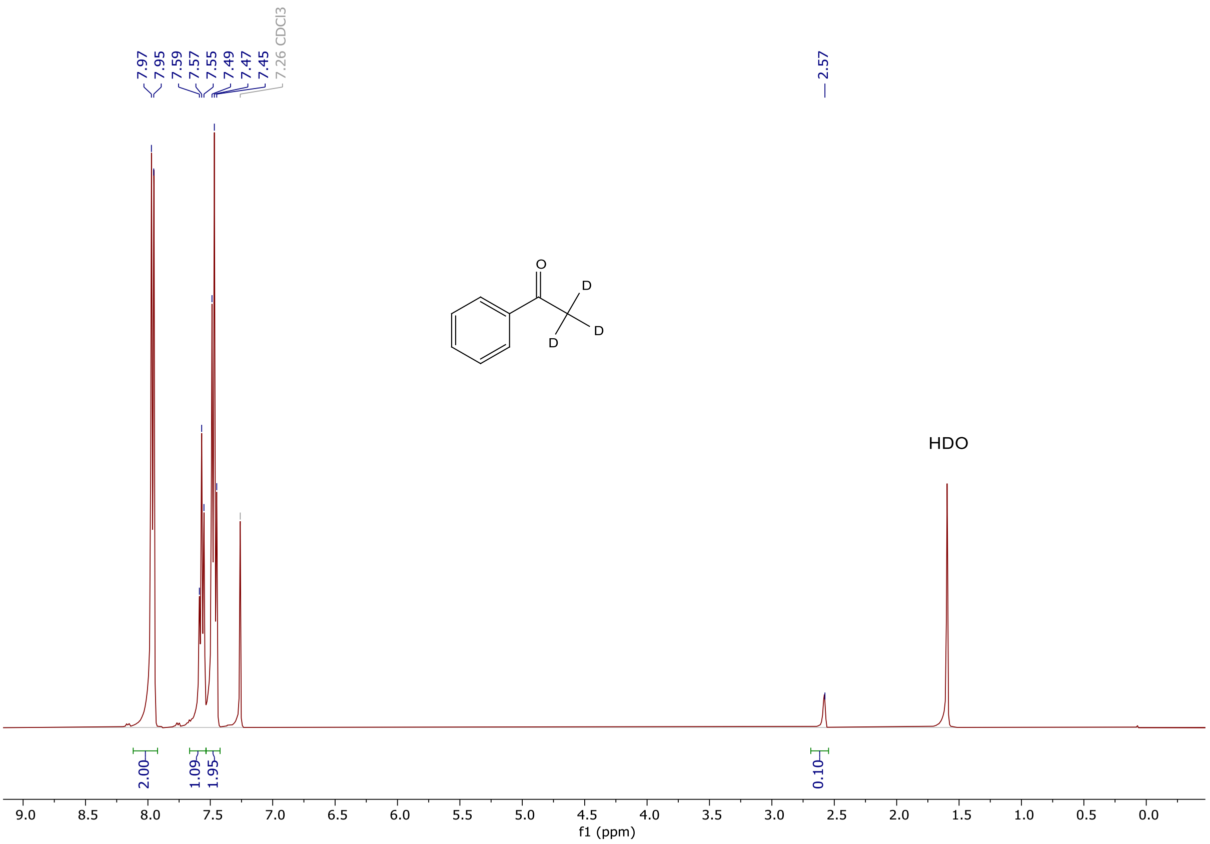

Figure S34: ^{1}H-NMR spectrum of D_3-acetophenone in $CDCl_3$. The degree of deuteration was calculated by integration of the residual CH_3 peak.

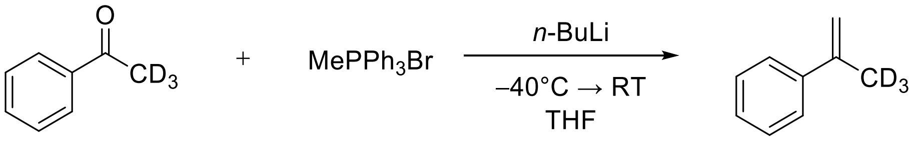

The synthetic protocol was adapted from ref. 5. *n*-Butyl lithium in hexane (10.1 mmol, 4.1 ml) is added dropwise to a solution of methyltriphenylphosphonium bromide (3.65 g, 10.1 mmol) in THF (10 ml) in a round bottom flask at –40°C. After addition, the mixture is stirred for 30 min at –40°C. After that, D_3-acetophenone (1.26 g, 10.2 mmol) in THF (1.0 mL) is added dropwise via syringe at −40°C. The mixture is warmed to room temperature and stirred for 16 h. The reaction is quenched by the addition of water (10 mL). After filtration, the organic layer is separated, washed with brine, dried over $MgSO_4$, and concentrated by rotary evaporation (crude yield: 0.95 g). Column chromatography with hexane/diethyl ether (98:2) gives the product as a colorless oil (0.27 g, 22%) with 97 % deuteration as calculated by the ratio of residual CH_3 ^{1}H NMR signal vs. aromatic protons.

^{1}H NMR: ($CDCl_3$, 400 MHz, [ppm]): δ = 7.52-7.48 (m, 2H, *o*-H), 7.39-7.33 (m, 2H, *m*-H), 7.32-7.27 (m, 1H, *p*-H), 5.40 (d, $^2J_{HH}$ = 1.5 Hz, 1H, C*H*H), 5.11 (d, $^2J_{HH}$ = 1.5 Hz, 1H, C*H*H), 2.16 (s, 0.1H, CH_3).

^{13}C{^{1}H} NMR: ($CDCl_3$, 400 MHz, [ppm]): δ = 143.2 (*C*=CH_2), 141.3 (*i*-C), 128.3 (*m*-C), 127.4 (*p*-C), 125.5 (*o*-C), 112.4 (C=*C*H_2). The septet signal of the CD_3 group was not observed.

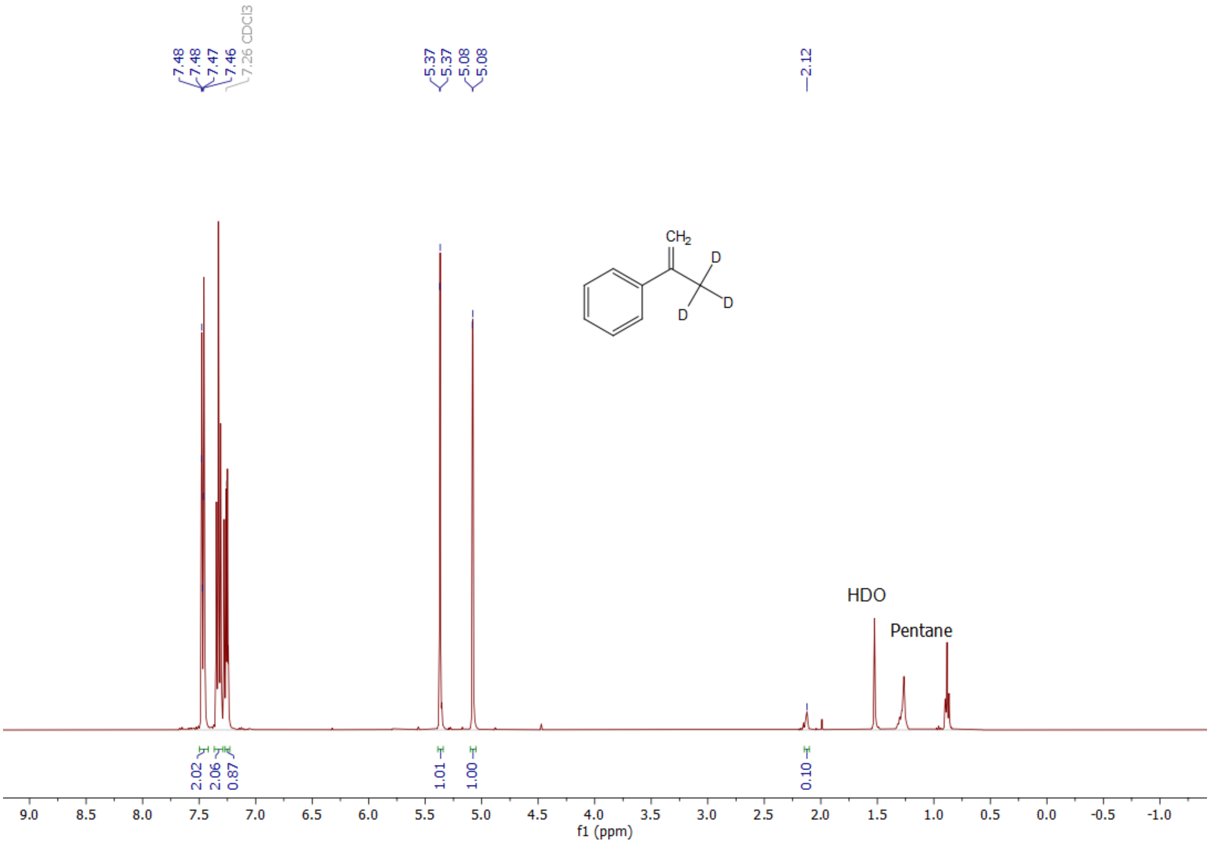

Figure S35: ^{1}H-NMR spectrum of 1-phenyl-1-CD_3-ethene in $CDCl_3$. The degree of deuteration was calculated by integration of the residual CH_3 peak.

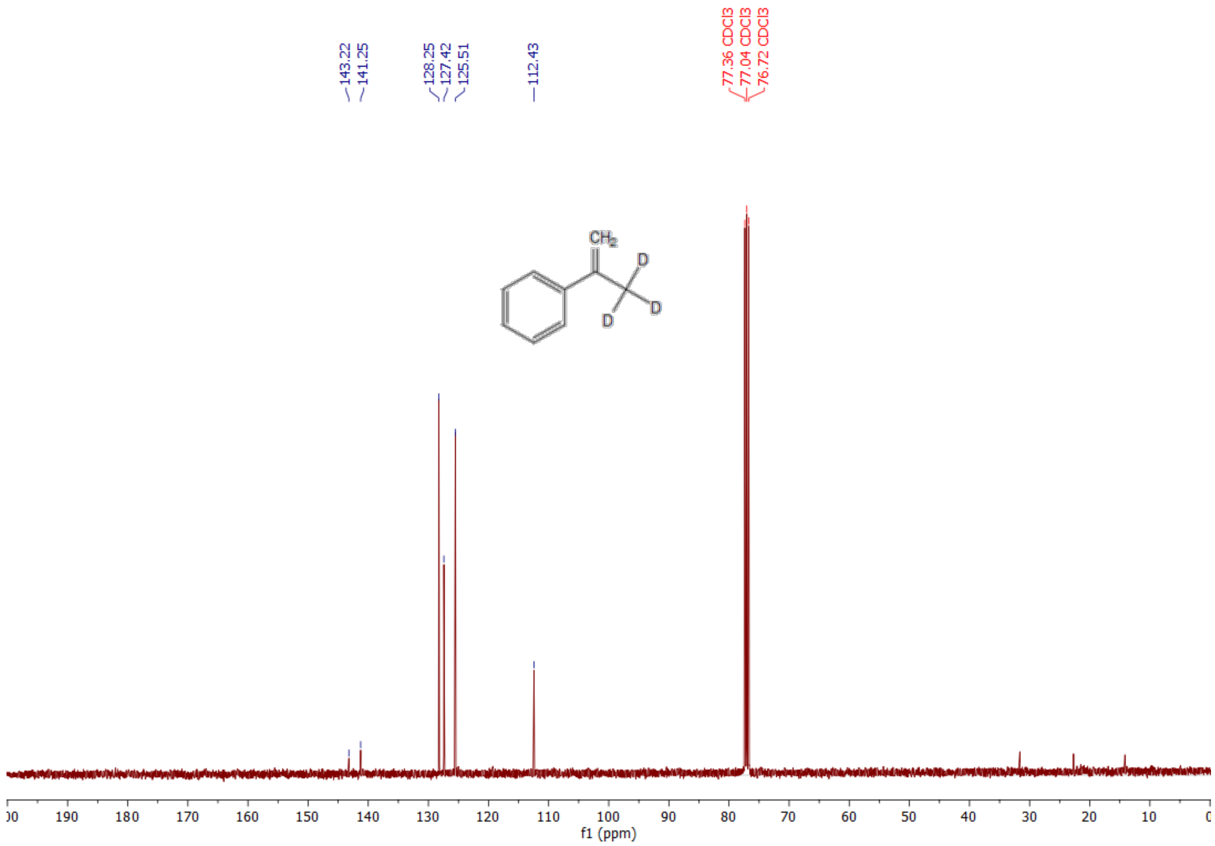

Figure S36: $^{13}C\{^{1}H\}$-NMR spectrum of 1-phenyl-1-CD_3-ethene in $CDCl_3$.

1.3 Photolysis of **1** and styrene at –30 °C

1 (4.0 mg, 6.7 µmol, 1.0 eq.) and styrene (1.1 µL, 10 µmol, 1.5 eq.) are dissolved in toluene-D8 (0.45 mL) with 1,3,5-trimethoxybenzene (0.5 eq.) as internal standard in a J-Young NMR tube. The mixture is photolyzed at −30 °C (390 nm) for 15 minutes.

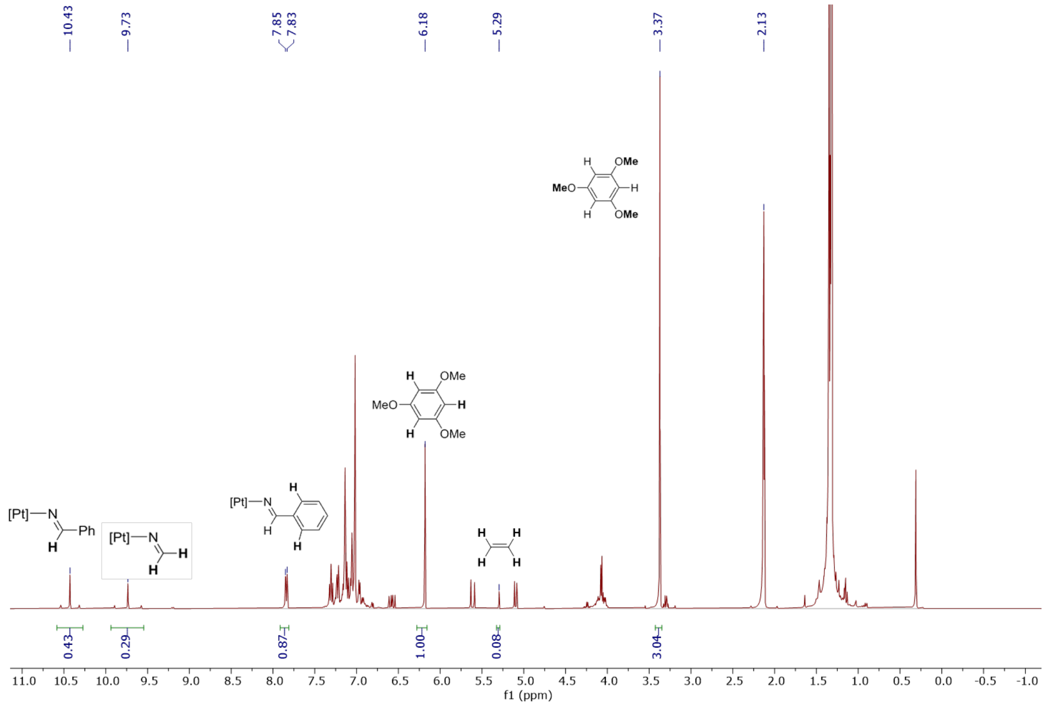

Figure S37: $^{1}H\{^{31}P\}$-NMR spectrum after photolysis of **1** and styrene in toluene-D8 with 1,3,5-trimethoxybenzene as internal standard. The imido protons around 10 ppm were used for product quantification.

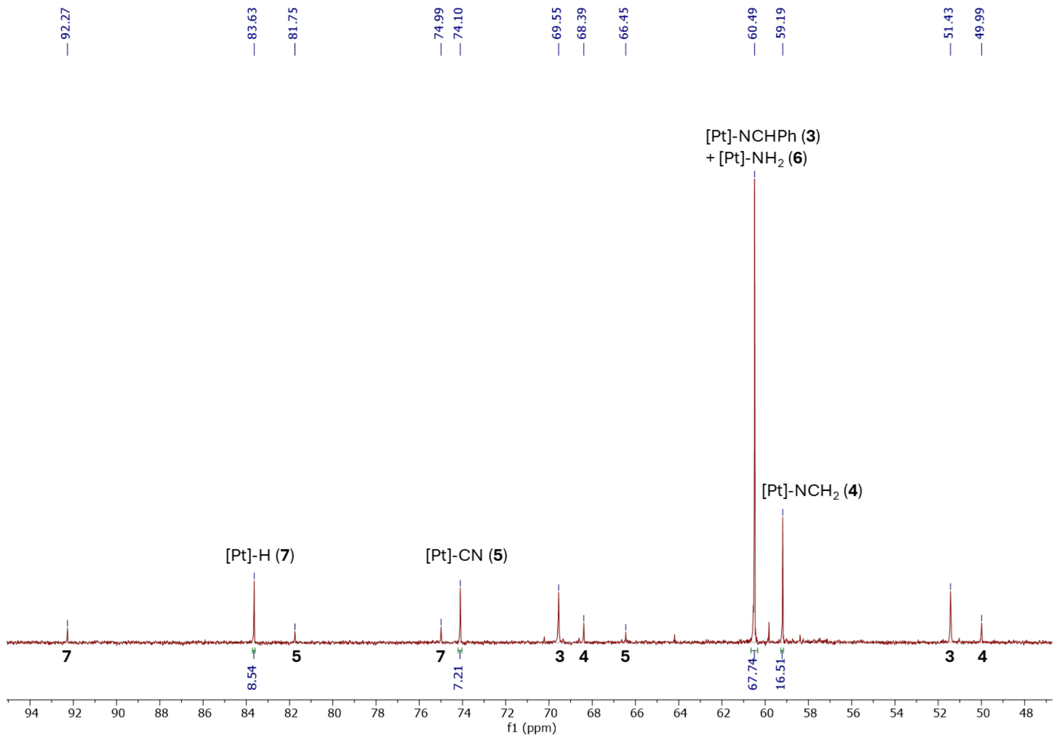

Figure S38: $^{31}P\{^{1}H\}$-NMR spectrum after photolysis of **1** and styrene in toluene-D8. ^{195}Pt satellites are labeled with the numbers of the respective compound. **6** is broadened due to hydrogen bonding and therefore overlaps with **3**.

1.4 Photolysis of **1** and styrene at –75 °C

1 (4.0 mg, 6.7 µmol, 1.0 eq.) and styrene (1.1 µL, 10 µmol, 1.5 eq.) are dissolved in toluene-D_8 (0.45 mL) with 1,3,5-trimethoxybenzene as internal standard. The mixture is photolyzed at −75 °C (380 nm) inside the NMR spectrometer until full conversion of **1** is observed, which typically takes between 1-2 hours. The sample is then warmed to room temperature. The product distribution is invariant to the excess of styrene (1-10 eq). Separation of the two main products, **3** and **8**, was not successful. The structural assignment of **8** ($\delta(^{31}P)$ = 56 ppm) therefore relies on the *in situ* NMR data (see below). DOSY NMR spectroscopy (**Figure S47**) is in line with a dinuclear species. The integral of the ^{1}H NMR signal 3.03 ppm (**Figure S39**), as well as the ^{13}C-^{1}H HSQC and ^{31}P-^{1}H HMBC spectra (**Figure S45** and **Figure S46**) support a bridging methylene group with quintet multiplicity due to coupling with four magnetically equivalent ^{31}P nuclei (**Figure S42** and **Figure S43**). The ^{195}Pt satellites ($^2J_{PtH}$ = 100 Hz) are in the typical range for Pt alkyl complexes ($^2J_{PtH}$ = 80 Hz), while the chemical shift of the bridging methylene group (3.00 ppm) is at the low-field end of the chemical shift range reported for terminal alkyl complexes in the literature (– 0.7 to 2.1 ppm).[6–8] Azide ^{15}N labelling of **1** did not perturb the NMR spectrum. When using *β*-d_2-styrene, the bridging methylene group at 3.00 ppm is not visible (**Figure S44**).

NMR characterization of **8**:

^{1}H{^{31}P} NMR: (tol-D_8, 500 MHz, [ppm]): δ = 7.18 (2H, NC*H*, overlapping with **3**), 4.16 (AB(X), d+dd, $^3J_{HH}$ = 5.4 Hz, $^3J_{HPt}$ = 46 Hz, 2H, PC*H*), 3.00 (s+d, A(X_2), $^2J_{HPt}$ = 100 Hz, CH_2), 1.41 (s, 36H, ^{t}Bu).

^{13}C{^{1}H} NMR (tol-D_8, 126 MHz, [ppm]): δ = 160.5 (vt, $^2J_{CP}$ = 7.3 Hz, N*C*H), 82.9 (vt, $^1J_{CP}$ = 24.0 Hz, P*C*H), 29.8 (vt, $^2J_{CP}$ = 3.1 Hz, C(CH_3)$_3$), 0.9 (s, CH_2).

^{31}P{^{1}H] NMR (tol-D_8, 203 MHz, [ppm]): δ = 56.1 (s+d, $^1J_{PPt}$ = 3210 Hz).

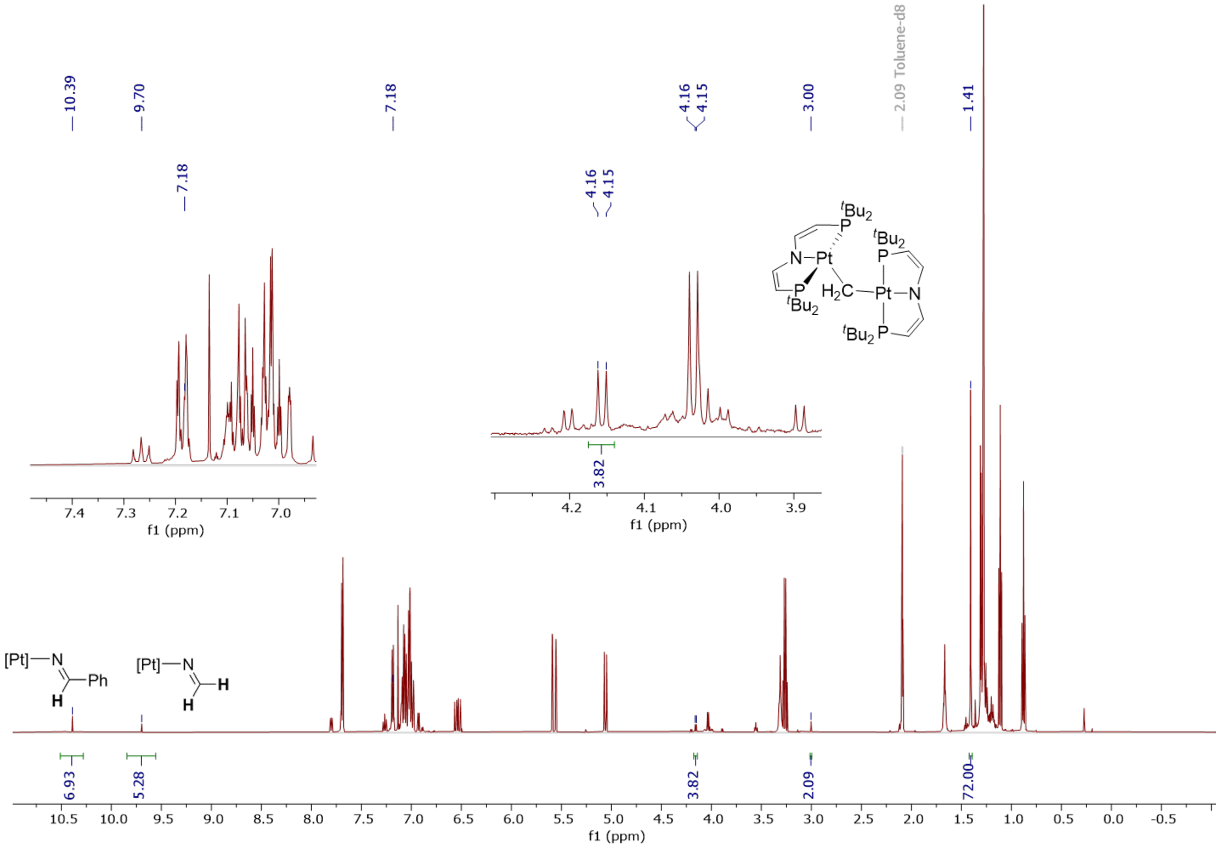

Figure S39: ^{1}H{^{31}P}-NMR spectrum of reaction of **1** with styrene after low-temperature photolysis in toluene-D_8.

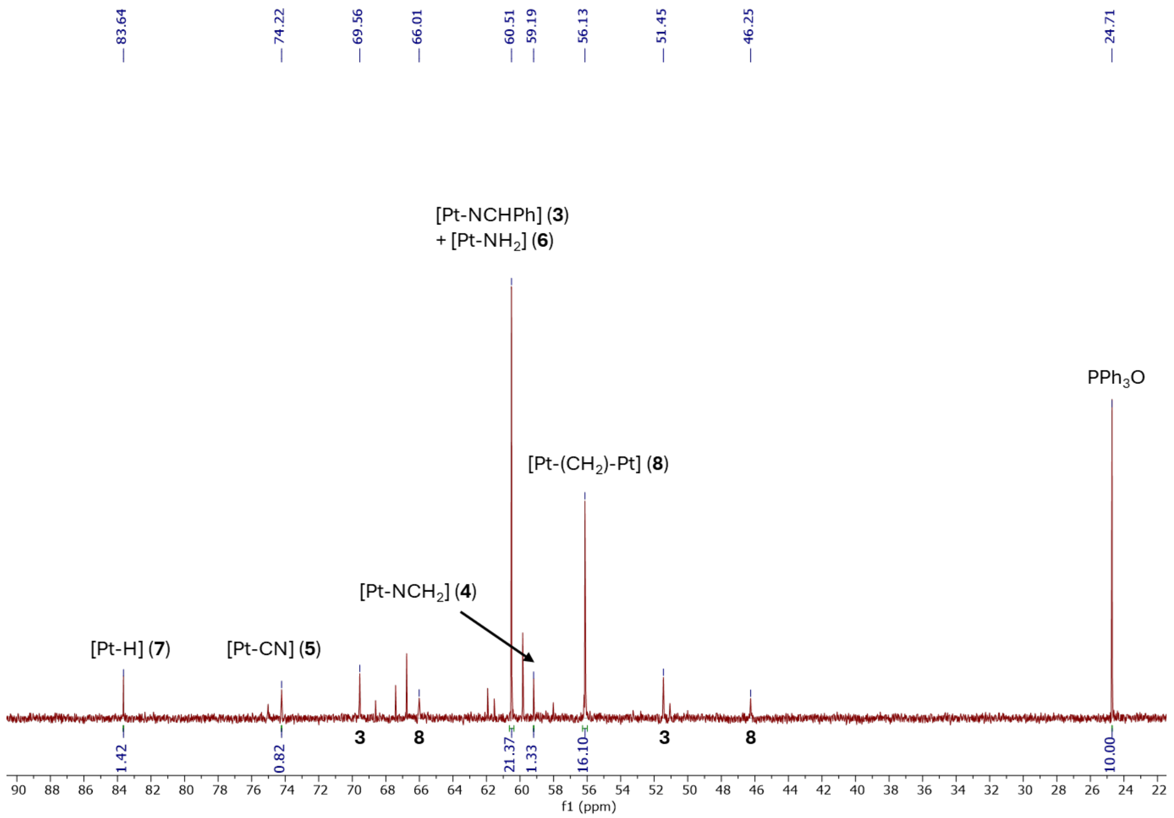

Figure S40: $^{31}P\{^{1}H\}$-NMR spectrum of reaction of **1** with styrene after photolysis at −75 °C in toluene-D$_8$ (internal standard $OPPh_3$ at δ_P = 24.7 ppm).

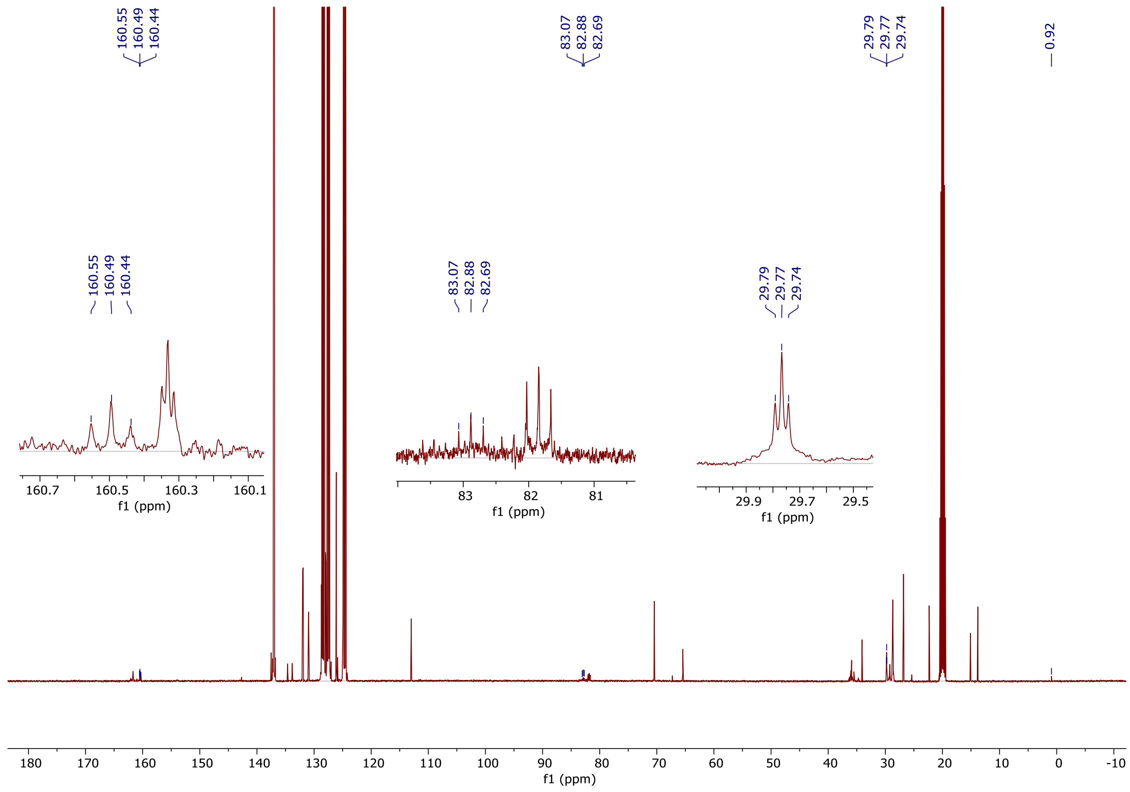

Figure S41: $^{13}C\{^{1}H\}$-NMR spectrum of reaction of **1** with styrene after photolysis at −75 °C in toluene-D$_8$.

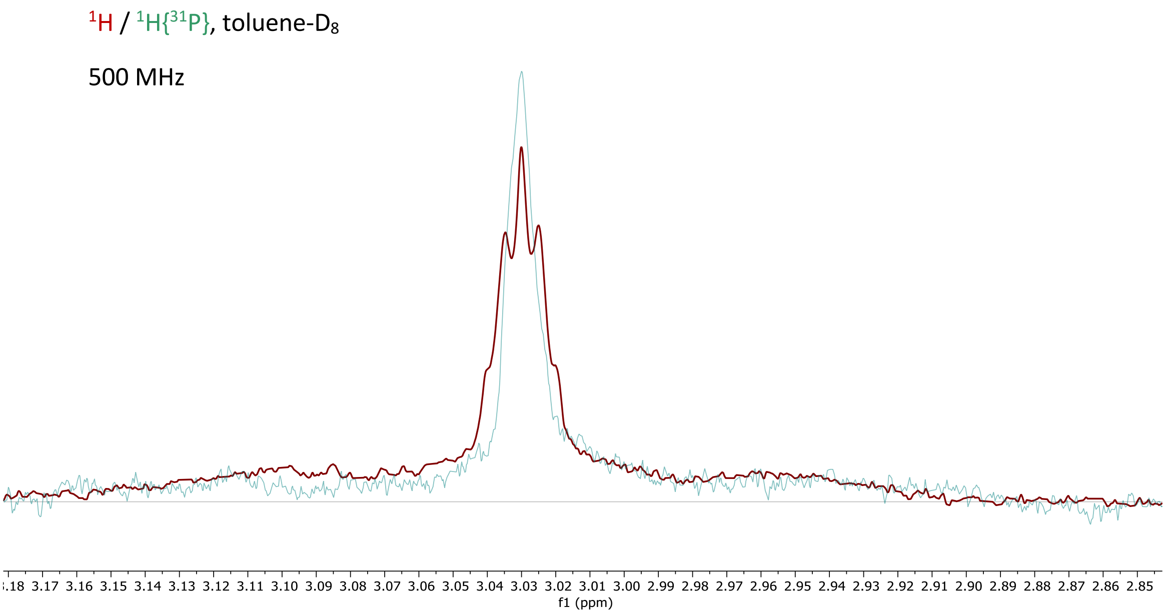

Figure S42: Expansions of the ^1H (red) and $^1H\{^{31}P\}$ (green) signals of the bridging CH_2 group in toluene-D_8.

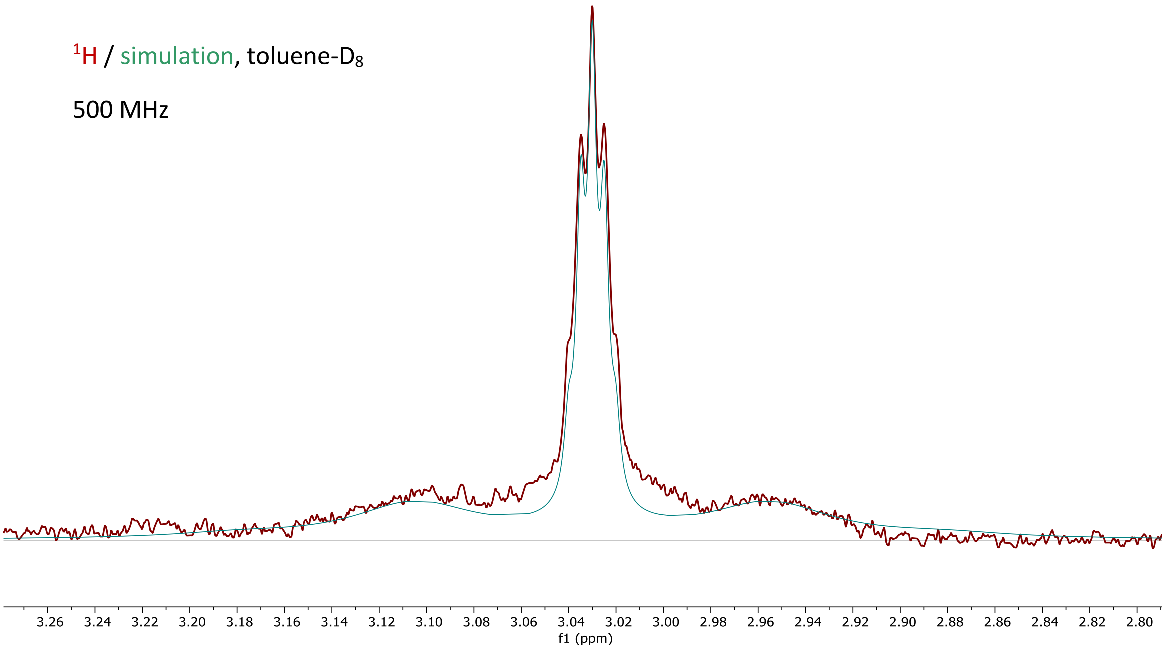

Figure S43: Simulation (green) of the ^1H-NMR signal (red) of the bridging CH_2 group. Three spin systems have been used for the simulation (X = ^{31}P; Y = ^{195}Pt considering 34% natural abundancy), i.e. $AX_2X'_2$ (46%), $AX_2X'_2Y$ (47%), and $AX_2X'_2YY'$ (7%). Coupling constants: $^3J_{HP}$ = 2.5 Hz, $^2J_{HPt}$ = 100 Hz, linewidth: 2.2 Hz (main peak) and 30 Hz (Pt satellites).

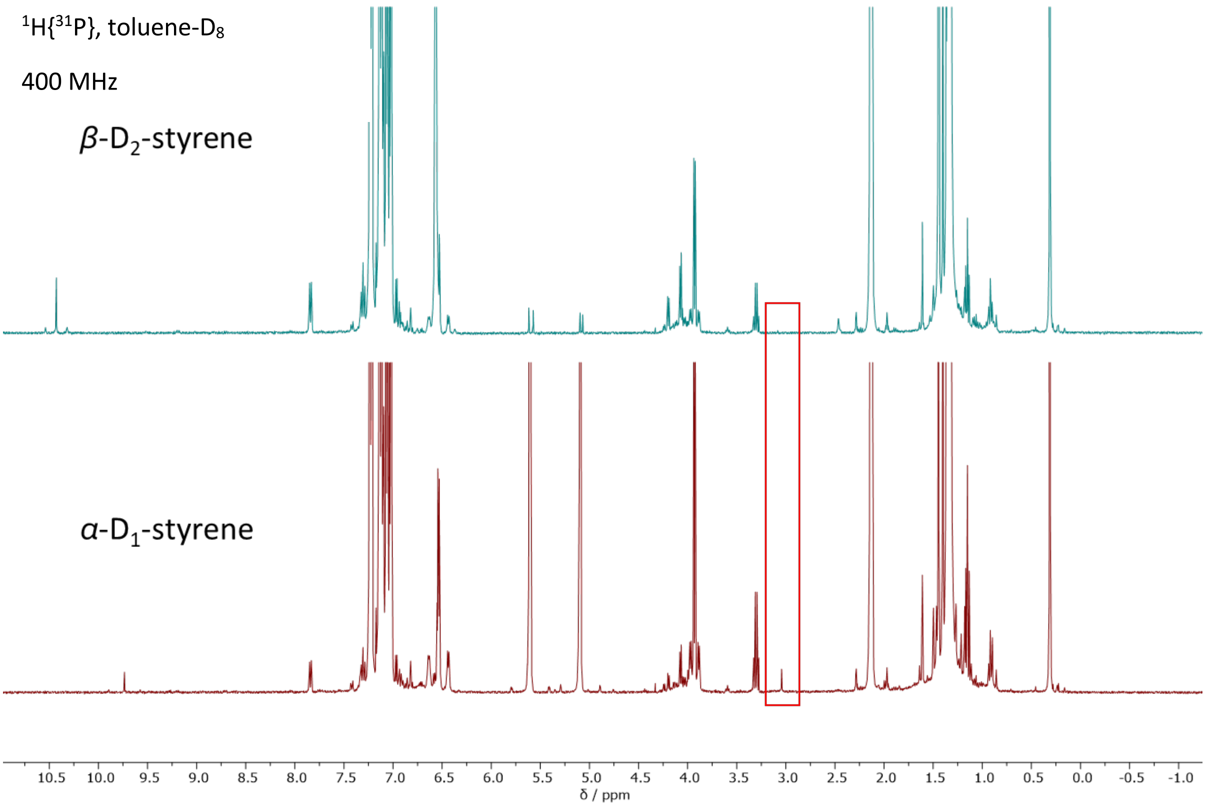

Figure S44: $^{1}H\{^{31}P\}$-NMR spectra of reaction of **1** with α-D$_1$-(bottom) and β-D$_2$-styrene (top) after photolysis at −75 °C in toluene-D$_8$. The peak at 3.0 ppm is not visible when using β-D$_2$-styrene.

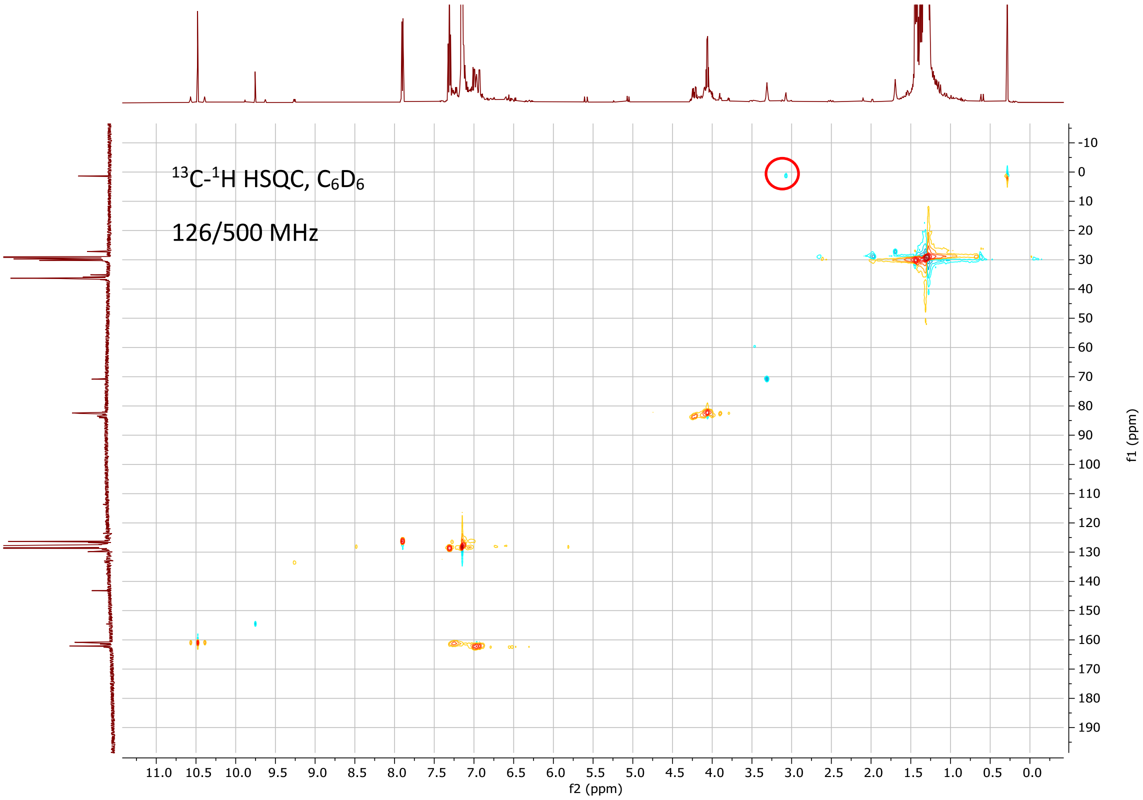

Figure S45: ^{1}H–^{13}C-HSQC spectrum (500/126 MHz) after evaporating all volatiles and redissolving in C_6D_6. The red circle marks the bridging CH_2 group.

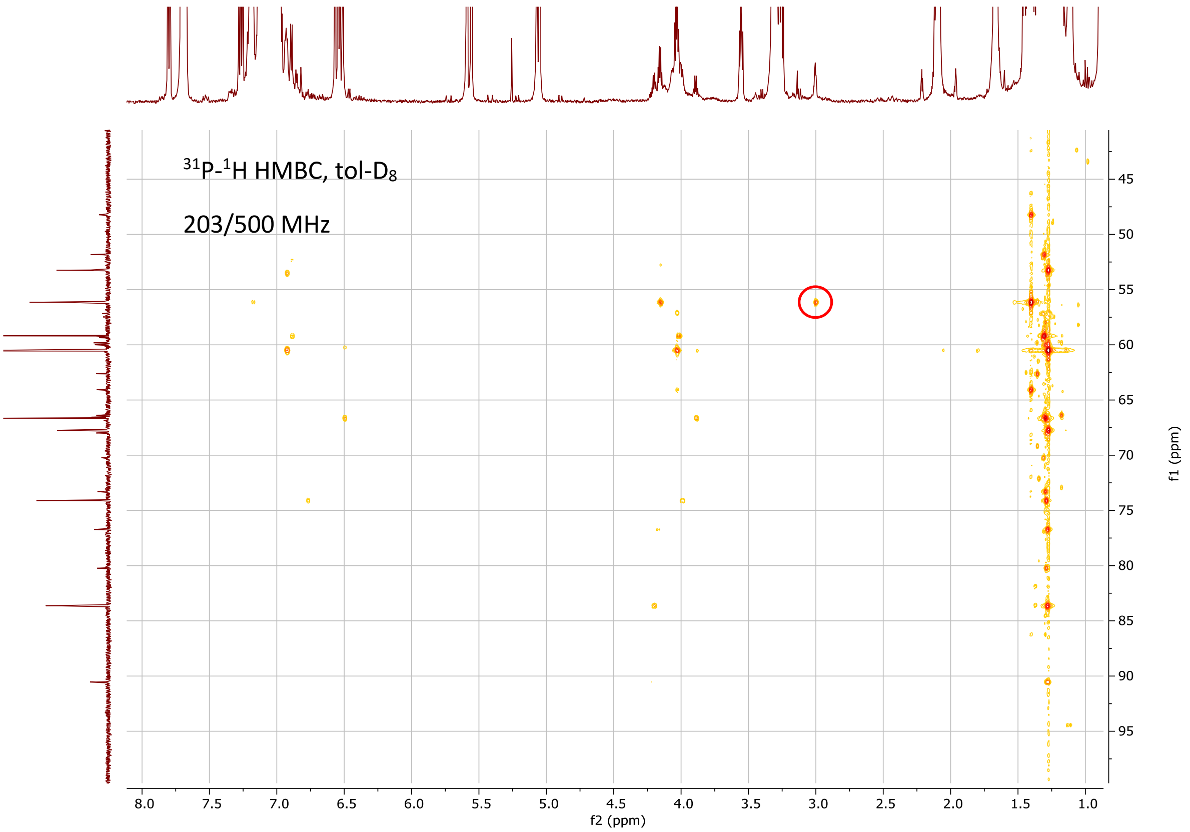

Figure S46: ^{1}H–^{31}P-HMBC spectrum after the reaction in toluene-D$_8$. The red circle marks the bridging CH_2 group.

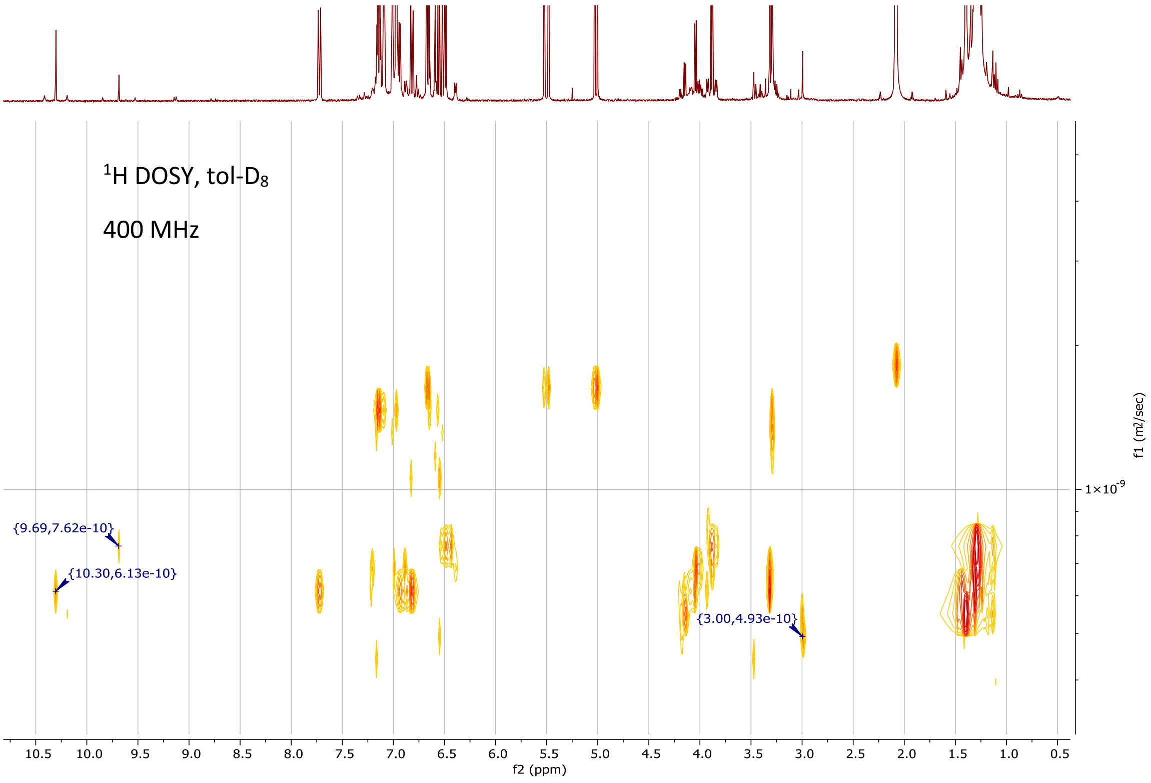

Figure S47: ^{1}H-DOSY-NMR spectrum. The spectrum shows significantly different diffusion coefficients for **4** (M =579.6 g mol^{-1}; D = 7.62 x 10^{-10} m^2 s^{-1}), **3** (M =655.71 g mol^{-1}; D = 6.13 x 10^{-10} m^2 s^{-1}), and **8** (M =1117.18 g mol^{-1}; D = 4.93 x 10^{-10} m^2 s^{-1}), respectively.

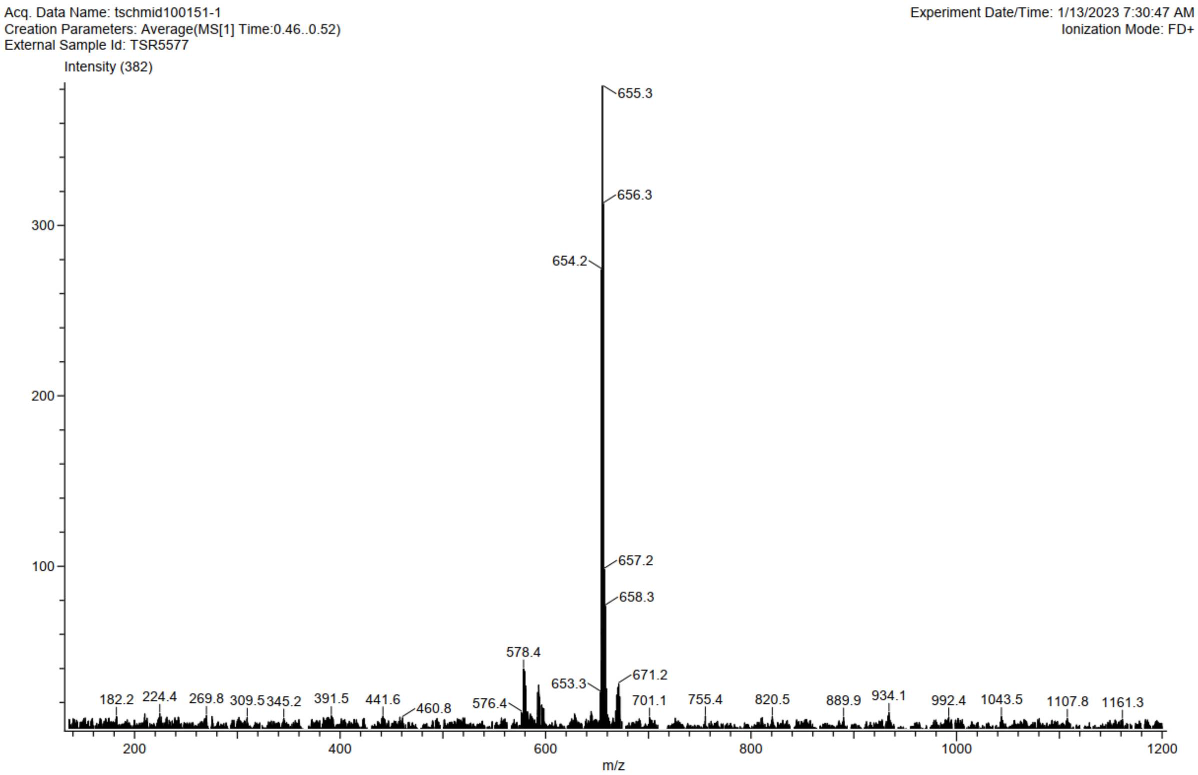

Figure S48: LIFDI-MS after dilution of the reaction mixture with THF, showing only **3** (m/z = 655.3).

1.5 Photolysis of **1** and styrene in frozen solution

1 (4.0 mg, 6.7 µmol, 1.0 eq.) and styrene (1.1 µL, 10 µmol, 1.5 eq.) are dissolved in toluene-D_8 (0.45 mL) with $OPPh_3$ as internal standard in a J-Young NMR tube. The mixture is frozen in a transparent dewar filled with liquid nitrogen and photolyzed with an LED (390 nm) for 3 h. After that, the mixture is thawed to –75°C in a cold bath and then warmed to room temperature. NMR spectra are identical with those obtained from photolysis at –75 °C.

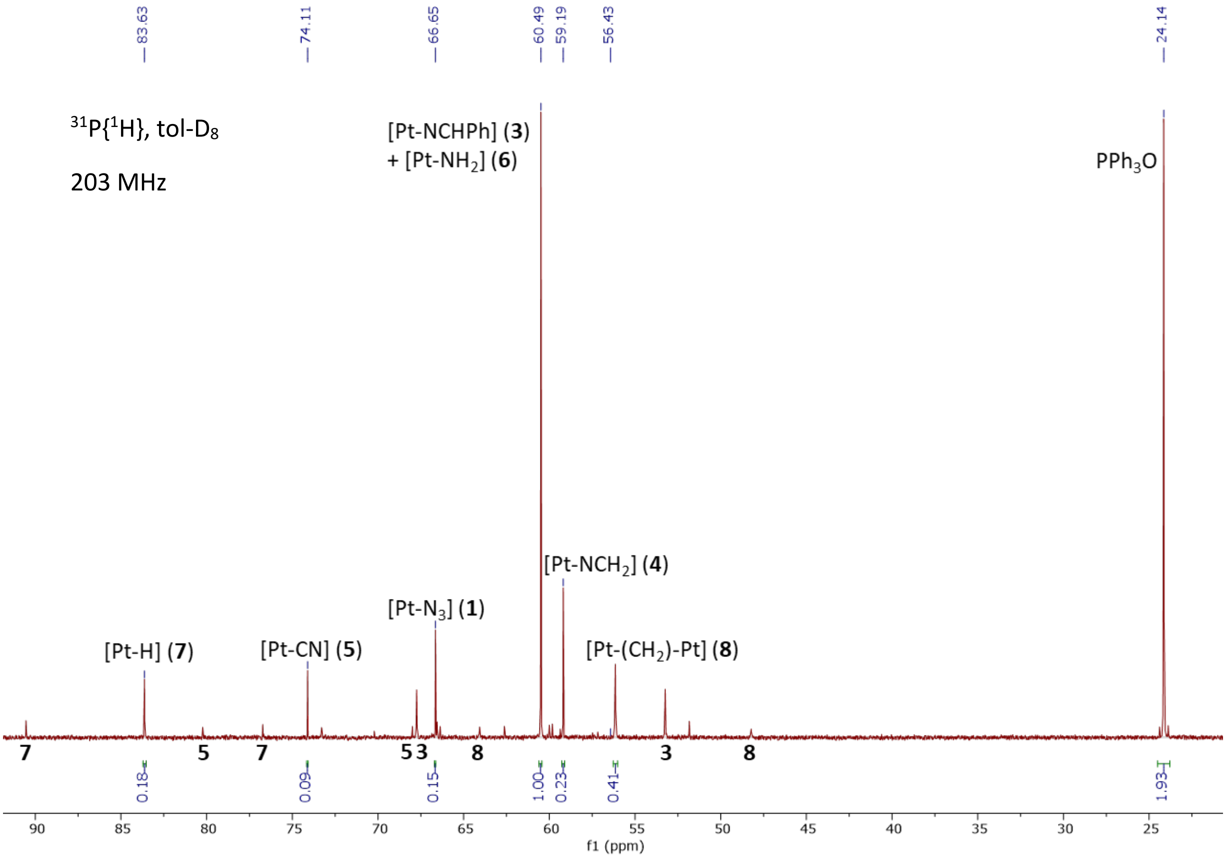

Figure S49: $^{31}P\{^1H\}$-NMR spectrum of the reaction of $Pt-N_3$ with styrene in toluene-D_8 after initial photolysis at –196 °C with $OPPh_3$ as internal standard (δ_P = 24.1 ppm).

1.6 Photolysis of **1** and *α*-methylstyrene

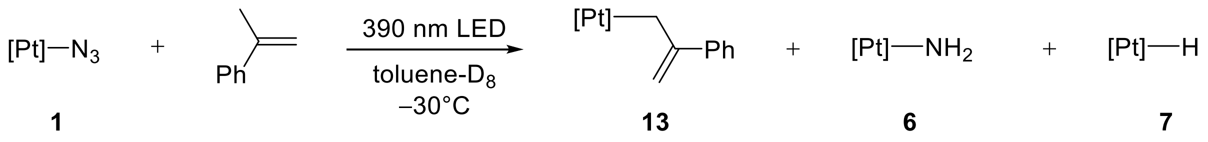

1 (6.0 mg, 10 µmol, 1.0 eq.) and *α*-methylstyrene (2.6 µL, 20 µmol, 2.0 eq.) are dissolved in toluene-d_8 (0.50 mL) in a thin Schlenk tube with 1,3,5-trimethoxybenzene as internal standard and the mixture is photolyzed (390 nm) at −30°C for 1 hour. For purification, amine **6** is protonated by addition of 2,6-lutidinium chloride (1.5 mg, 11 µmol, 1.0 eq.) in THF (0.50 mL) and stirring for 15 min. After removal of solvent *in vacuo*, the residue is extracted with pentane, filtered, and all volatiles are removed.

^{31}P{^{1}H} NMR (C_6D_6, 203 MHz, [ppm]): δ = 57.0 (s+d, $^{1}J_{CP}$ = 2900 Hz).

^{1}H{^{31}P} NMR (C_6D_6, 500 MHz, [ppm]): δ = 7.77 (AB, d, $^{3}J_{H\text{-}H}$ = 7.5 Hz, 2H, *o*-H), 7.26 (ABC, t, $^{3}J_{H\text{-}H}$ = 7.5 Hz, $^{4}J_{H\text{-}H}$ = 1.2 Hz, 2H, *m*-H), 7.17 (m, 2H, NC*H*, overlap with C_6D_6 peak, identified by ^{1}H–^{1}H COSY), 7.12 (ABC, t, $^{3}J_{H\text{-}H}$ = 7.5 Hz, $^{4}J_{H\text{-}H}$ = 1.2 Hz, 1H, *p*-H), 6.09–6.05 (m, 1H, C=C*H*H), 5.59–5.57 (m, 1H, C=CH*H*), 4.14 (AB(X), d+dd, $^{3}J_{HH}$ = 5.5 Hz, $^{3}J_{HPt}$ = 30.6 Hz, 2H, PC*H*), 3.58 (ABC(X), t+dt, $^{4}J_{H\text{-}H}$ = 1.6 Hz, $^{2}J_{HPt}$ = 89 Hz, Pt-C*H*$_2$), 1.32 (s, 36H, ^{t}Bu).

^{13}C{^{1}H} NMR (C_6D_6, 126 MHz, [ppm]): δ = 161.1(vt+dvt, $^{2}J_{CPt}$ = 55 Hz, $^{2}J_{CP}$ = 7.3 Hz, N*C*H), 156.8 (s+d, $^{2}J_{C\text{-}Pt}$ = 18.7 Hz, α–C), 146.9 (s+d, $^{3}J_{C\text{-}Pt}$ = 46 Hz, *i*–C), 128.0 (s, *m*-C), 126.4 (2 x s, *o*-C, *p*-C), 117.1 (s+d, $^{3}J_{C\text{-}Pt}$ = 68.6 Hz, C-*C*H$_2$), 83.5 (vt, $^{1}J_{CP}$ = 23.1 Hz, P*C*H), 36.1 (vt, $^{1}J_{CP}$ = 12.6 Hz, *C*(CH$_3$)$_3$), 29.4 (vt, $^{2}J_{CP}$ = 2.9 Hz, C(*C*H$_3$)$_3$), −6.1 (vt+dvt, $^{1}J_{Pt\text{-}C}$ = 668 Hz, $^{2}J_{P\text{-}C}$ = 6.5 Hz, Pt-C*H*$_2$).

^{195}Pt{^{1}H] NMR (Tol-D_8, 107 MHz, [ppm]): δ = −3806 (Pt−NH$_2$), −4418 (t, $^{1}J_{CP}$ = 2920 Hz, Pt−CH$_2$−C(CH$_2$)−Ph).

ESI-HR-MS m/z found (calc) [$C_{29}H_{49}NP_2Pt$+H]$^+$: 669.3064 (669.3064).

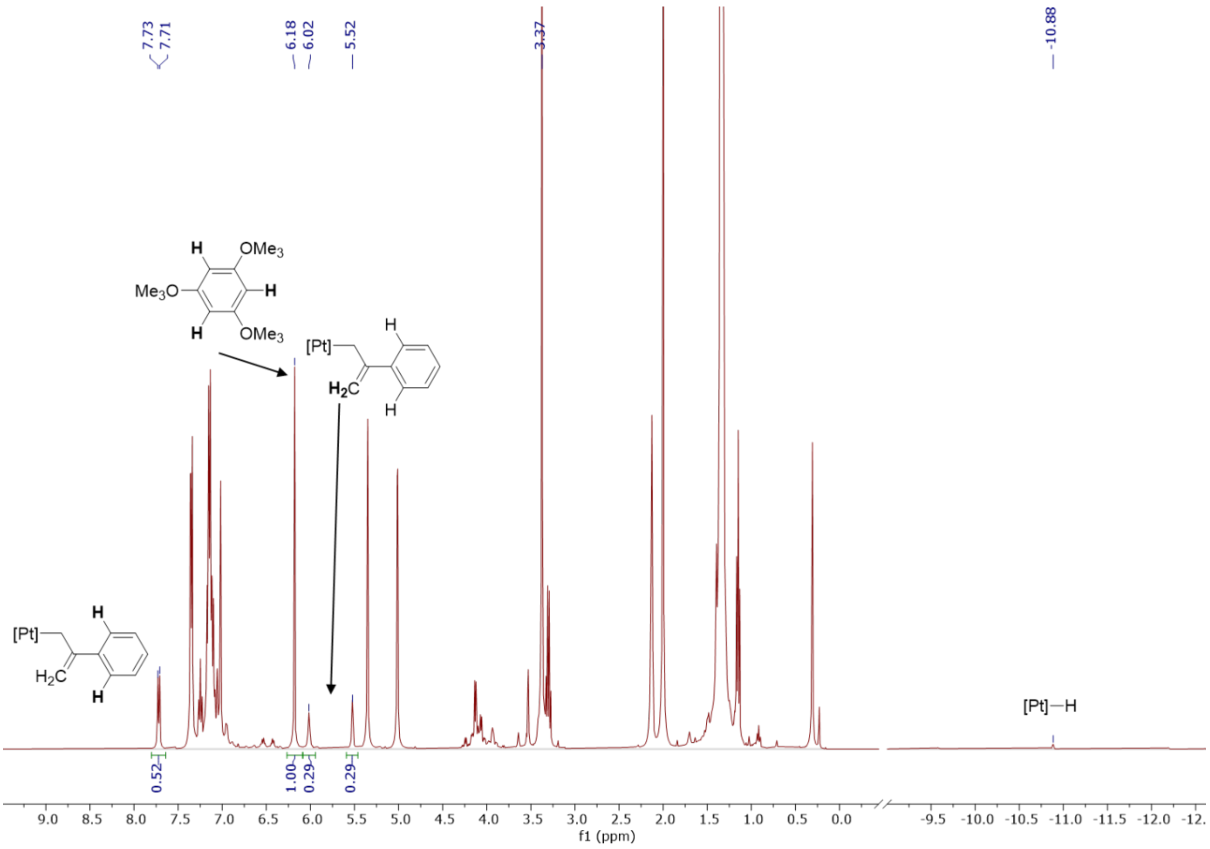

Figure S50: $^{1}H\{^{31}P\}$-NMR spectrum after photolysis of **1** and α-methylstyrene in toluene-D_8 with 1,3,5-trimethoxybenzene as internal standard.

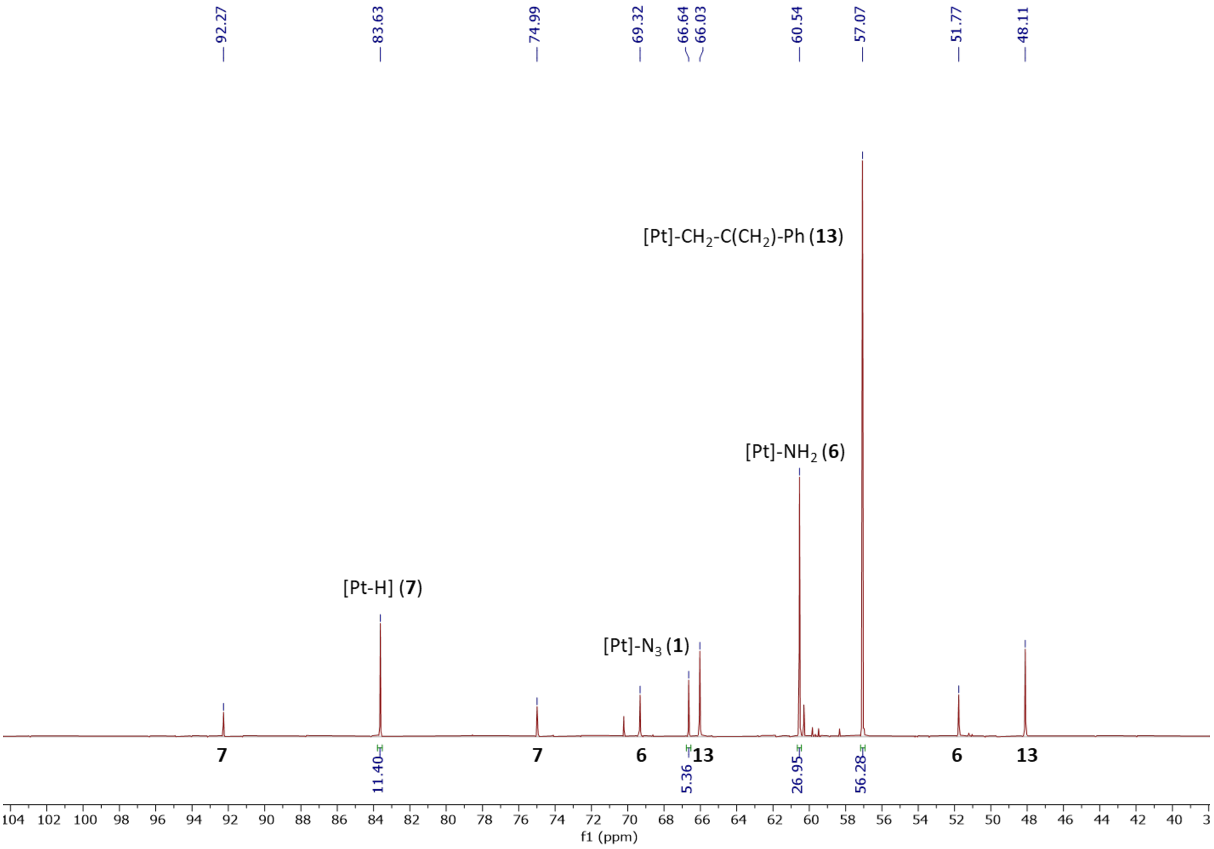

Figure S51: $^{31}P\{^{1}H\}$-NMR spectrum after photolysis of **1** and α-methylstyrene in toluene-D_8.

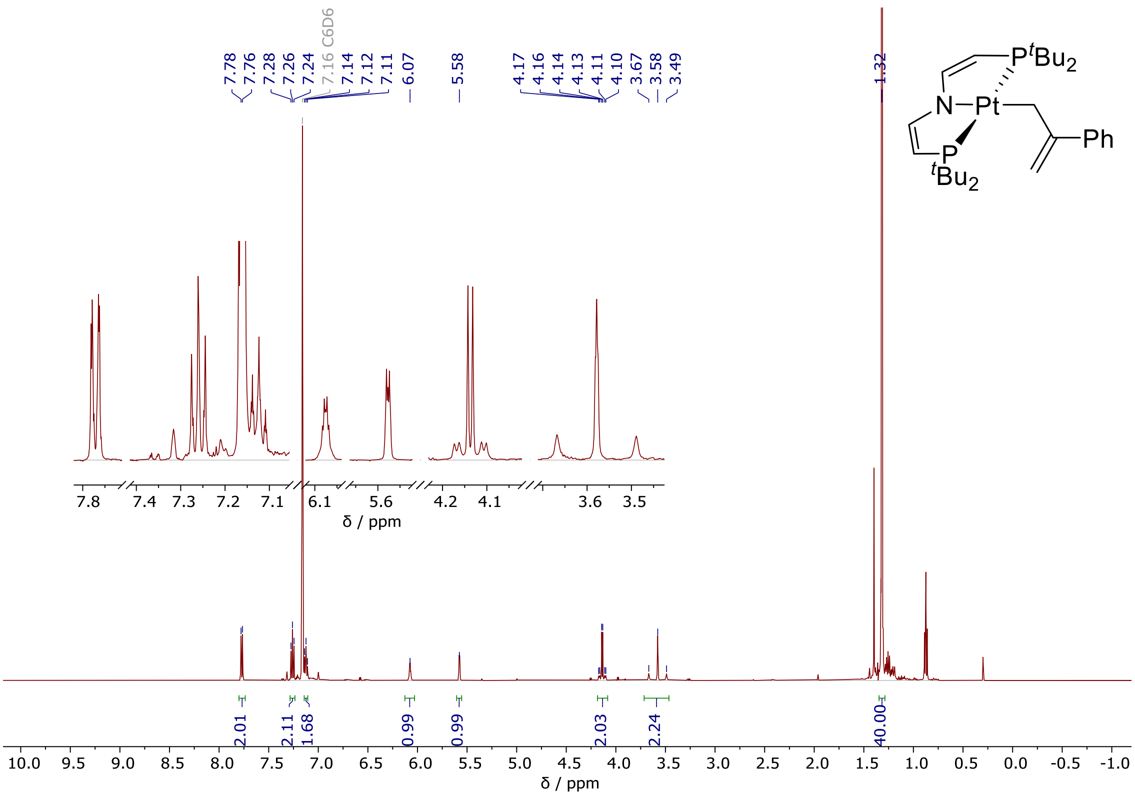

Figure S52: $^{1}H\{^{31}P\}$-NMR spectrum after purification, in C_6D_6.

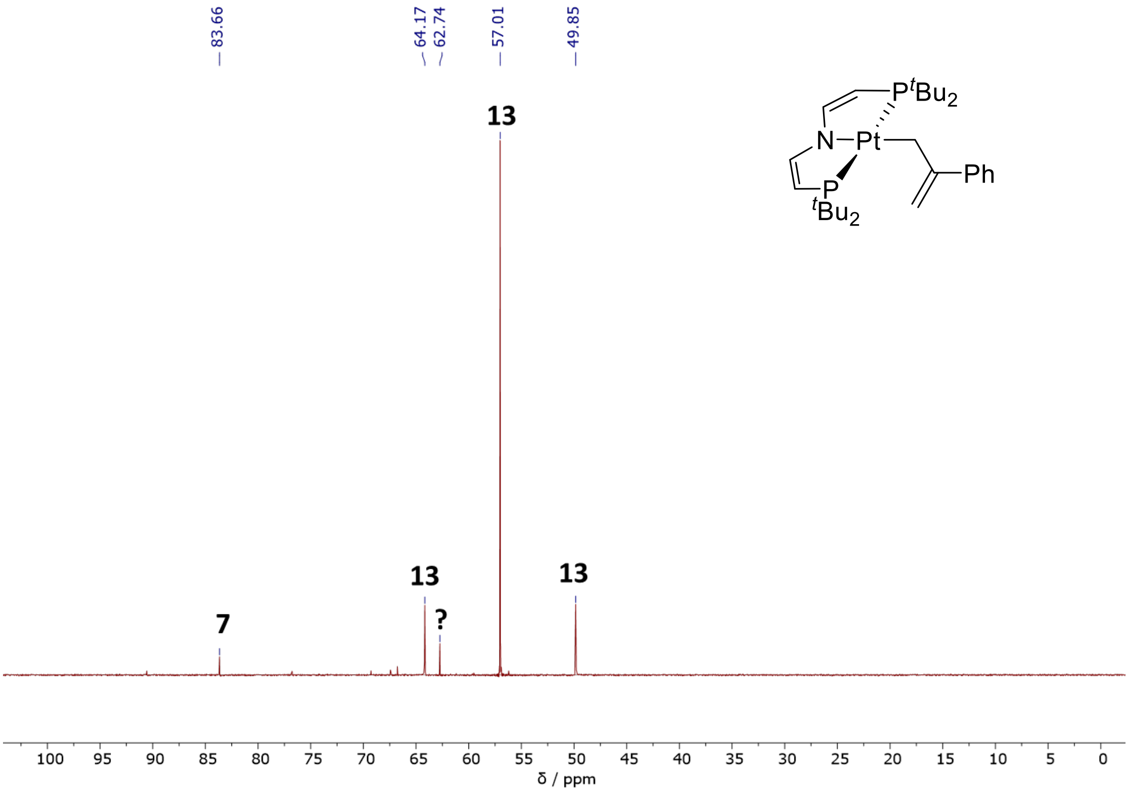

Figure S53: $^{31}P\{^{1}H\}$-NMR spectrum after purification, in C_6D_6. An additional, unknown signal is observed at 62.7 ppm.

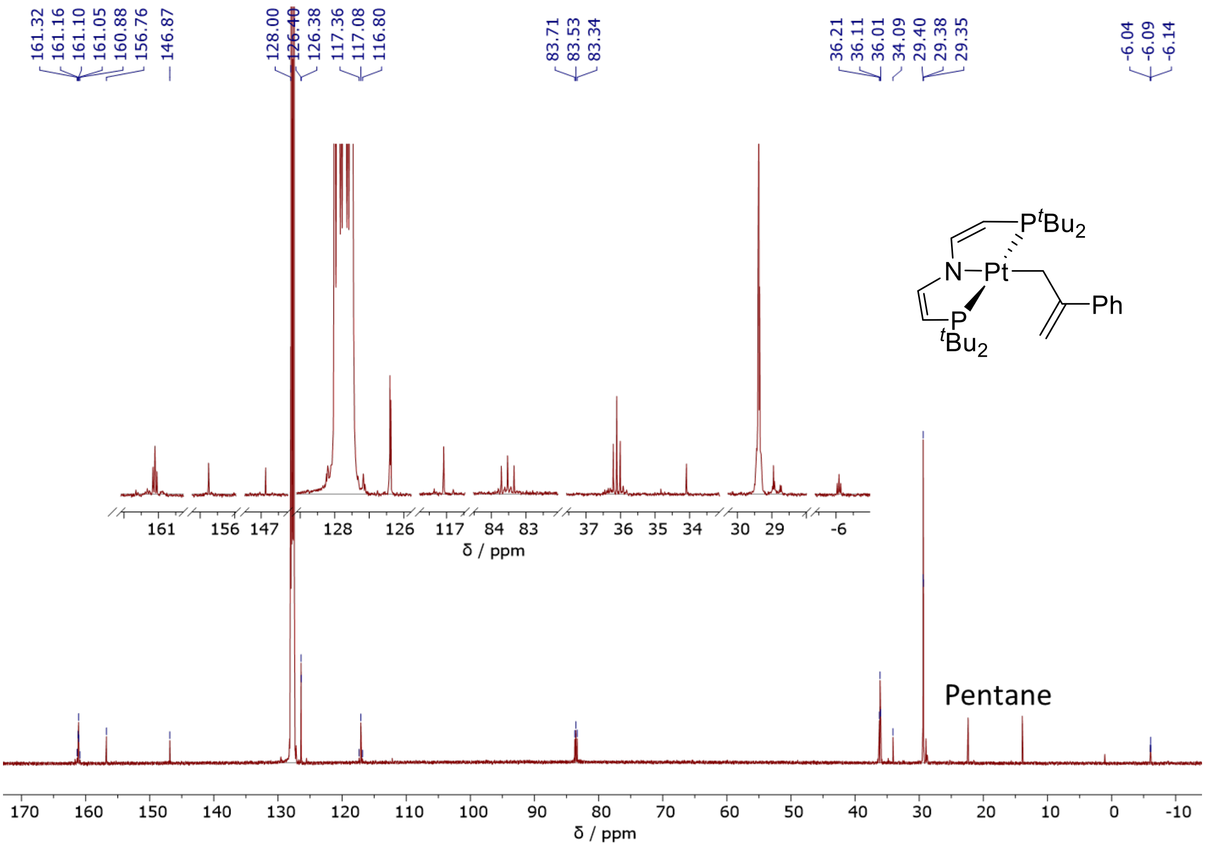

Figure S54: $^{13}C\{^1H\}$-NMR spectrum after purification, in C_6D_6.

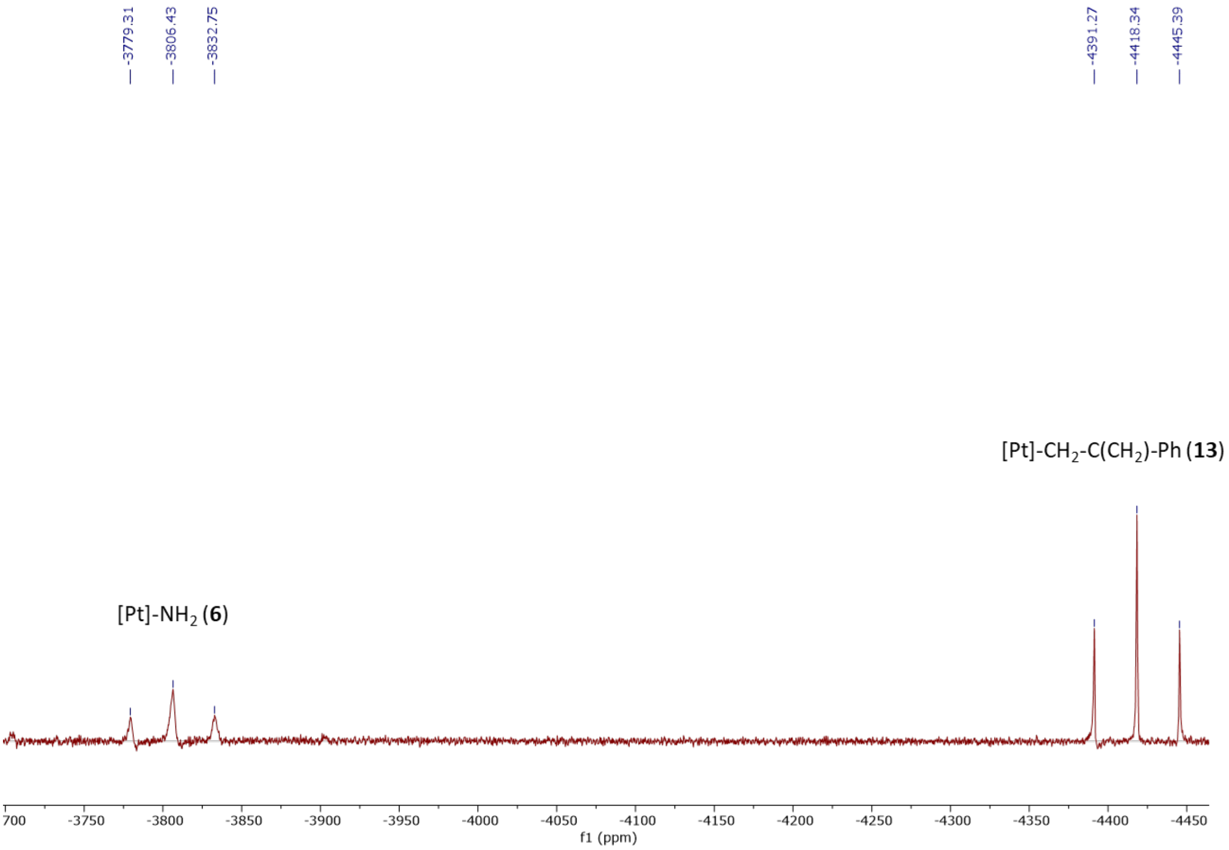

Figure S55: $^{195}Pt\{^1H\}$ NMR spectrum after photolysis of **1** and α-methylstyrene in toluene-D_8.

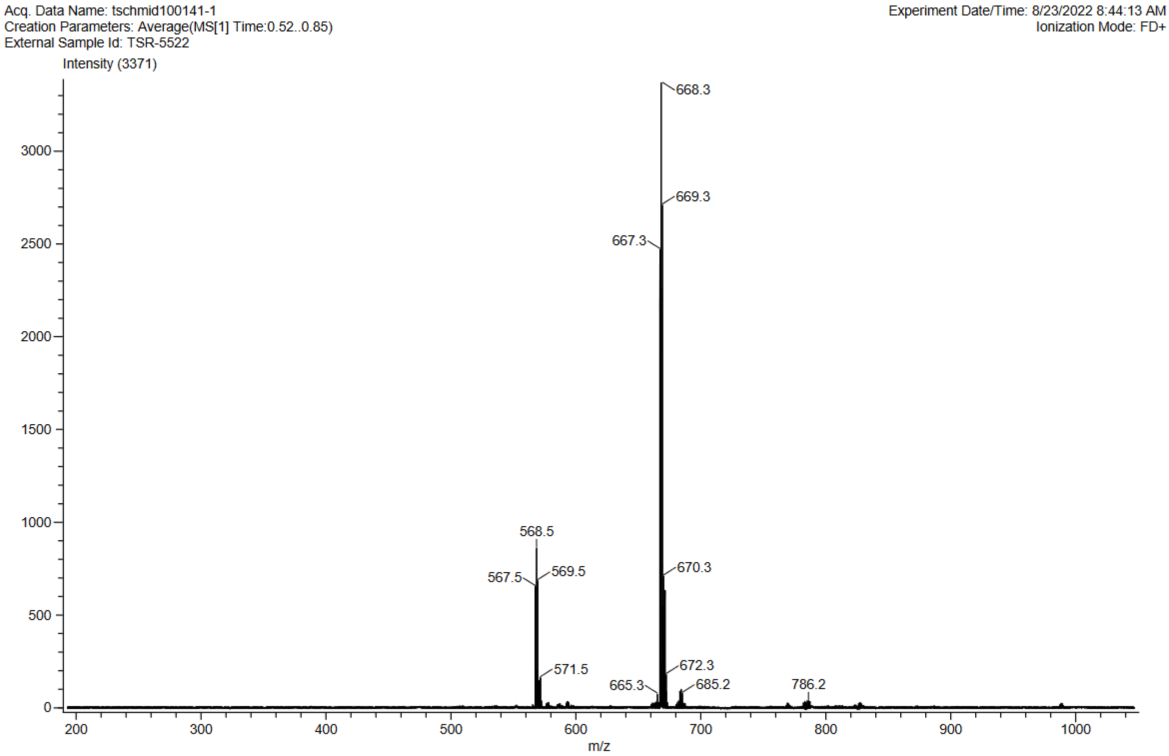

Figure S56: LIFDI-MS after dilution of the reaction mixture with THF. **13**: m/z = 668.3, **6**: m/z = 568.3.

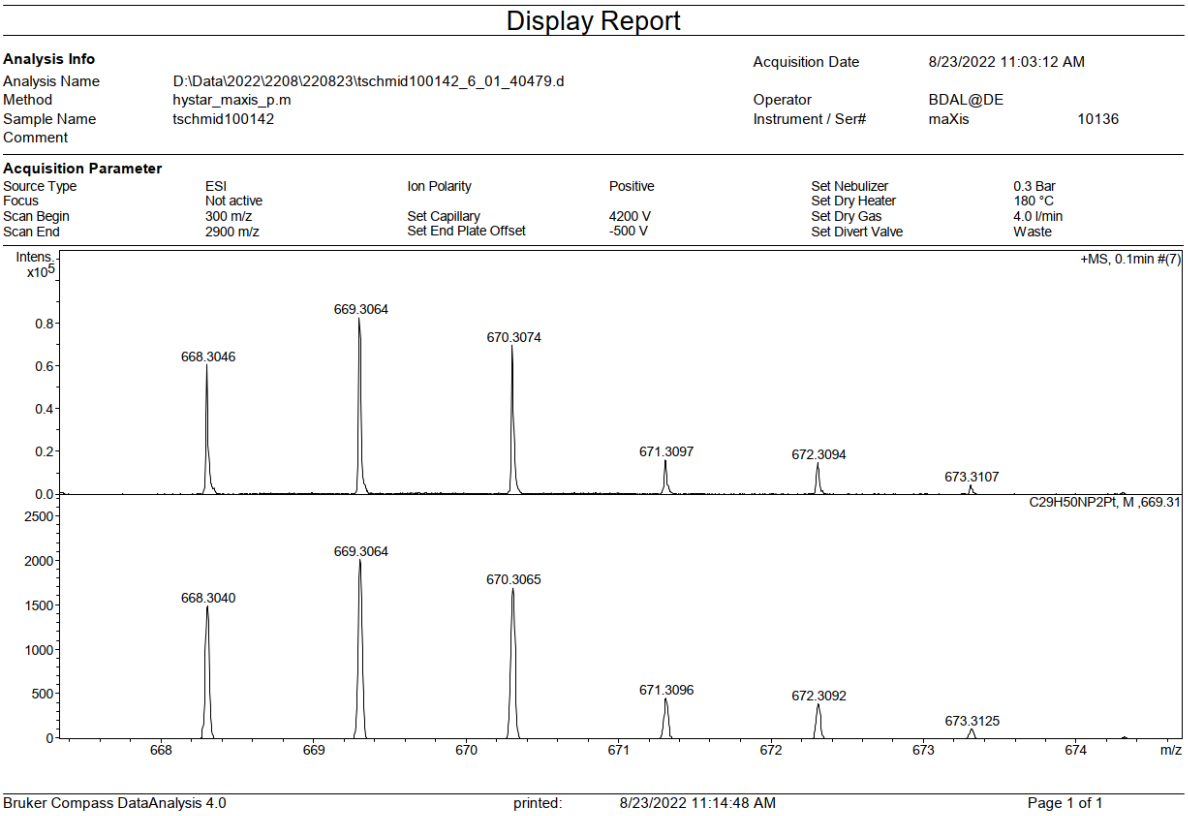

Figure S57: ESI-HR-MS after dilution of the reaction mixture with THF. Experiment (top) and simulation (bottom) for **13**.

1.6.1 Photolysis of **1** and D_3-*α*-methylstyrene

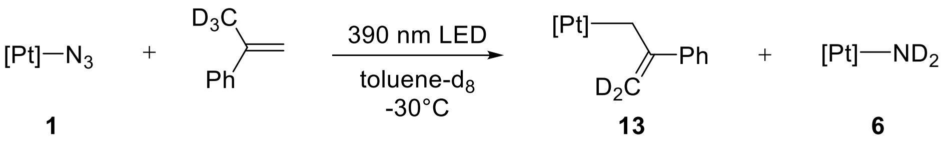

1 (4.0 mg, 6.7 µmol, 1.0 eq.) and D_3-*α*-methylstyrene (1.6 mg, 13 µmol, 2.0 eq) are dissolved in toluene-D_8 (0.45 mL) in a J-Young NMR tube and the mixture is photolyzed (390 nm) at −30°C for 15 minutes. For ^{2}H-NMR, all volatiles are removed *in vacuo* and the residue is dissolved in toluene.

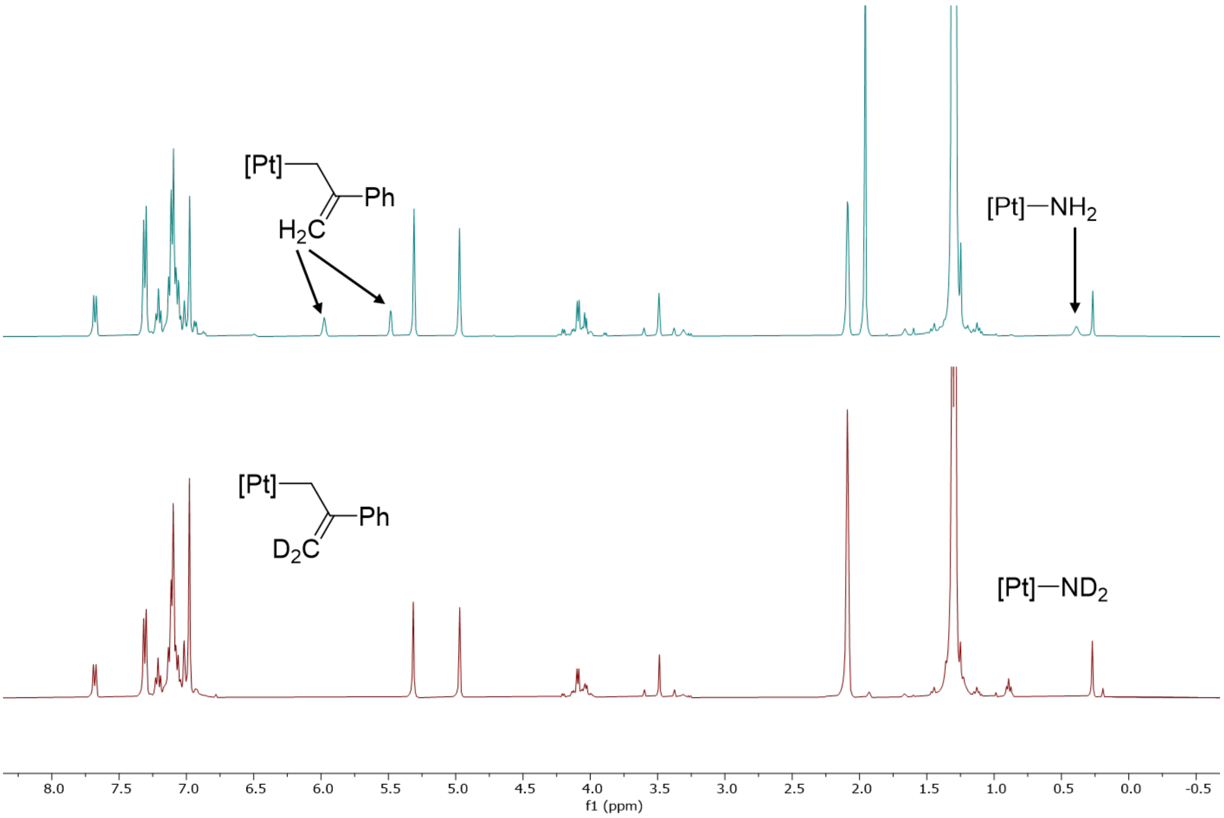

Figure S58: ^{1}H{^{31}P}-NMR spectrum (400 MHz) after photolysis of **1** and *α*-methylstyrene (top) and D_3-*α*-methylstyrene (bottom) in toluene-D_8, respectively.

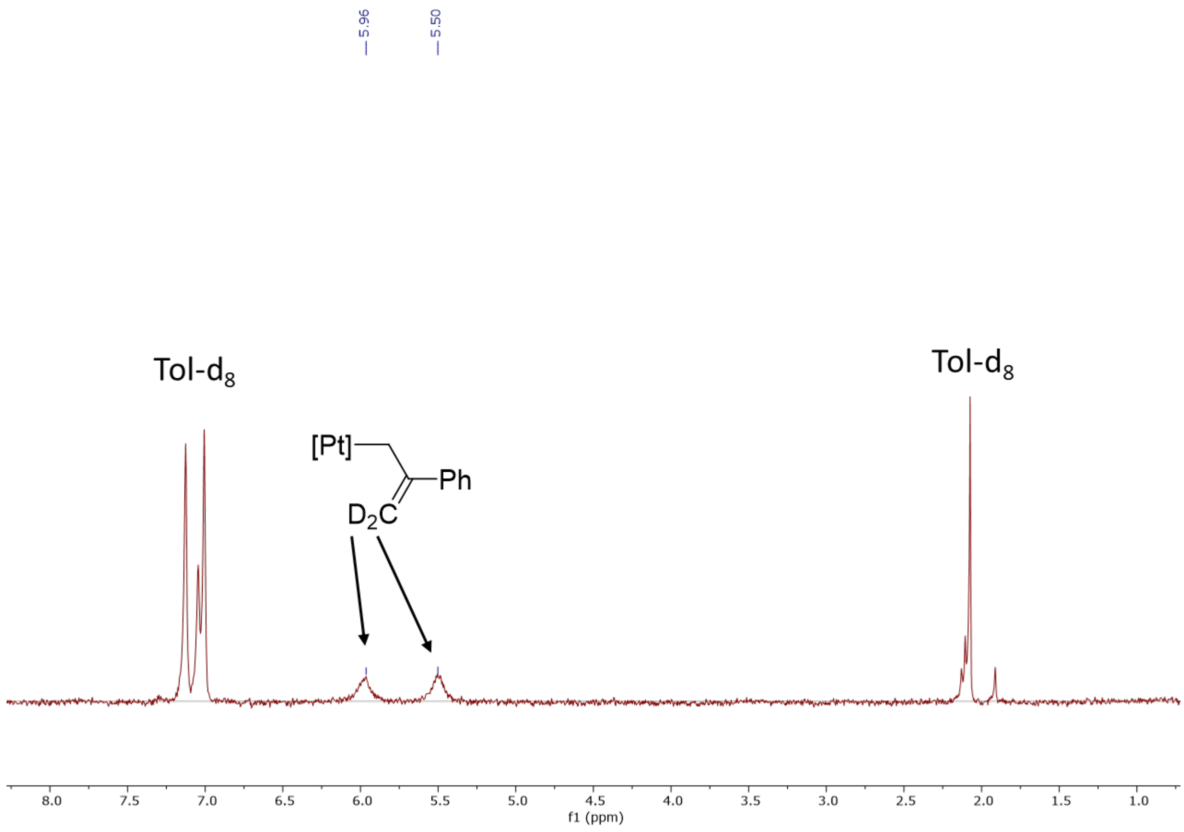

Figure S59: ^{2}H-NMR spectrum (92 MHz) after photolysis of **1** and D_3-*α*-methylstyrene in toluene.

2 Mechanistic Examinations

2.1 Light source

Different light sources were tested for the photolysis of **1** with 4-methoxystyrene. The use of monochromatic LED lamps (λ = 370 or 390 nm; FWHM = 20 nm) gave the same yields as a xenon lamp with different high-energy cut off filters (λ > 305 nm or λ > 395 nm). Reaction times significantly vary due to different photon fluxes.

Protocol: **1** (4.0 mg, 6.7 µmol, 1.0 eq.) and 4-methoxystyrene (1.5 µL, 14 µmol, 2.0 eq.) are dissolved in toluene-D8 (0.45 mL) and photolyzed in a J-Young NMR tube at −30°C.

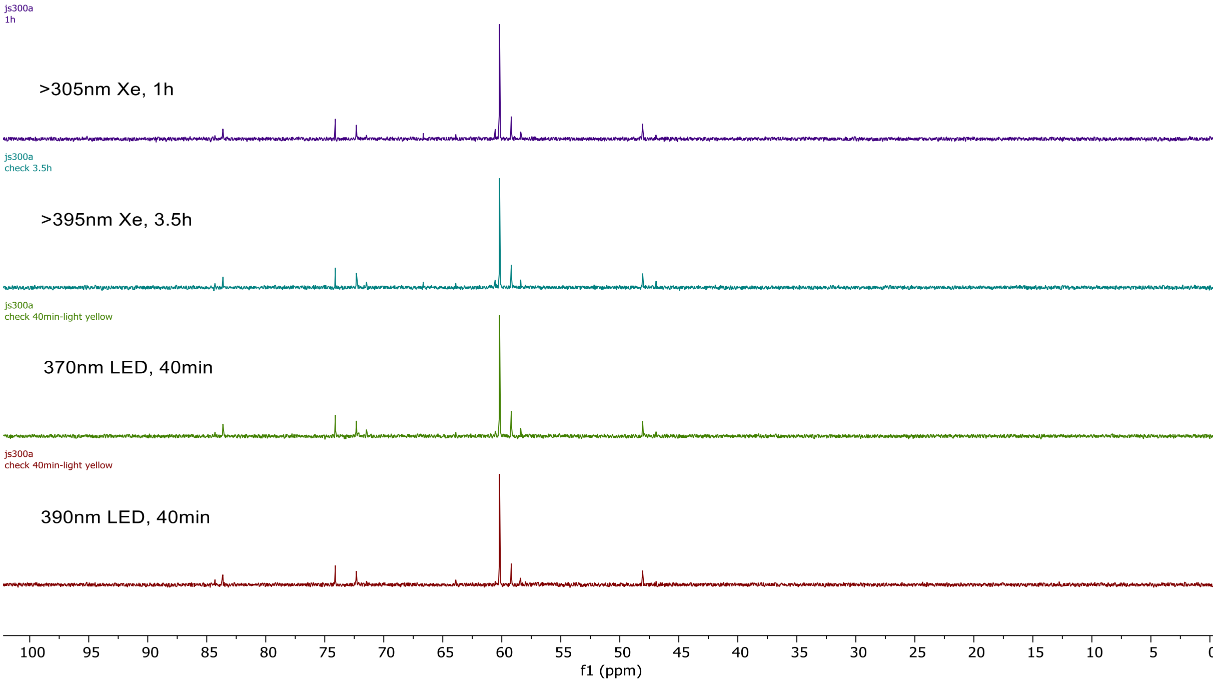

Figure S60: $^{31}P\{^{1}H\}$-NMR spectra (162 MHz) after photolysis of **1** and 4-methoxystyrene in toluene-D8 with different light sources.

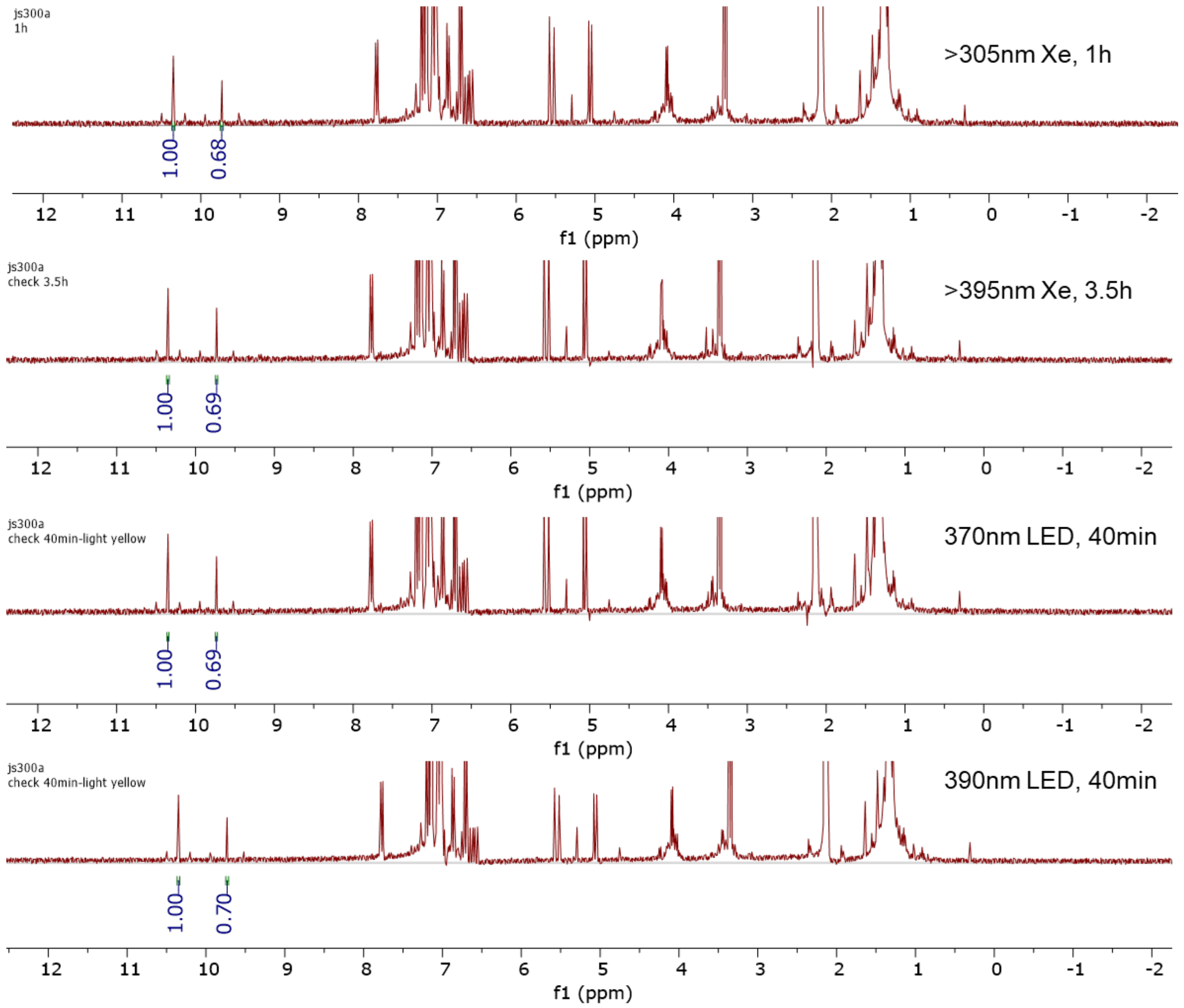

Figure S61: $^{1}H\{^{31}P\}$-NMR spectra (400 MHz) after photolysis of **1** and 4-methoxystyrene in toluene-D_8 with different light sources.

2.2 Quantum Yield

The photon flux of the 405 nm LED (I = 2.0×10^{-8} mol s^{-1}) was determined via actinometry, using the photoisomerization of 2-nitrobenzaldehyde, which has a constant quantum yield of $\phi = 0.5$ over wavelengths between 300 and 410 nm.[9]

Quantum yield determination: **1** (0.19 mg, 0.32 µM, 1.0 eq) and styrene (0.38 µL, 3.5 µmol, 11 eq) are dissolved in toluene-D_8 (0.35 mL) with 1,3,5-trimethoxybenzene as internal standard and the mixture is photolyzed at −30 °C (405 nm) inside an NMR spectrometer for 60 s, giving a conversion of 16% ($\Delta n = 0.72$ µmol). The absorbance (A_{405}) is determined by UV/Vis spectroscopy from a sample with the same concentration.

The quantum yield was calculated from the following equation:

$$\phi_{405} = \frac{\Delta n}{I \cdot t \cdot (1 - 10^{-A_{405}})} = 36 \pm 5\ \%$$

The experiment was performed twice with and without styrene, respectively, showing no significant difference in quantum yield.

2.3 Hammett Analysis

Hammett analysis was carried out by competition experiments upon photolysis of **1** in the presence of equimolar amounts of styrene and *p*-substituted styrenes. Substituent parameters (σ_p) were taken from Hansch *et al.*[10] The selectivity was derived by integration of the NC*H*Ar $^1H\{^{31}P\}$ NMR signals of the aldimido products using MestReNova's line fitting tool and cross-checked with the ^{31}P NMR intensities from an inverse gated-decoupled $^{31}P\{^1H\}$ experiment. The relative rate constants $k(X)/k(H)$ were obtained from the product ratios $c(X)/c(H)$, corrected by the ratio of the initial concentrations $c_0(X)/c_0(H)$:

$$\frac{k(\mathrm{X})}{k(\mathrm{H})} = \frac{\frac{c(\mathrm{X})}{c(\mathrm{H})}}{\frac{c_0(\mathrm{X})}{c_0(\mathrm{H})}}$$

The experimental error was estimated from the standard deviation σ_N for three runs, taking in to account the student *t*-factor for N = 3 (t = 4.303, 95% confidence interval). Exemplary NMR spectra for all substrates are shown in Figures S59-S68.

Protocol: Styrene (1.5 eq.) and the respective *para*-substituted styrene (1.5 eq.) are added to a solution of **1** (4.0 mg, 6.7 µmol, 1.0 eq) and $OPPh_3$ (0.5 eq) in toluene-D_8 (0.5 mL). The mixture is photolyzed (390 nm) at −30 °C for 15 seconds (conversion ca. 10%).

Table S1: Hammett σ_p values, mean of $\log(\frac{k(\mathrm{X})}{k(\mathrm{H})})$, and error.

Substituent (*p*-X)	σ_p	mean $\log(\frac{k(\mathrm{X})}{k(\mathrm{H})})$	$\sigma_N \cdot t$
OMe	−0.27	−0.268	0.06
^{t}Bu	−0.20	−0.161	0.015
F	0.06	0.020	0.06
Cl	0.23	0.294	0.03
CF_3	0.54	0.558	0.05

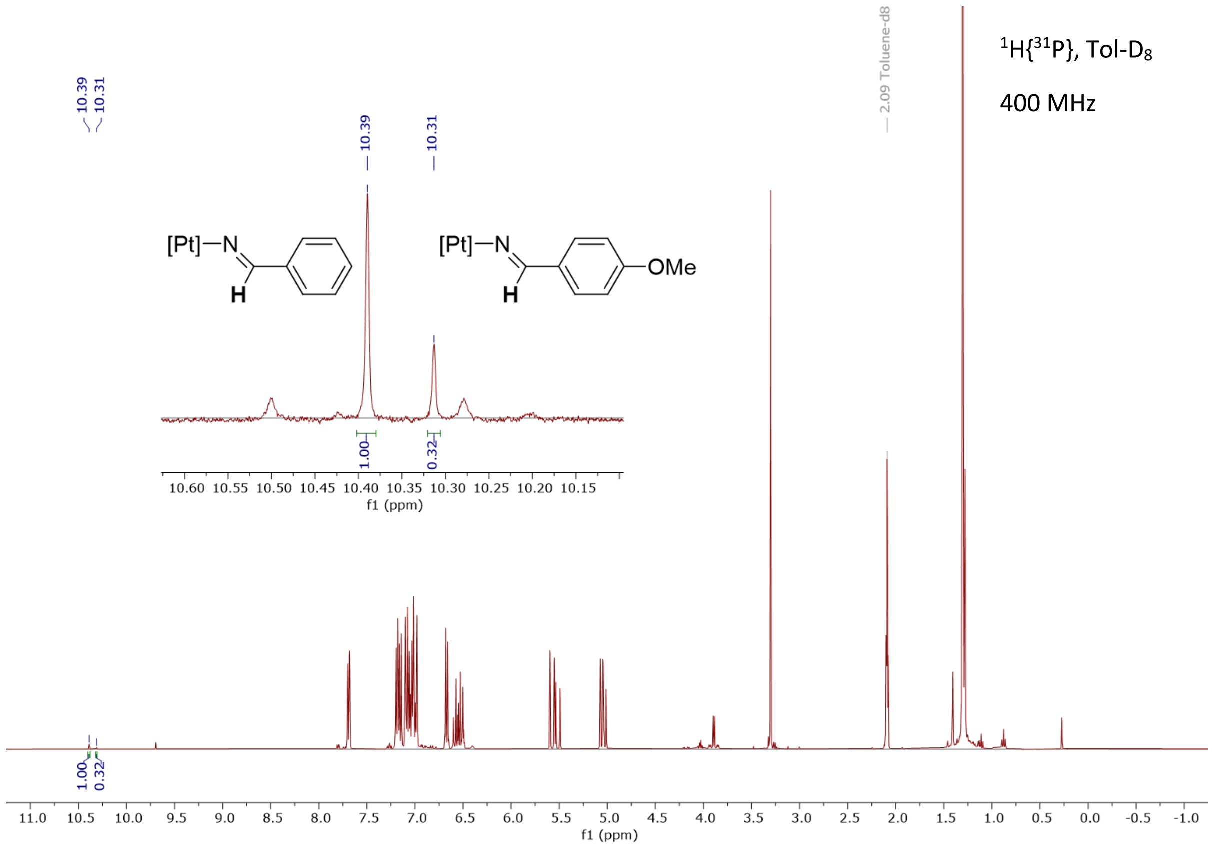

Figure S62: ^{1}H{^{31}P}-NMR spectrum after photolysis of **1** with styrene and 4-methoxystyrene.

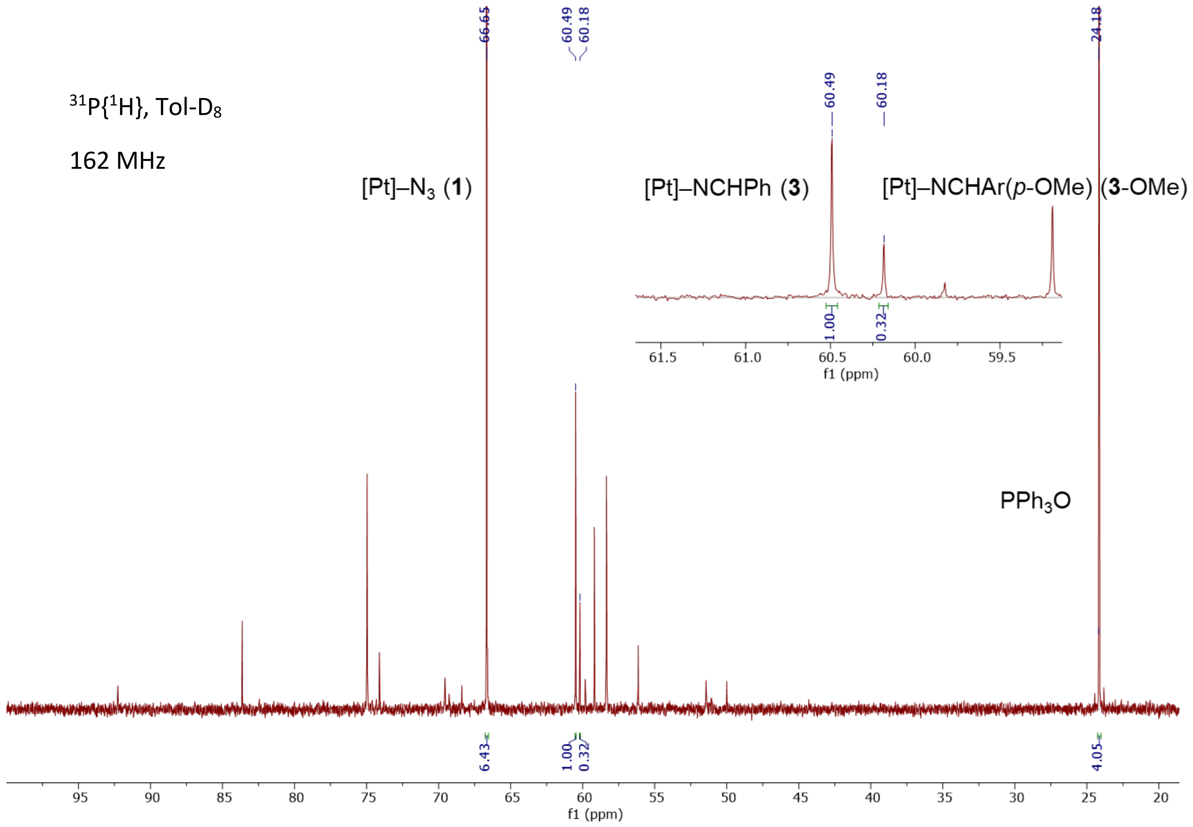

Figure S63: ^{31}P{^{1}H}-NMR spectrum after photolysis of **1** with styrene and 4-methoxystyrene with $OPPh_3$ as internal standard.

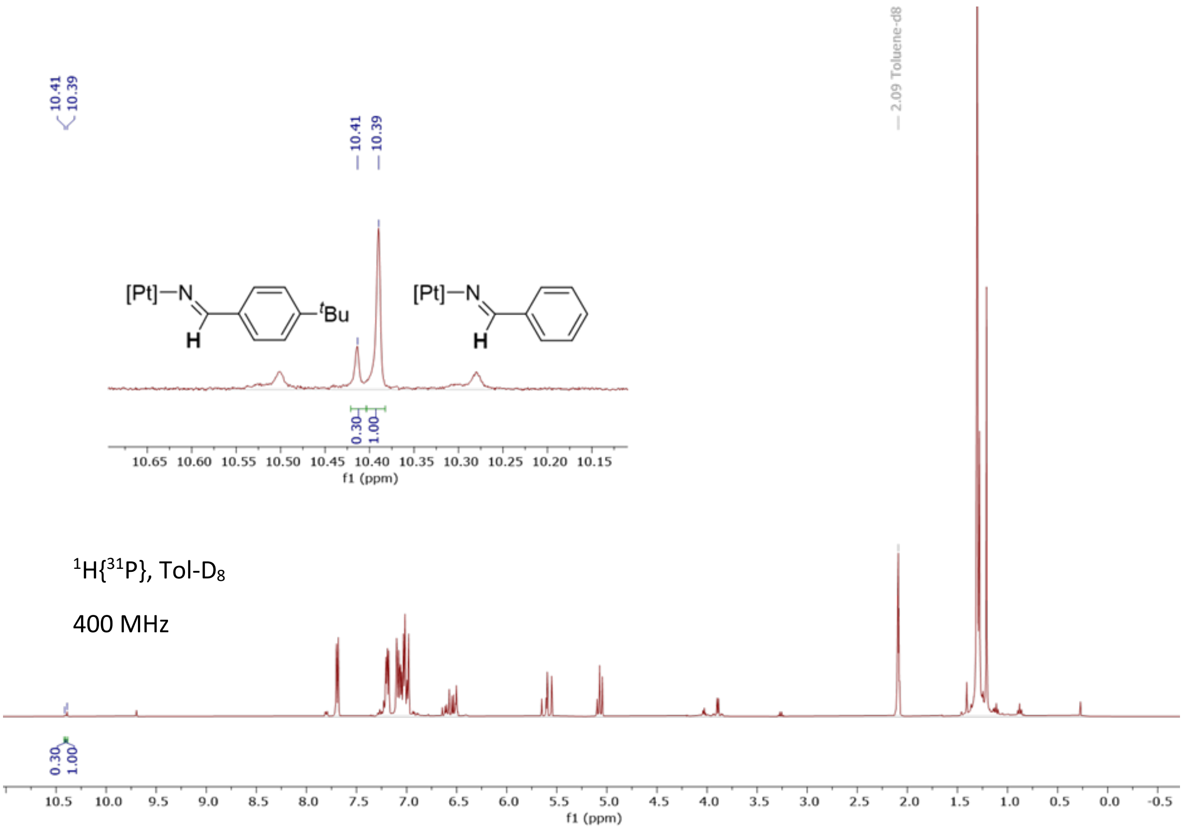

Figure S64: ^{1}H{^{31}P}-NMR spectrum after photolysis of **1** with styrene and 4-*tert*-butylstyrene.

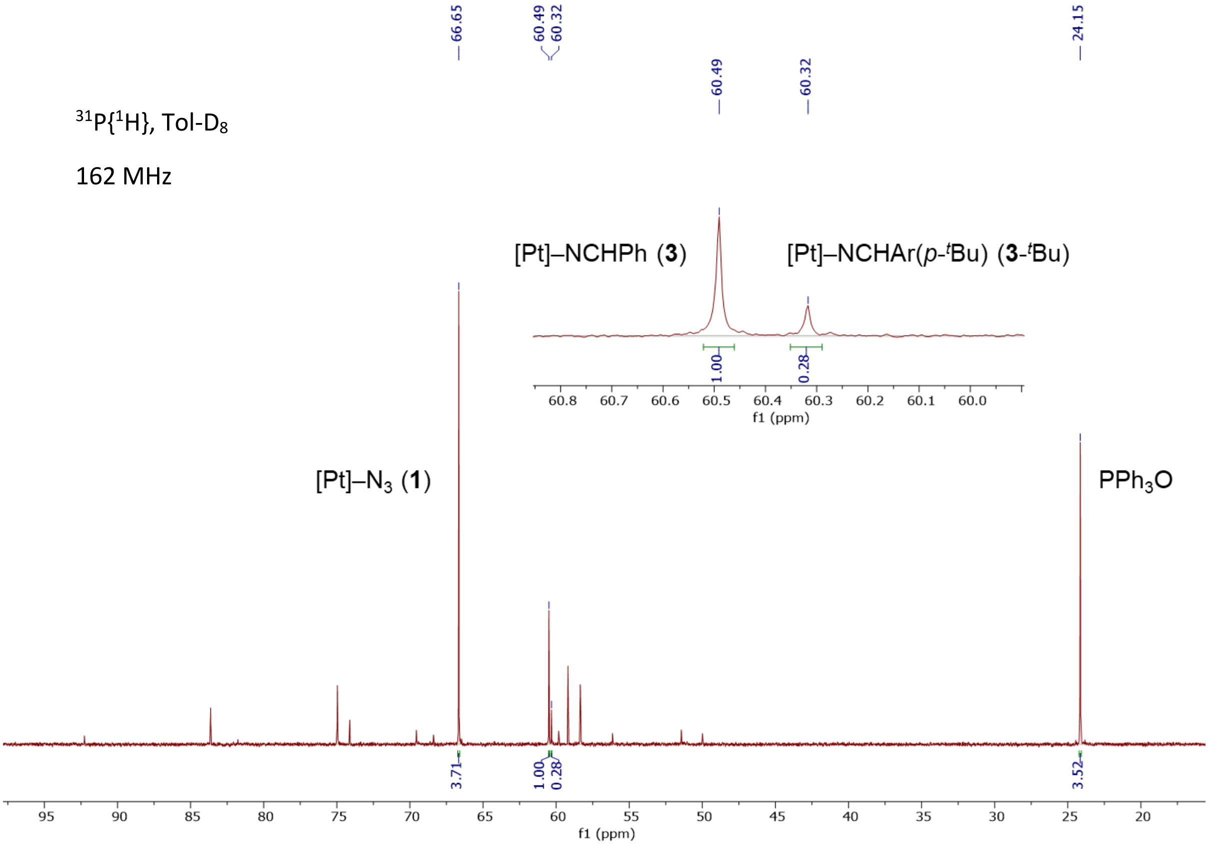

Figure S65: ^{31}P{^{1}H}-NMR spectrum after photolysis of **1** with styrene and 4-*tert*-butylstyrene with $OPPh_3$ as internal standard.

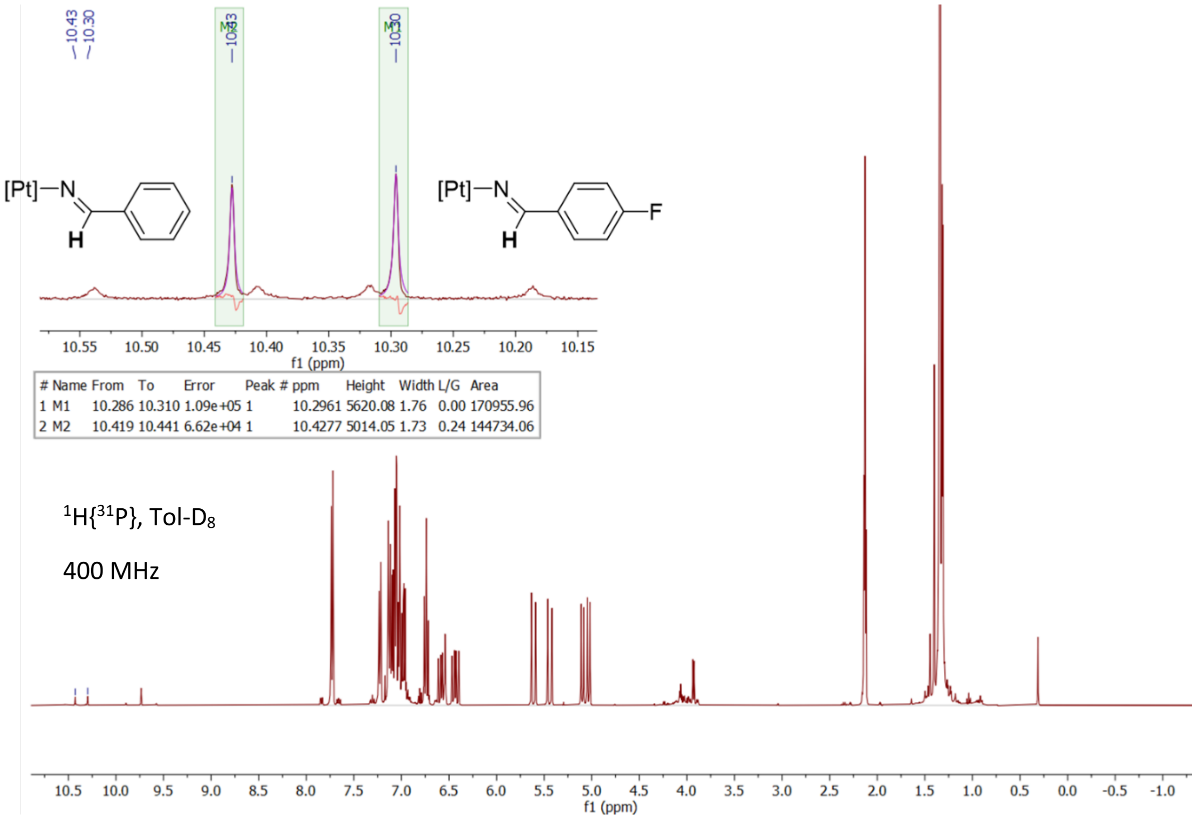

Figure S66: $^{1}H\{^{31}P\}$-NMR spectrum after photolysis of **1** with styrene and 4-fluorostyrene.

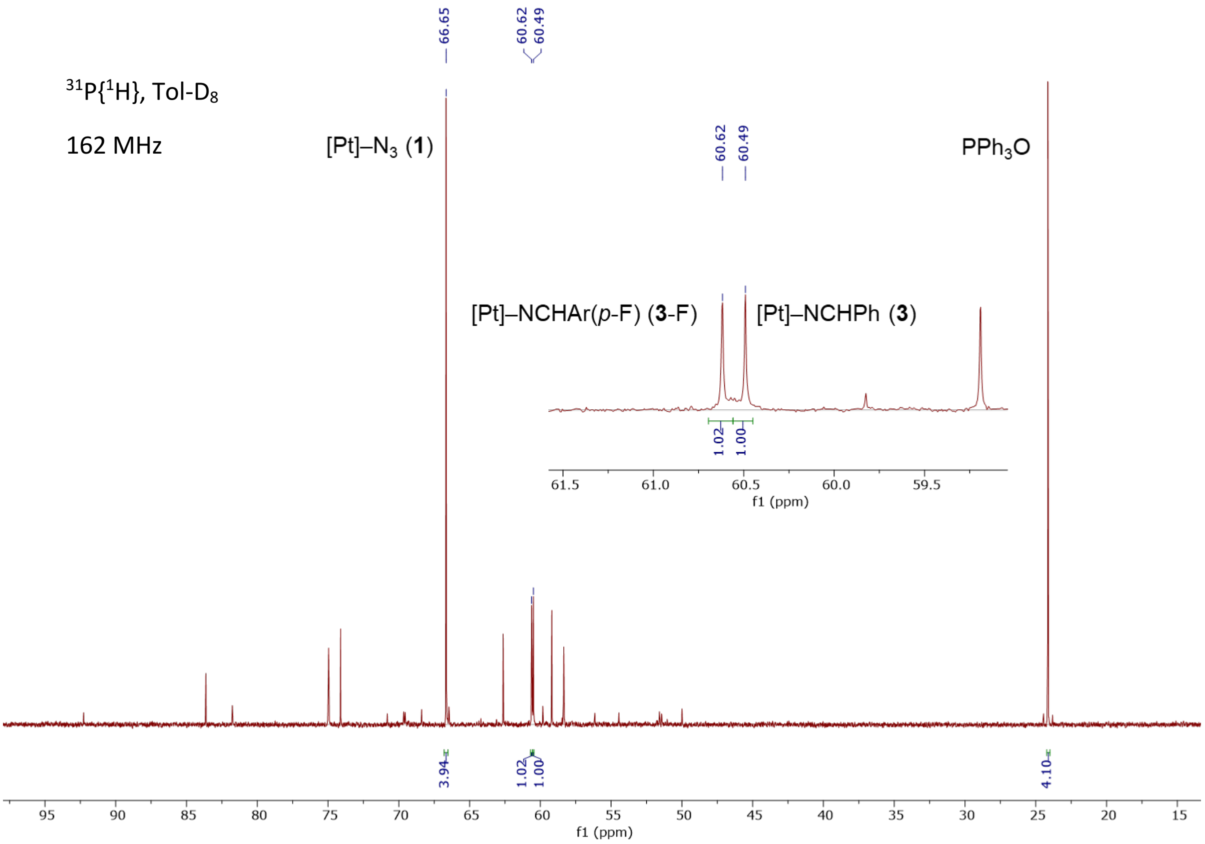

Figure S67: $^{31}P\{^{1}H\}$-NMR spectrum after photolysis of **1** with styrene and 4-fluorostyrene with $OPPh_3$ as internal standard. Due to the proximity of the signals and partial overlap, the data was not used for the Hammett analysis.

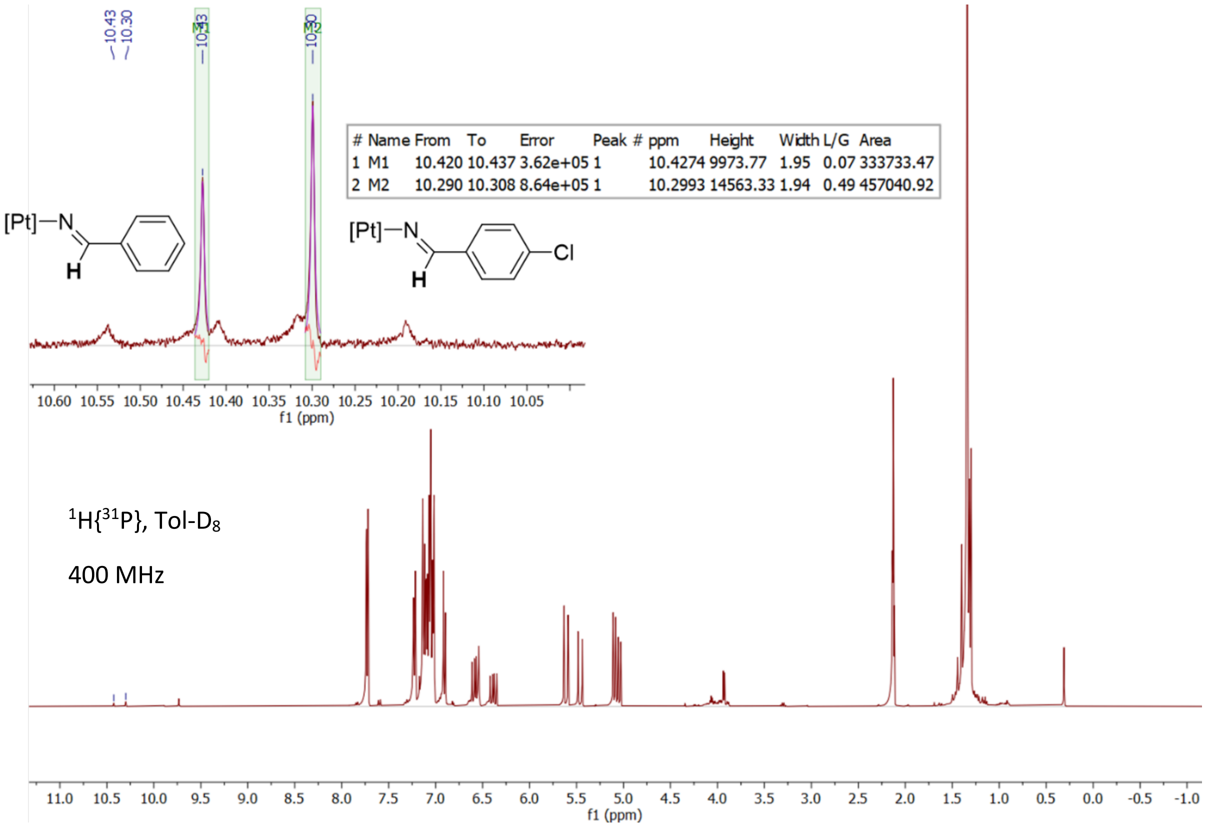

Figure S68: $^1H\{^{31}P\}$-NMR spectrum after photolysis of **1** with styrene and 4-chlorostyrene.

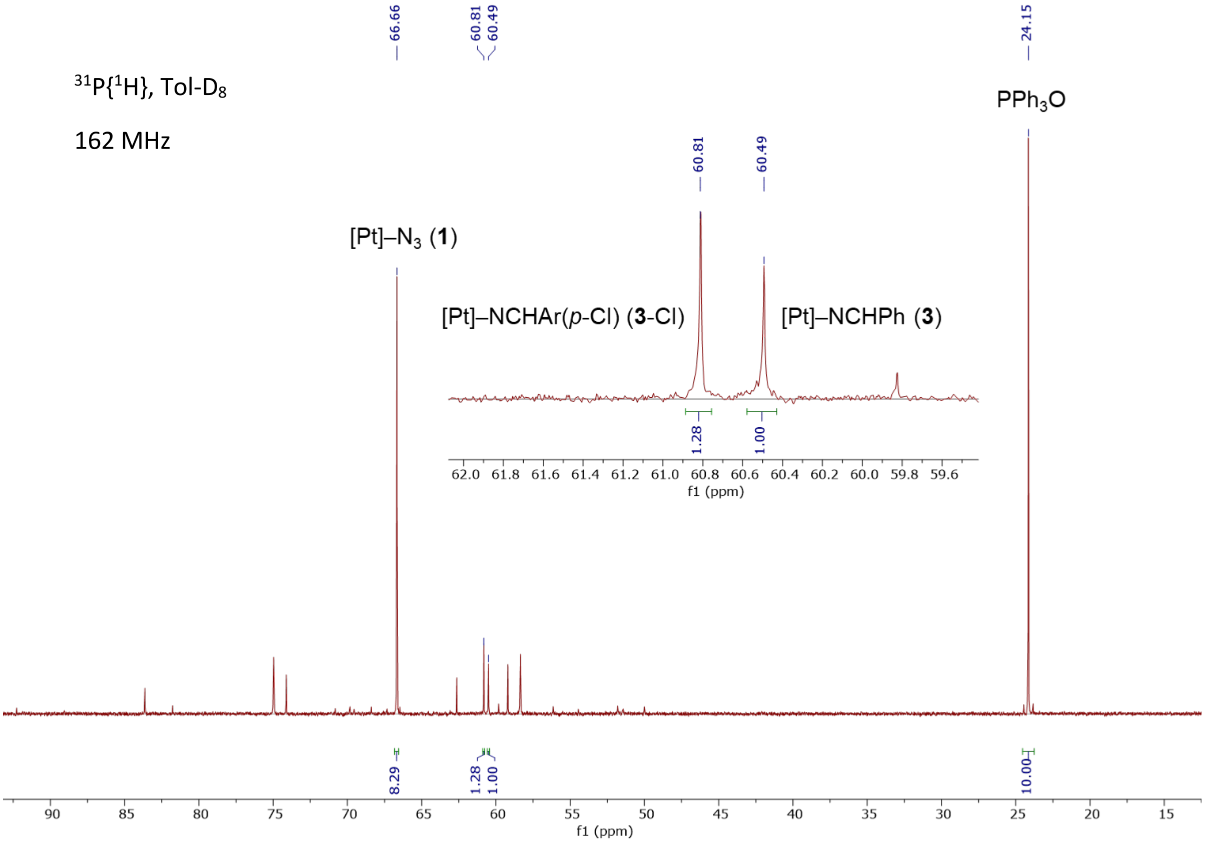

Figure S69: $^{31}P\{^1H\}$-NMR spectrum after photolysis of **1** with styrene and 4-chlorostyrene with $OPPh_3$ as internal standard.

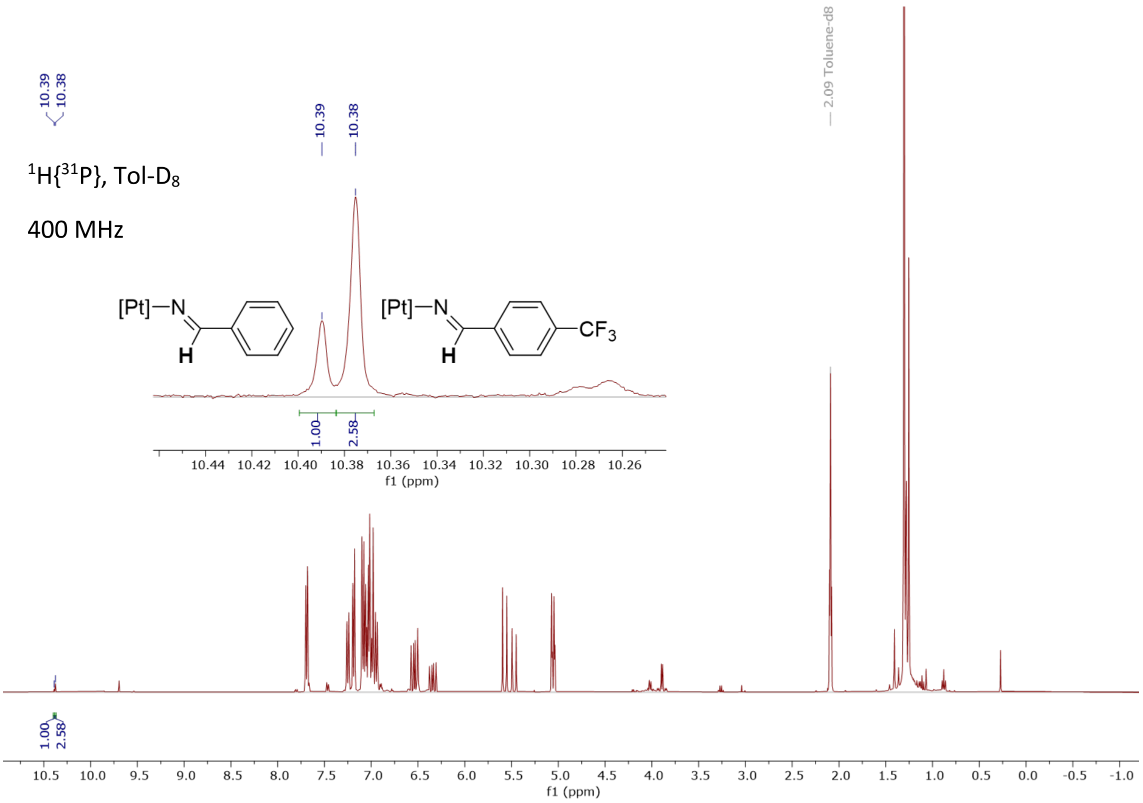

Figure S70: ^{1}H{^{31}P}-NMR spectrum after photolysis of **1** with styrene and 4-trifluoromethylstyrene.

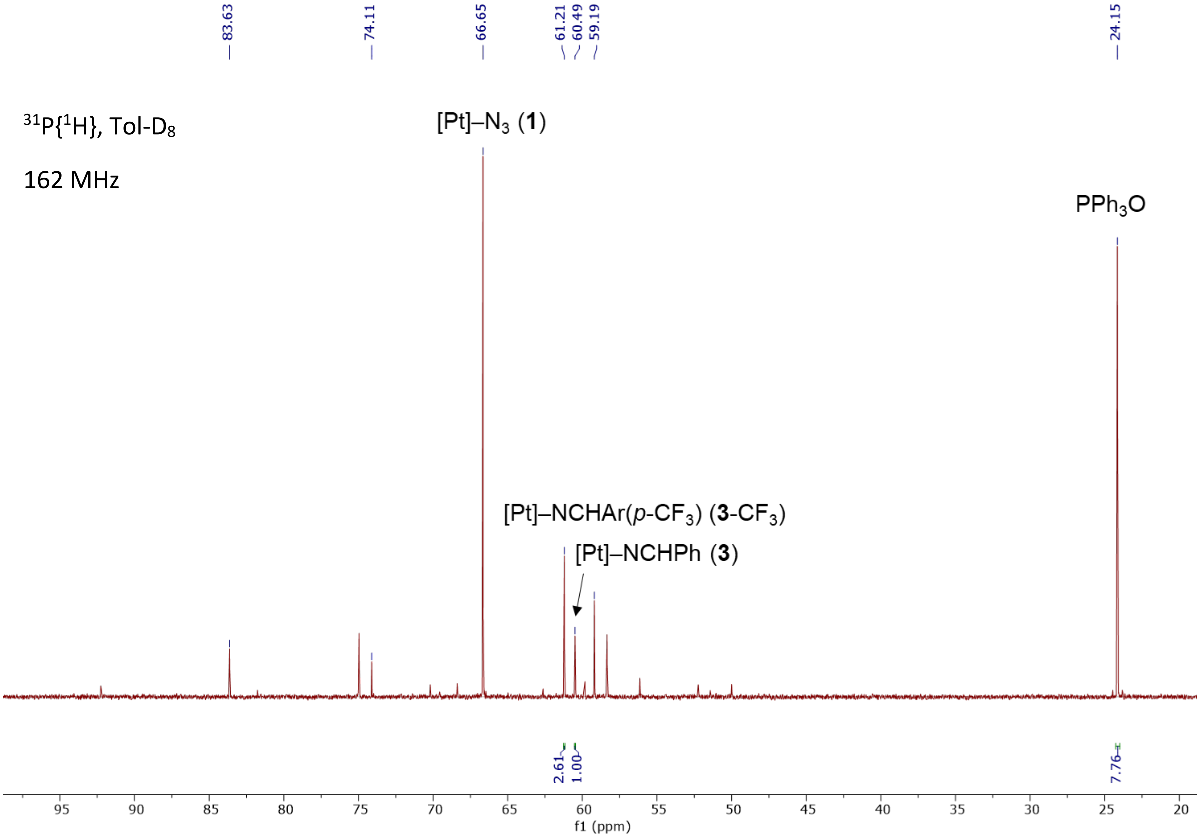

Figure S71: ^{31}P{^{1}H}-NMR spectrum after photolysis of **1** with styrene and 4-trifluoromethylstyrene with OPPh$_3$ as internal standard.

2.4 Variation of Styrene Concentration

The photolysis (390 nm, 15 min) of **1** (4.0 mg, 6.7 µmol) and varying concentrations of styrene in toluene-D_8 (0.45 mL, −30 °C) was examined. Yields vs. parent **1** were derived by inverse-gated decoupled $^{31}P\{^1H\}$-NMR spectroscopy. Experimental errors were derived from three runs using the student *t*-factor for a 95 % confidence interval.

Table S2: Spectroscopic yields vs. parent **1**.

Eq. Styrene	**3** ([Pt]–NCHPh)	**4** ([Pt]–NCH_2)	**5** ([Pt]–CN)	**6** ([Pt]–NH_2)	**7** ([Pt]–H)
0.5	(39±2)%	(15±2)%	(6±2)%	(5±2)%	(8±2)%
1	(38±2)%	(14±2)%	(5±2)%	(5±2)%	(7±2)%
10	(36±2)%	(12±2)%	(6±2)%	(6±2)%	(8±2)%
100	(38±2)%	(10±2)%	(5±2)%	(5±2)%	(6±2)%

2.5 Irradiation at room temperature (25 °C)

Photolysis (390 nm, 15 min) of **1** (4.0 mg, 6.7 µmol) and styrene in toluene-D_8 (0.4 mL, 25 °C) gave the following yields:

Table S3: Spectroscopic product yield vs. parent **1**.

Compounds	**3**	**4**	**5**	**6**	**7**
Yields	35%	6%	8%	5%	10%

2.6 Selective deuteration of styrene

Hydrogen transfer was probed by use of selectively deuterated styrene isotopologues as a substrate. After irradiation, full retention of the deuteration was observed in the products. At low temperature (−75 °C), full deuteration of the bridging methylene group of **8** is observed with β-D_2-labelled styrene.

Protocol: **1** (4.0 mg, 6.7 µmol, 1.0 eq.) and α-D_1- or β-D_2-labelled styrene (1.1 µL, 10 µmol, 1.5 eq.), respectively, are dissolved in toluene-D_8 (0.45 mL) with 1,3,5-trimethoxybenzene as internal standard in a J-Young NMR. The mixture is photolyzed at −30 °C or −75 °C (390 nm) for 15 minutes and examined by NMR spectroscopy.

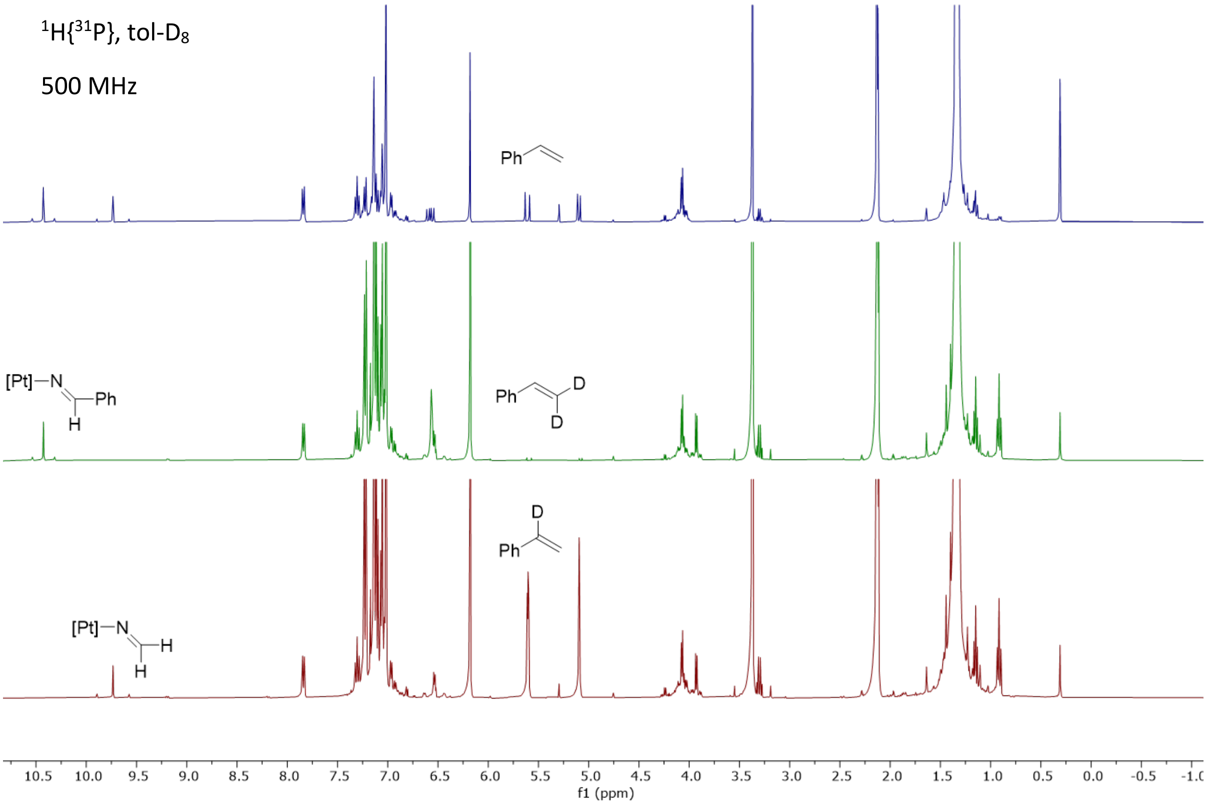

Figure S72: $^1H\{^{31}P\}$-NMR spectrum after irradiation of **1** and selectively deuterated styrenes at –30°C with 1,3,5-trimethoxybenzene as internal standard.

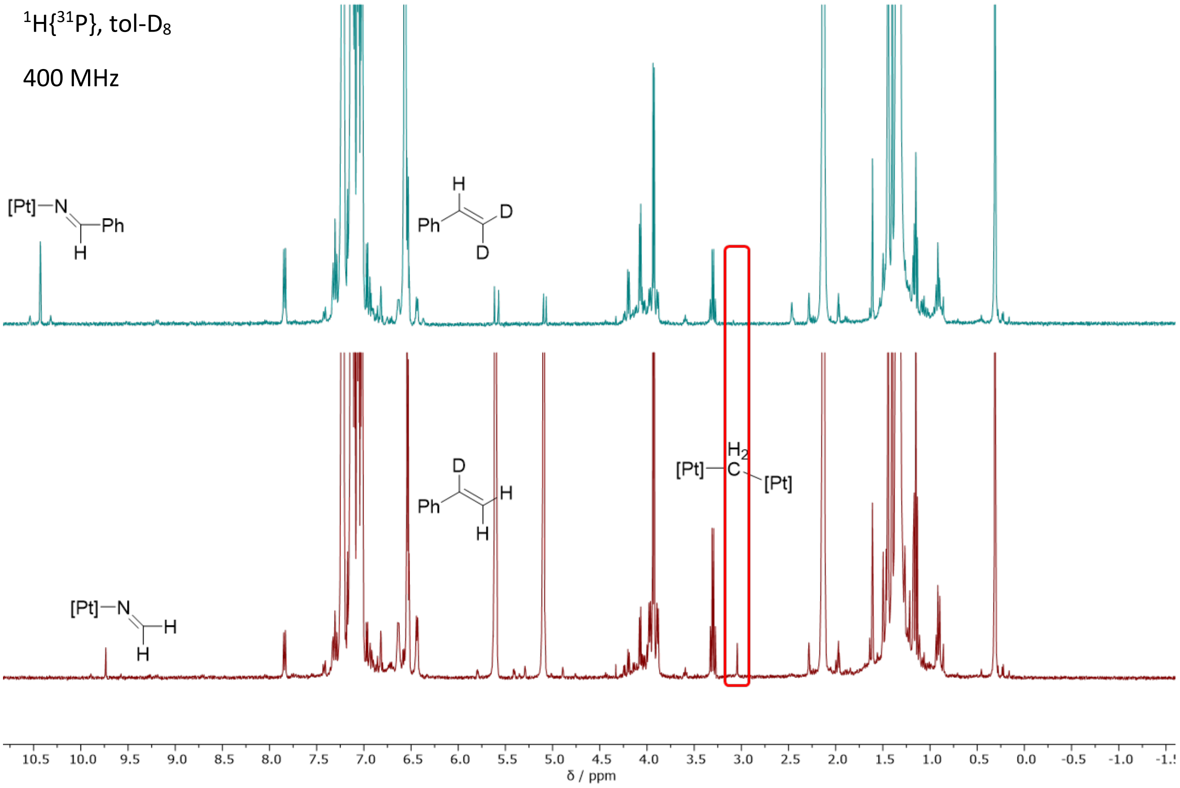

Figure S73: $^1H\{^{31}P\}$-NMR spectrum after irradiation of **1** and selectively deuterated styrenes at –75°C.

2.7 Kinetic Isotope Effect

Protocol: Styrene (2 eq.) and styrene-d_8 (2 eq.) are added to a solution of **1** (4.0 mg, 6.7 µmol, 1.0 eq) in toluene-D_8 (0.5 mL). The mixture is photolyzed (390 nm) at −30 °C for 1 minute.

The ratios of deuterated vs. hydrogenated aldimido (**3**) and formimido (**4**) products (**Table S4**) were obtained from integration of the respective signals in the inverse gated-decoupled $^{31}P\{^{1}H\}$-NMR spectrum, as well as intensities of **3** and **3**-D from ESI-MS, assuming equal ionization cross sections of the isotopologues. The slightly higher KIE obtained for **4** might be caused by the decay reaction of **4**, i.e., hydrogen atom transfer to form cyanide **6** and amide **7**. The KIE obtained from **3** is therefore considered as more reliable.

Table S4: Ratios of **3**/**3**-D and **4**/**4**-D obtained from competition experiments with styrene and styrene- D_8.

Method	**3**/**3**-D	**4**/**4**-D
NMR	1.14±0.02	1.3±0.2
ESI-MS	1.17±0.02	–

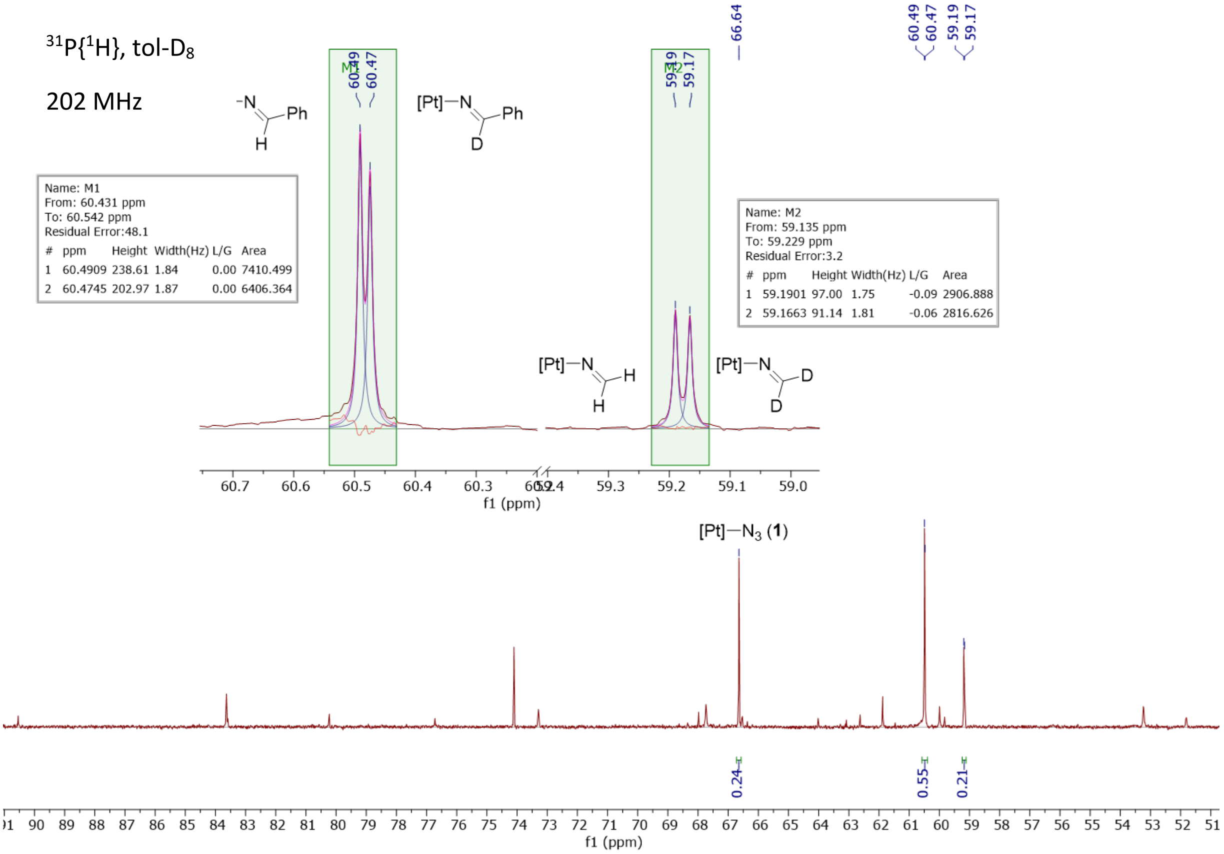

Figure S74: $^{31}P\{^{1}H\}$-NMR spectrum after photolysis of **1** and styrene/styrene-D_8.

Display Report

Analysis Info

Analysis Name	Z:\Data\2022\2207\sam270722\tschmid100138_18_01_117821.d	Acquisition Date	28.07.2022 00:56:57
Method	hystar_pl.m	Operator	BDAL@DE
Sample Name	tschmid100138	Instrument / Ser#	micrOTOF 10237
Comment			

Acquisition Parameter

Source Type	ESI	Ion Polarity	Positive	Set Nebulizer	1.2 Bar
Focus	Not active			Set Dry Heater	180 °C
Scan Begin	50 m/z	Set Capillary	4500 V	Set Dry Gas	4.0 l/min
Scan End	1600 m/z	Set End Plate Offset	-500 V	Set Divert Valve	Source

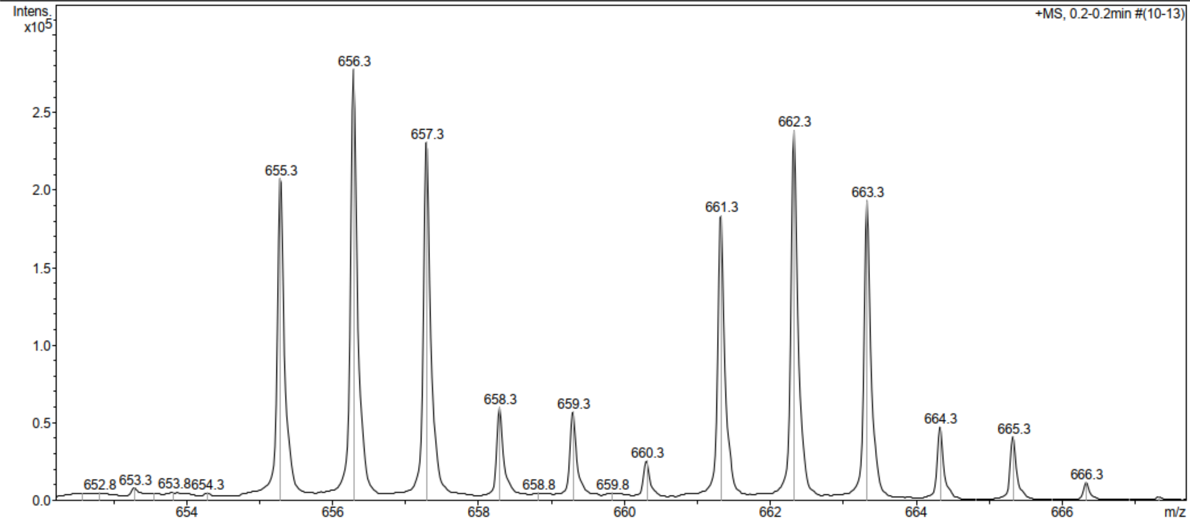

Bruker Compass DataAnalysis 4.0 printed: 27.07.2022 14:00:53 Page 1 of 1

Figure S75: ESI-MS after dilution of the reaction mixture with THF. **3**+H+: m/z = 656.3.

2.8 Photolysis of 1 at low temperatures

2.8.1 UV/Vis spectroscopy

1 is dissolved in 2-MeTHF (1.3 mM) in a reinforced quartz cuvette and sealed with a rubber septum. After freezing the cuvette in liquid nitrogen, the sample is irradiated at 370 nm for 30 minutes. The cuvette is then transferred into the precooled UV/Vis spectrometer (–185°C). After measuring the frozen solution (**Figure S76**), the sample is warmed to –130°C and a spectrum is measured after 5 minutes of thermal equilibration. Further spectra were measured at –30°C and room temperature. The spectra before irradiation, and after irradiation and warming to room temperature are identical with no absorption maxima in the visible range.

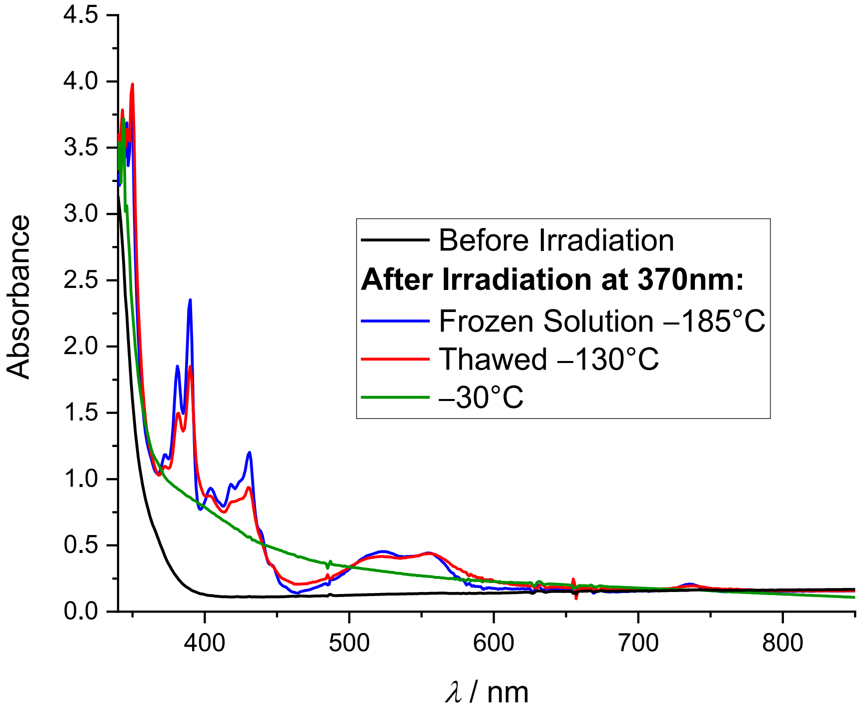

Figure S76: UV/Vis spectroscopic monitoring of the photolysis of **1** in 2-MeTHF in frozen solution and subsequent thawing. The final spectrum at room temperature is equal to the spectrum measured before irradiation.

2.8.2 NMR spectroscopy

Photolysis in liquid solution: A solution of **1** (17 mM) and styrene (1.5 eq) in toluene-D_8 was transferred into an NMR tube suitable for *in-situ* photolysis. After cooling the sample to −80°C, a reference spectrum was measured. Irradiation at 385 nm inside the spectrometer for 1 h (ca. 50% conversion) gives a paramagnetic species, which was also observed in the absence of styrene up to temperatures of –10°C.[1] The thermal stability of the paramagnetic product was monitored by NMR spectroscopy upon stepwise increasing the temperature in intervals of 10 K. The chemical shift of the photoproduct follows Curie behavior, as was previously reported.[1] Beyond −50 °C, reactivity with styrene is observed with concomitant formation of **3** and **8** as main products. Longer irradiation times, leading to full conversion of **1**, are accompanied by hydride (**7**) formation already at –80 °C.

Evans' method was employed to determine the effective magnetic moment (μ_{eff}) of the photoproduct. THF-D_8 was used as solvent and a capillary with PPh_3 in THF-D_8 as internal standard was added to the

NMR tube. For ^{1}H integration, 1,3,5-trimethoxybenzene (TMB) was added as an additional internal standard. The NMR tube was photolyzed at −85°C (acetone/LN_2) with a 370 nm Kessil LED for 10 min, inserted into the spectrometer and measured at −80°C to determine Δv. The broad signal at −10 ppm with an integral of 2 was integrated vs. the TMB signal to determine the concentration of paramagnetic species. Three independent measurements were performed for determination of μ_{eff} according to Sur's equation and corrected for diamagnetic contributions.[11] For S=1, a μ_{eff} (spin-only) of 2.83 is expected.

Table S5: Parameters for determination of μ_{eff} from Evans' NMR method. Spectrometer frequency v^0 = 400 MHz, T = 193K.

Measurement	Δv / Hz	c / mM	μ_{eff}
1	21	2.5	2.8
2	14	1.7	2.8
3	29	2.8	3.1
average			2.9

Photolysis in frozen-solution: A solution of **1** (17 mM) and styrene (1.5 eq) in toluene-D_8 was transferred into a J-Young NMR tube. The sample was frozen in liquid nitrogen, and a transparent glass was obtained. The sample was irradiated for 1 h (390 nm) at that that temperature. After that, the frozen solution was thawed at −75 °C in a cold bath (2-propanol/dry ice). NMR spectroscopic examination showed full consumption of **1** and formation of **3** and **8** as main products. Note that the time for full consumption of **1** strongly depends on the transparency of the glass.

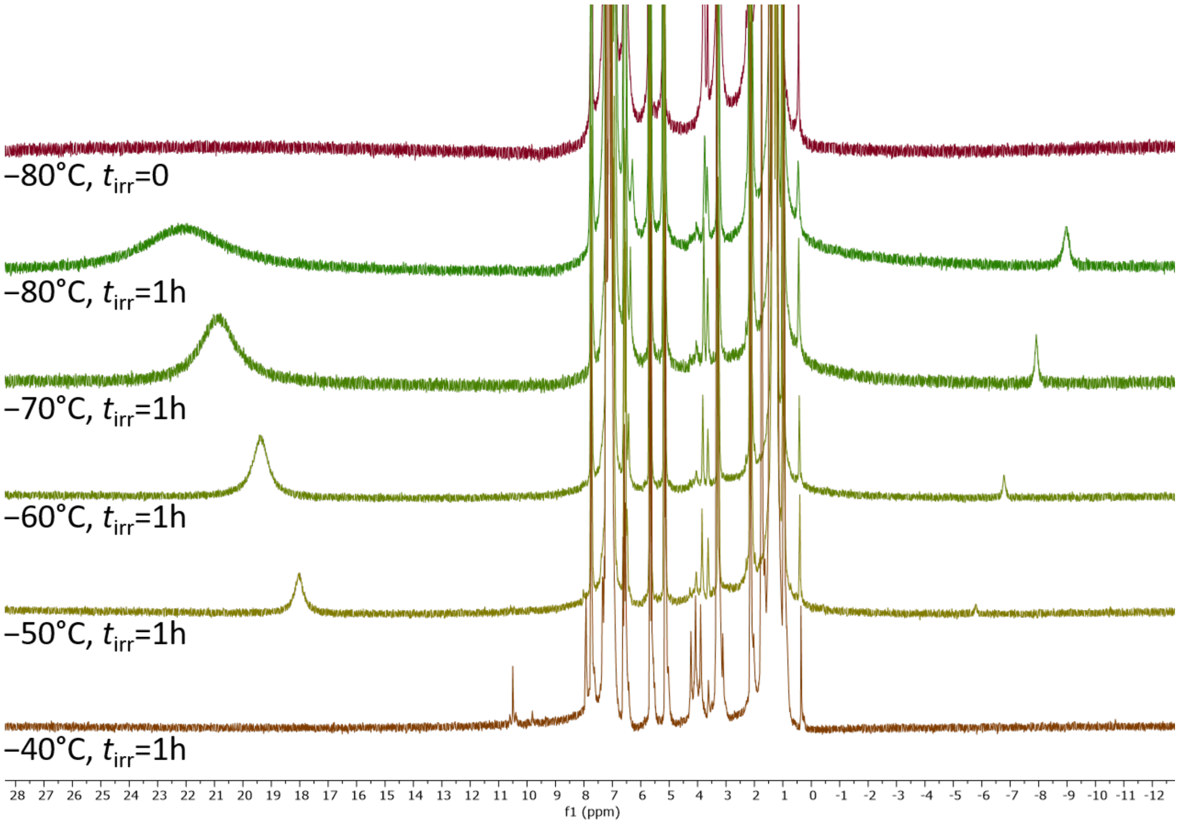

Figure S77: $^{1}H\{^{31}P\}$-NMR spectra of after photolysis of **1** in the presence of styrene in toluene-D_8. t_{irr} gives the total photolysis time. The signal at around δ = −130 ppm is not shown.

2.9 Photolysis of **1** in presence of **4**

1 (4.0 mg, 6.7 µmol, 1.0 eq.) and **4** (4.0 mg, 6.7 µmol, 1.0 eq.) are dissolved in toluene-D_8 (0.45 mL) in a J-Young NMR tube. The mixture is photolyzed at −30 °C (390 nm) for 5 minutes and examined by NMR spectroscopy.

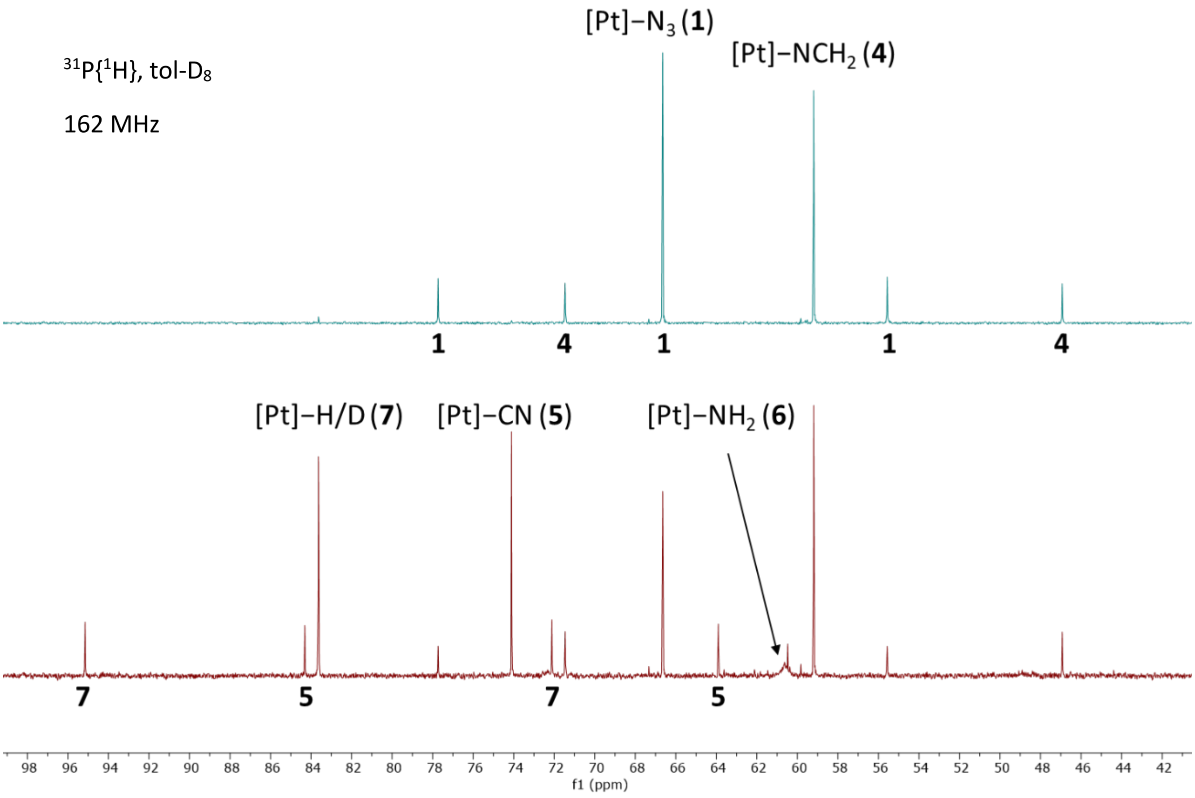

Figure S78: $^{31}P\{^1H\}$-NMR spectrum before (top) and after (bottom) photolysis (λ=390 nm) of **1** at −30°C.

2.10 Photolysis of **1** in the absence of substrate

1 was irradiated at 390 nm in a J-Young NMR tube at –30°C in THF-D_8 (17 mM) in the absence of other substrates with $OPPh_3$ as internal standard. NMR spectroscopic characterization shows the formation of Pt–H (no Pt–D) as the main product in 32% spectroscopic yield, besides several other unidentified phosphorous compounds. Furthermore, isobutene (5%) is observed in the $^{1}H\{^{31}P\}$-NMR spectrum, indicating partial photodegradation of the pincer ligand.

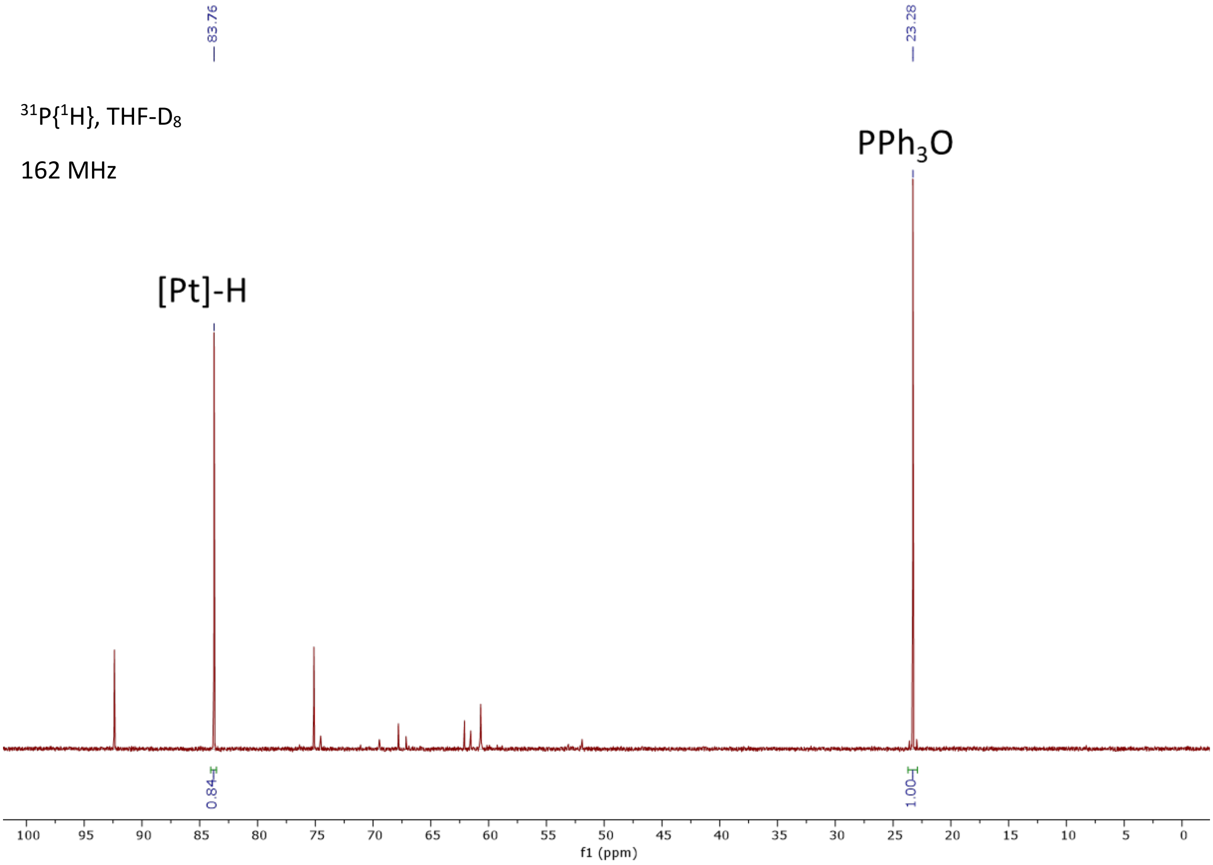

Figure S79: $^{31}P\{^{1}H\}$-NMR spectrum after irradiation (λ=390 nm) of **1** in THF-D_8 with $OPPh_3$ as internal standard at −30°C.

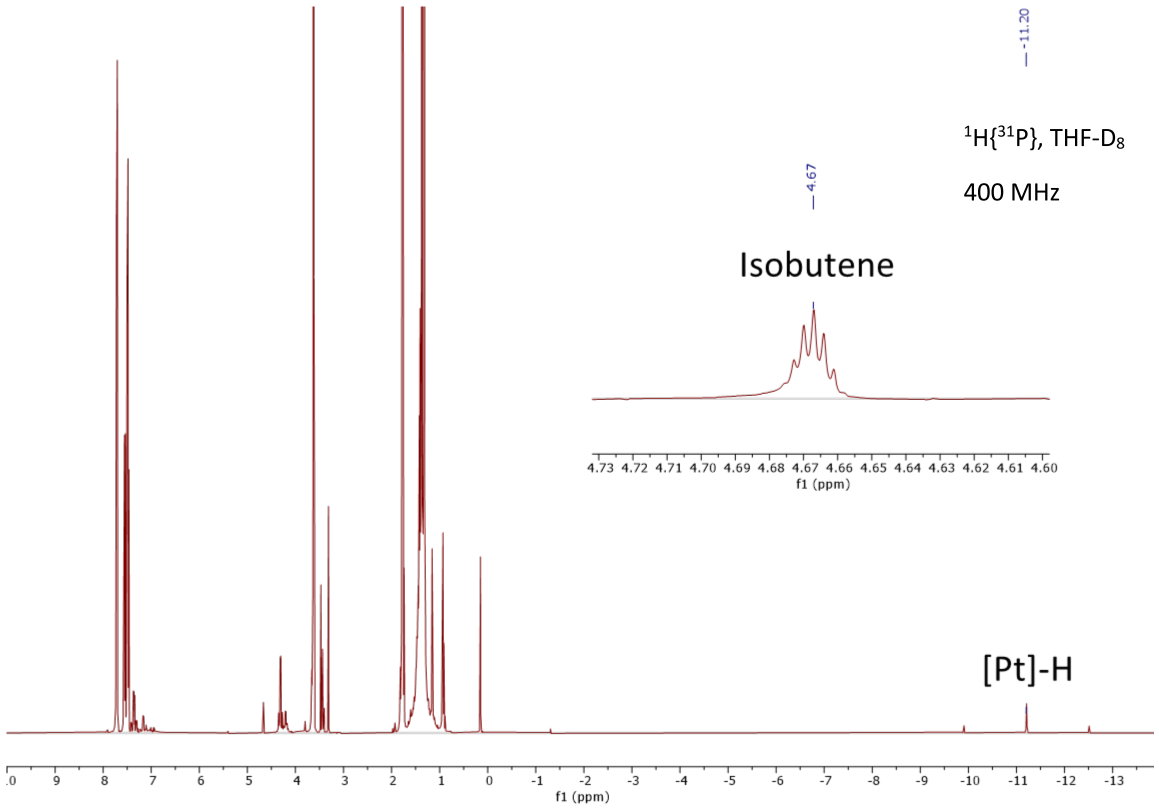

Figure S80: $^{1}H\{^{31}P\}$-NMR spectrum after irradiation (λ=390 nm) of **1** in THF-D_8 at −30°C.

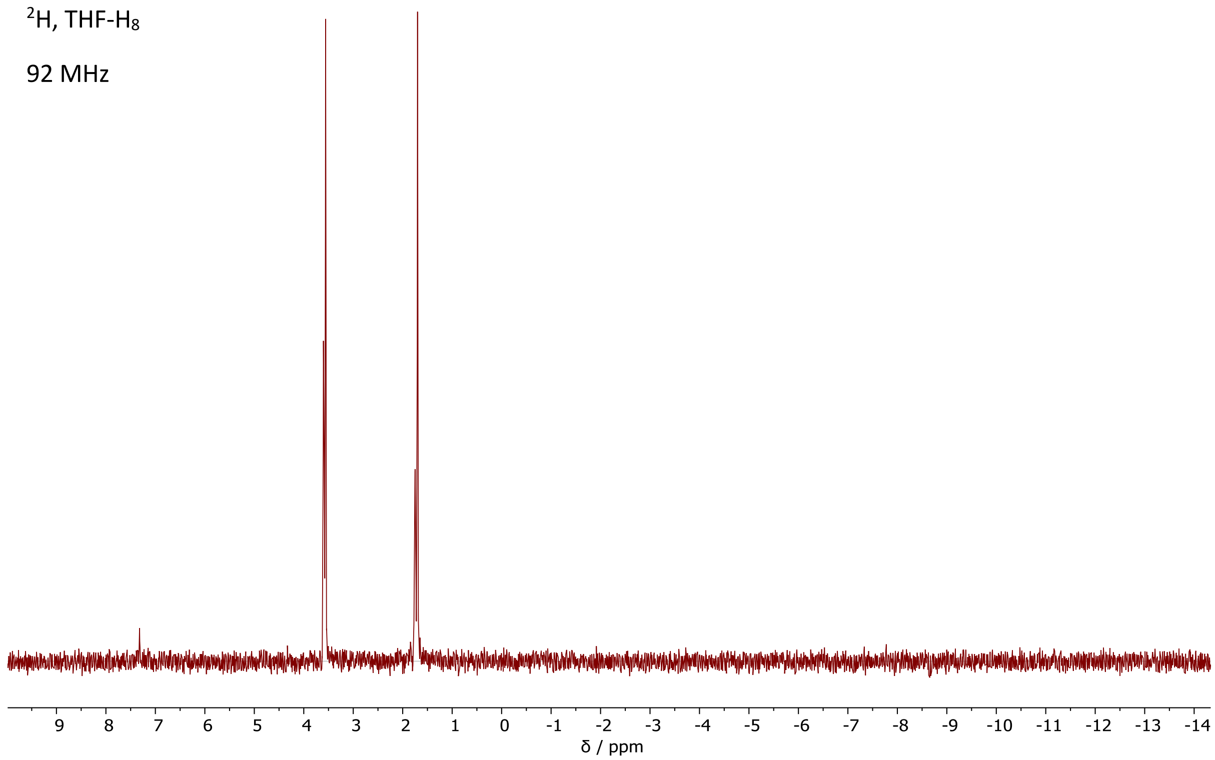

Figure S81: ^{2}H-NMR spectrum after irradiation (λ=390 nm) of **1** in THF-D_8 at −30°C, then removal of solvents in vacuo, then redissolved in THF-H_8.

3 EPR Spectroscopy

X-Band spectra were recorded with a Bruker ElexSys-II E500 CW-EPR spectrometer with frozen-solution samples in J-Young quartz glass EPR tubes, at temperatures between 130 and 150 K. Q-Band EPR measurements were performed on a Bruker ElexSys E580 EPR spectrometer equipped with a Bruker 3 mm cylindrical resonator in TE012 mode (QT-II) in a CF935 helium gas flow cryostat (Oxford Instruments, Abingdon, UK) at 80 K. EPR simulations were conducted using EasySpin's *pepper* function (6.0.0-dev.53).[12] For simulation of the experimental spectra, the computed hyperfine couplings (*A*) and computed Euler angles for **9** (Spin-Orbit ZORA) were used as initial guess and *g* and *A* values optimized manually. Euler angles were not optimized, since DFT calculations usually give accurate orientations of the *A*-tensor. The Euler angles are used in the *zyz* convention and describe a subsequent rotation of the frame: First by *α* about the z axis, yielding the frame x'y'z', then by *β* about the y' axis, yielding the frame x''y''z'', and last by *γ* about the z'' axis yielding the final frame. Positive angles refer to clockwise rotation.

Sample preparation: Styrene (2 eq.) was added to a solution of **1** in 2-MeTHF (1.5mg, 0.2mL, 13 mM). After transferring the mixture into a J-Young quartz EPR tube, the sample was frozen in liquid nitrogen to obtain a transparent glass. The sample was photolyzed for 3 h (390 nm LED) in a transparent dewar filled with liquid nitrogen. The frozen solution was then allowed to thaw at −90 °C in a cold bath (acetone/liquid nitrogen) for one minute before being frozen again.

Table S6: Experimental parameters for the measurements in Figure S82.

	T / K	MW Freq / GHz	Mod Freq / kHz	Mod Amp / G	MW power / mW	Scans
b	80	34.01	100	3	0.39	100
c-f	133	9.43	100	2	9.9	1

Table S7: Simulation and DFT parameters for X- and Q-Band simulation of **9**. Euler angles *α*, *β*, *γ* are given in degree.

	DFT (SO-ZORA)			Simulation		
g	2.106	2.000	1.985	2.125	2.003	1.993
A(Pt) / MHz	-473	-724	-494	-565	-530	-500
Euler angles	-12.0	9.3	28.0	-12.0	9.3	28.0
A(P_1) / MHz	61	60	80	60	60	80
Euler angles	25.9	29.5	22.0	25.9	29.5	22.0
A(P_2) / MHz	76	76	99	75	75	100
Euler angles	-4.7	29.7	24.3	-4.7	29.7	24.3
A(N) / MHz	12	6	109	12	6	109
Euler angles	46.8	6.2	-56.3	46.8	6.2	-56.3
A(H) / MHz	142	128	125	140	125	130
Euler angles	52.4	55.6	-47.4	52.4	55.6	-47.4
A(^{2}H) / MHz	–	–	–	20	20	20
Euler angles	–	–	–	52.4	55.6	-47.4
g-strain, X-Band<br>Q-Band	–	–	–	0.010<br>0.010	0.002<br>0.002	0.005<br>0.005
H-strain, X-Band<br>Q-Band	–	–	–	10<br>20	10<br>20	10<br>20
line width / mT (isotropic)	–			0.7 (X-Band), 1.2 (Q-Band)		

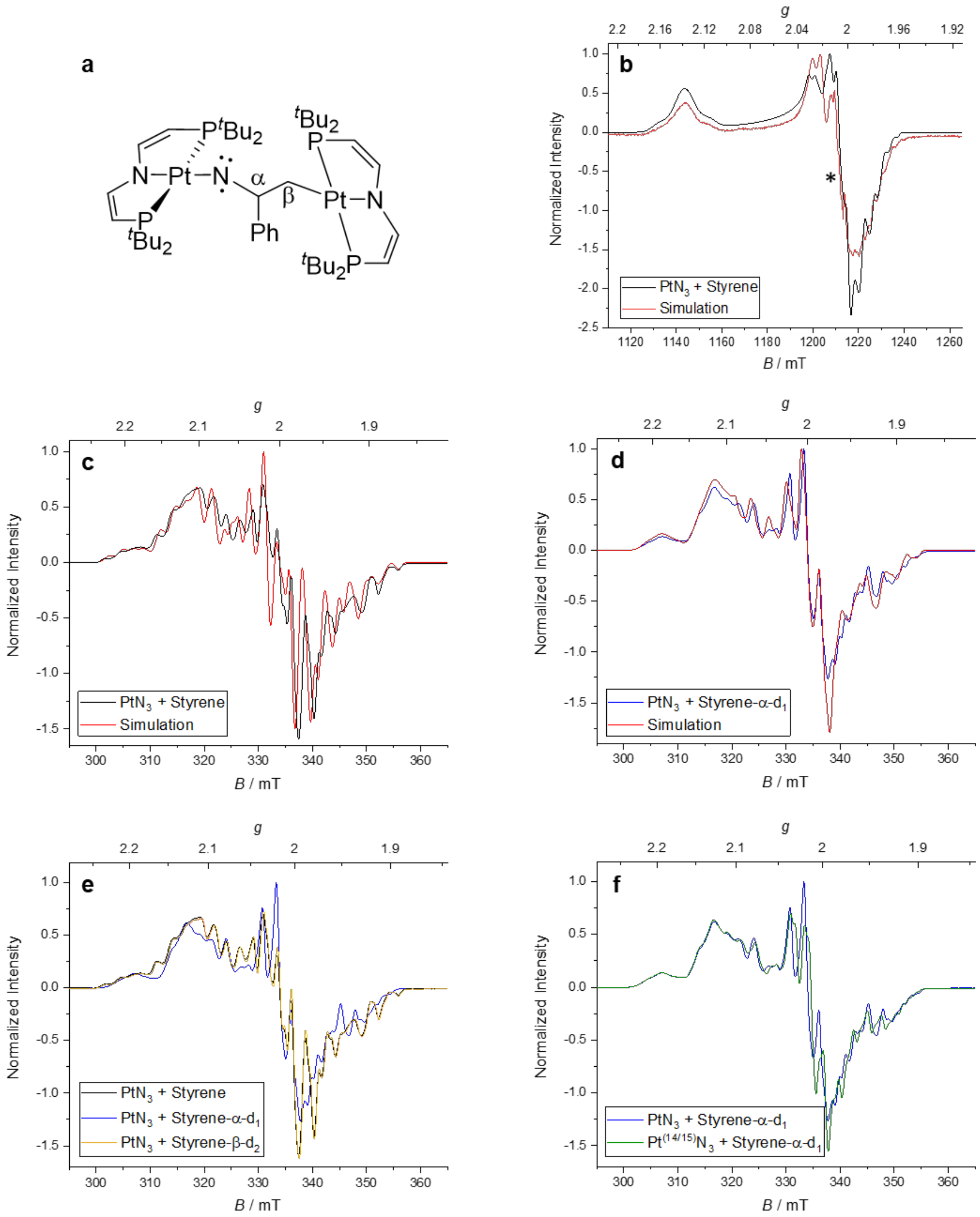

Figure S82: Experimental and simulated EPR spectra of the frozen reaction mixtures after photolysis of **1** with styrene, in MeTHF. b) Experimental and simulated Q-band spectrum; asterisk denotes glass peak. c-d) Experimental and simulated X-Band spectra with styrene and α-d$_1$-labelled styrene. e-f) Experimental X-Band spectra with differently labelled styrene and ^{15}N labelled azide **1** (50%). The computed spin density plot of **9** is shown in **Figure S94**.

4 Crystallographic Details

CCDC 2339415 (**3**-CF_3), CCDC 2339416 (**3**-OMe) and CCDC 2339414 (**4**) contain the supplementary crystallographic data for this paper. These data can be obtained free of charge from https://www.ccdc.cam.ac.uk/structures/ (or from Cambridge Crystallographic Data Centre, 12 Union Road, Cambridge, CB2 1EZ, UK. Fax: +44-1223-336-033; e-mail: deposit@ccdc.cam.ac.uk).

Suitable single crystals for X-ray structure determination were selected from the mother liquor under an inert gas atmosphere and transferred in protective perfluoro polyether oil on a microscope slide. The selected and mounted crystals were transferred to the cold gas stream on the diffractometer. The diffraction data were obtained at 100 K on a Bruker D8 three-circle diffractometer, equipped with a PHOTON III detector or a PHOTON 100 CMOS detector and an INCOATEC microfocus source with Quazar mirror optics (Mo-Kα radiation, λ= 0.71073 Å).

The data were integrated with SAINT and a semi-empirical absorption correction from equivalents with SADABS was applied. The structure was solved and refined using the Bruker SHELX 2014 software package.[13–16] All non-hydrogen atoms were refined with anisotropic displacement parameters. All C–H hydrogen atoms were refined isotropically on calculated positions by using a riding model with their U_{iso} values constrained to 1.5 U_{eq} of their pivot atoms for terminal sp^3 carbon atoms and 1.2 times for all other atoms.

4.1 Crystal Structure of [(PNP)Pt(NCH(C_6H_4OMe)] (**3**-OMe)

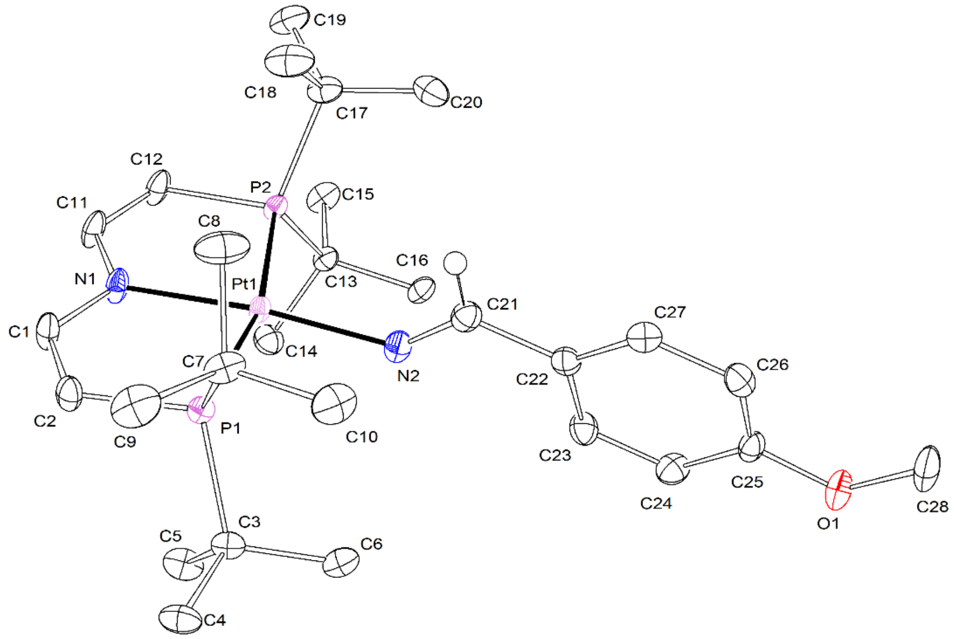

Figure S83: Thermal ellipsoid plot of **3**-OMe with the anisotropic displacement parameters drawn at the 50% probability level. The asymmetric unit contains one complex molecule.

Table S8: Crystal data and structure refinement for **3**-OMe.

Identification code	mo_CW_JS_220810_2_0m_a (JS-5-49)	
Empirical formula	$C_{28}H_{48}N_2OP_2Pt$	
Formula weight	685.71	
Temperature	100(2) K	
Wavelength	0.71073 Å	
Crystal system	Triclinic	
Space group	$P\bar{1}$	
Unit cell dimensions	a = 8.4025(3) Å	α= 91.863(3)°
	b = 12.4446(6) Å	β= 94.111(2)°
	c = 14.3129(6) Å	γ = 90.429(3)°
Volume	1491.93(11) Å^3	
Z	2	
Density (calculated)	1.526 Mg/m^3	
Absorption coefficient	4.832 mm^{-1}	
F(000)	692	
Crystal size	0.172 x 0.123 x 0.112 mm^3	
Crystal shape and color	Plate, clear light yellow	
Theta range for data collection	2.430 to 28.411°	
Index ranges	-11<=h<=11, -16<=k<=16, -19<=l<=19	
Reflections collected	79024	

Independent reflections	7480 [R(int) = 0.0726]
Completeness to theta = 25.242°	99.9 %
Refinement method	Full-matrix least-squares on F^2
Data / restraints / parameters	7480 / 0 / 320
Goodness-of-fit on F^2	1.070
Final R indices [I>2sigma(I)]	R1 = 0.0238, wR2 = 0.0430
R indices (all data)	R1 = 0.0319, wR2 = 0.0456
Largest diff. peak and hole	1.228 and -1.243 eÅ^{-3}

Table S9: Bond lengths [Å] and angles [°] for **3**-OMe.

Pt(1)-N(2)	1.989(2)	C(17)-C(18)	1.538(4)
Pt(1)-N(1)	2.043(2)	C(21)-C(22)	1.491(4)
Pt(1)-P(2)	2.3023(7)	C(22)-C(27)	1.383(4)
Pt(1)-P(1)	2.3030(7)	C(22)-C(23)	1.401(4)
P(1)-C(2)	1.788(3)	C(23)-C(24)	1.371(4)
P(1)-C(3)	1.872(3)	C(24)-C(25)	1.392(4)
P(1)-C(7)	1.874(3)	C(25)-C(26)	1.382(4)
P(2)-C(12)	1.783(3)	C(26)-C(27)	1.395(4)
P(2)-C(13)	1.869(3)		
P(2)-C(17)	1.874(3)	N(2)-Pt(1)-N(1)	172.57(10)
O(1)-C(25)	1.372(3)	N(2)-Pt(1)-P(2)	97.49(7)
O(1)-C(28)	1.419(4)	N(1)-Pt(1)-P(2)	82.90(6)
N(1)-C(1)	1.358(3)	N(2)-Pt(1)-P(1)	96.74(7)
N(1)-C(11)	1.364(3)	N(1)-Pt(1)-P(1)	82.97(6)
N(2)-C(21)	1.253(4)	P(2)-Pt(1)-P(1)	165.77(2)
C(1)-C(2)	1.351(4)	C(2)-P(1)-C(3)	106.80(13)
C(3)-C(4)	1.524(4)	C(2)-P(1)-C(7)	104.82(13)
C(3)-C(6)	1.534(4)	C(3)-P(1)-C(7)	112.45(12)
C(3)-C(5)	1.537(4)	C(2)-P(1)-Pt(1)	99.84(9)
C(7)-C(10)	1.526(4)	C(3)-P(1)-Pt(1)	114.75(9)
C(7)-C(9)	1.528(4)	C(7)-P(1)-Pt(1)	116.31(9)
C(7)-C(8)	1.530(4)	C(12)-P(2)-C(13)	106.69(13)
C(11)-C(12)	1.347(4)	C(12)-P(2)-C(17)	105.20(14)
C(13)-C(16)	1.530(4)	C(13)-P(2)-C(17)	112.58(12)
C(13)-C(15)	1.531(4)	C(12)-P(2)-Pt(1)	99.96(9)
C(13)-C(14)	1.536(4)	C(13)-P(2)-Pt(1)	114.82(8)
C(17)-C(20)	1.527(4)	C(17)-P(2)-Pt(1)	115.80(9)
C(17)-C(19)	1.529(4)	C(25)-O(1)-C(28)	116.9(2)

C(1)-N(1)-C(11)	121.5(2)	C(15)-C(13)-C(14)	109.6(2)
C(1)-N(1)-Pt(1)	119.32(18)	C(16)-C(13)-P(2)	110.75(18)
C(11)-N(1)-Pt(1)	119.19(18)	C(15)-C(13)-P(2)	112.88(18)
C(21)-N(2)-Pt(1)	130.3(2)	C(14)-C(13)-P(2)	104.97(18)
C(2)-C(1)-N(1)	122.4(3)	C(20)-C(17)-C(19)	110.1(2)
C(1)-C(2)-P(1)	115.5(2)	C(20)-C(17)-C(18)	108.8(3)
C(4)-C(3)-C(6)	110.6(2)	C(19)-C(17)-C(18)	108.7(2)
C(4)-C(3)-C(5)	109.2(2)	C(20)-C(17)-P(2)	111.97(19)
C(6)-C(3)-C(5)	107.7(2)	C(19)-C(17)-P(2)	112.3(2)
C(4)-C(3)-P(1)	113.44(19)	C(18)-C(17)-P(2)	104.84(19)
C(6)-C(3)-P(1)	110.51(18)	N(2)-C(21)-C(22)	121.3(3)
C(5)-C(3)-P(1)	105.03(19)	C(27)-C(22)-C(23)	117.9(3)
C(10)-C(7)-C(9)	109.6(2)	C(27)-C(22)-C(21)	121.7(3)
C(10)-C(7)-C(8)	109.3(3)	C(23)-C(22)-C(21)	120.4(2)
C(9)-C(7)-C(8)	108.7(2)	C(24)-C(23)-C(22)	120.7(3)
C(10)-C(7)-P(1)	112.09(19)	C(23)-C(24)-C(25)	120.7(3)
C(9)-C(7)-P(1)	112.3(2)	O(1)-C(25)-C(26)	125.0(3)
C(8)-C(7)-P(1)	104.70(19)	O(1)-C(25)-C(24)	115.2(3)
C(12)-C(11)-N(1)	122.3(3)	C(26)-C(25)-C(24)	119.8(3)
C(11)-C(12)-P(2)	115.6(2)	C(25)-C(26)-C(27)	118.9(3)
C(16)-C(13)-C(15)	110.0(2)	C(22)-C(27)-C(26)	122.0(3)
C(16)-C(13)-C(14)	108.4(2)		

Symmetry transformations used to generate equivalent atoms:

Table S10: Torsion angles [°] for 3-OMe.

C(11)-N(1)-C(1)-C(2)	179.5(3)	Pt(1)-P(1)-C(3)-C(6)	58.8(2)
Pt(1)-N(1)-C(1)-C(2)	0.8(4)	C(2)-P(1)-C(3)-C(5)	52.5(2)
N(1)-C(1)-C(2)-P(1)	0.1(4)	C(7)-P(1)-C(3)-C(5)	166.93(18)
C(3)-P(1)-C(2)-C(1)	-120.5(2)	Pt(1)-P(1)-C(3)-C(5)	-57.1(2)
C(7)-P(1)-C(2)-C(1)	120.0(2)	C(2)-P(1)-C(7)-C(10)	164.8(2)
Pt(1)-P(1)-C(2)-C(1)	-0.7(2)	C(3)-P(1)-C(7)-C(10)	49.2(2)
C(2)-P(1)-C(3)-C(4)	-66.7(2)	Pt(1)-P(1)-C(7)-C(10)	-86.0(2)
C(7)-P(1)-C(3)-C(4)	47.8(2)	C(2)-P(1)-C(7)-C(9)	41.0(2)
Pt(1)-P(1)-C(3)-C(4)	-176.32(17)	C(3)-P(1)-C(7)-C(9)	-74.7(2)
C(2)-P(1)-C(3)-C(6)	168.38(19)	Pt(1)-P(1)-C(7)-C(9)	150.13(18)
C(7)-P(1)-C(3)-C(6)	-77.2(2)	C(2)-P(1)-C(7)-C(8)	-76.8(2)

C(3)-P(1)-C(7)-C(8)	167.6(2)	C(13)-P(2)-C(17)-C(19)	73.6(2)
Pt(1)-P(1)-C(7)-C(8)	32.4(2)	Pt(1)-P(2)-C(17)-C(19)	-151.47(17)
C(1)-N(1)-C(11)-C(12)	179.8(3)	C(12)-P(2)-C(17)-C(18)	75.6(2)
Pt(1)-N(1)-C(11)-C(12)	-1.5(4)	C(13)-P(2)-C(17)-C(18)	-168.6(2)
N(1)-C(11)-C(12)-P(2)	-0.3(4)	Pt(1)-P(2)-C(17)-C(18)	-33.7(2)
C(13)-P(2)-C(12)-C(11)	121.4(2)	Pt(1)-N(2)-C(21)-C(22)	175.27(18)
C(17)-P(2)-C(12)-C(11)	-118.8(2)	N(2)-C(21)-C(22)-C(27)	-171.8(3)
Pt(1)-P(2)-C(12)-C(11)	1.6(3)	N(2)-C(21)-C(22)-C(23)	7.1(4)
C(12)-P(2)-C(13)-C(16)	-172.19(19)	C(27)-C(22)-C(23)-C(24)	-0.9(4)
C(17)-P(2)-C(13)-C(16)	72.9(2)	C(21)-C(22)-C(23)-C(24)	-179.7(3)
Pt(1)-P(2)-C(13)-C(16)	-62.46(19)	C(22)-C(23)-C(24)-C(25)	0.3(4)
C(12)-P(2)-C(13)-C(15)	63.9(2)	C(28)-O(1)-C(25)-C(26)	1.2(4)
C(17)-P(2)-C(13)-C(15)	-51.0(2)	C(28)-O(1)-C(25)-C(24)	179.7(3)
Pt(1)-P(2)-C(13)-C(15)	173.64(17)	C(23)-C(24)-C(25)-O(1)	-178.1(3)
C(12)-P(2)-C(13)-C(14)	-55.4(2)	C(23)-C(24)-C(25)-C(26)	0.4(4)
C(17)-P(2)-C(13)-C(14)	-170.32(18)	O(1)-C(25)-C(26)-C(27)	177.8(2)
Pt(1)-P(2)-C(13)-C(14)	54.31(19)	C(24)-C(25)-C(26)-C(27)	-0.6(4)
C(12)-P(2)-C(17)-C(20)	-166.6(2)	C(23)-C(22)-C(27)-C(26)	0.7(4)
C(13)-P(2)-C(17)-C(20)	-50.8(2)	C(21)-C(22)-C(27)-C(26)	179.5(2)
Pt(1)-P(2)-C(17)-C(20)	84.1(2)	C(25)-C(26)-C(27)-C(22)	0.0(4)
C(12)-P(2)-C(17)-C(19)	-42.2(2)		

Symmetry transformations used to generate equivalent atoms:

4.2 Crystal Structure of [(PNP)Pt(NCH(C_6H_4OMe)] (**3**-CF_3)

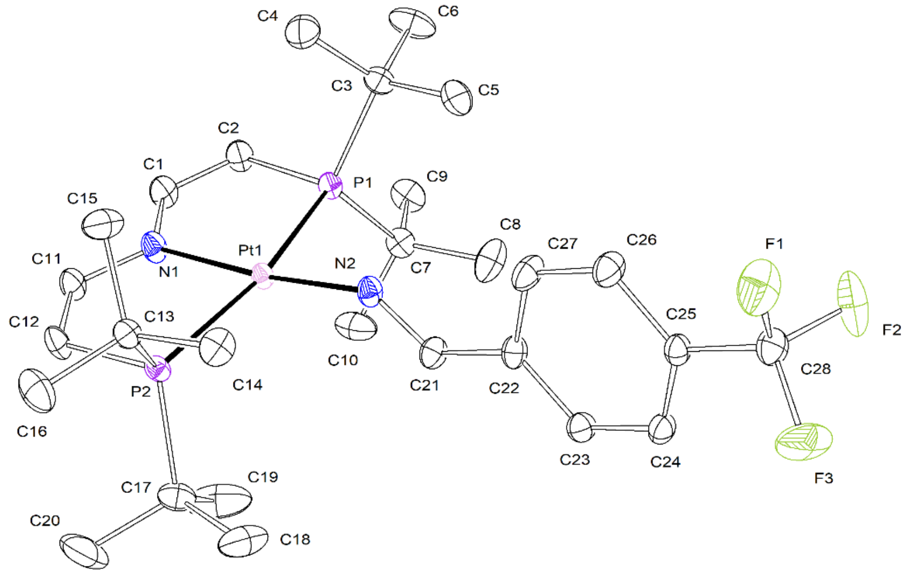

Figure S84: Thermal ellipsoid plot of **3**-CF_3 with the anisotropic displacement parameters drawn at the 50% probability level. The asymmetric unit contains one complex molecule.

Table S11: Crystal data and structure refinement for **3**-CF_3.

Identification code	JS_051021_MO_a	(JS-7-183)
Empirical formula	$C_{28}H_{45}F_3N_2P_2Pt$	
Formula weight	723.69	
Temperature	120(2) K	
Wavelength	0.71073 Å	
Crystal system	Monoclinic	
Space group	$P2_1/c$	
Unit cell dimensions	a = 11.8636(7) Å	α= 90°
	b = 16.2135(10) Å	β= 90.727(2)°
	c = 15.6914(10) Å	γ = 90°
Volume	3018.0(3) Å^3	
Z	4	
Density (calculated)	1.593 Mg/m^3	
Absorption coefficient	4.793 mm^{-1}	
F(000)	1448	
Crystal size	0.087 x 0.040 x 0.024 mm^3	
Crystal shape and color	Block, clear intense orange-yellow	
Theta range for data collection	2.127 to 41.192°.	

Index ranges	-21<=h<=21, -29<=k<=30, -29<=l<=29
Reflections collected	217536
Independent reflections	20051 [R(int) = 0.0977]
Completeness to theta = 25.242°	100.0 %
Refinement method	Full-matrix least-squares on F^2
Data / restraints / parameters	20051 / 0 / 337
Goodness-of-fit on F^2	1.062
Final R indices [I>2sigma(I)]	R1 = 0.0457, wR2 = 0.0671
R indices (all data)	R1 = 0.0834, wR2 = 0.0792
Largest diff. peak and hole	1.481 and -2.574 e.Å^{-3}

Table S12: Bond lengths [Å] and angles [°] for **3**-CF_3.

Pt(1)-N(2)	1.989(2)	C(13)-C(15)	1.534(4)
Pt(1)-N(1)	2.043(2)	C(17)-C(18)	1.509(5)
Pt(1)-P(2)	2.3063(7)	C(17)-C(19)	1.523(5)
Pt(1)-P(1)	2.3084(7)	C(17)-C(20)	1.534(5)
F(1)-C(28)	1.343(4)	C(21)-C(22)	1.489(3)
P(1)-C(2)	1.783(3)	C(22)-C(27)	1.396(4)
P(1)-C(3)	1.867(3)	C(22)-C(23)	1.396(4)
P(1)-C(7)	1.874(3)	C(23)-C(24)	1.386(4)
N(1)-C(1)	1.364(4)	C(24)-C(25)	1.382(4)
N(1)-C(11)	1.366(4)	C(25)-C(26)	1.394(4)
C(1)-C(2)	1.355(4)	C(25)-C(28)	1.497(4)
P(2)-C(12)	1.787(3)	C(26)-C(27)	1.385(4)
P(2)-C(13)	1.867(3)		
P(2)-C(17)	1.875(3)	N(2)-Pt(1)-N(1)	172.57(10)
F(2)-C(28)	1.338(4)	N(2)-Pt(1)-P(2)	97.36(7)
N(2)-C(21)	1.261(3)	N(1)-Pt(1)-P(2)	82.73(7)
C(3)-C(4)	1.530(4)	N(2)-Pt(1)-P(1)	97.69(7)
C(3)-C(5)	1.530(4)	N(1)-Pt(1)-P(1)	82.37(7)
C(3)-C(6)	1.532(4)	P(2)-Pt(1)-P(1)	164.95(2)
F(3)-C(28)	1.323(4)	C(2)-P(1)-C(3)	106.71(14)
C(7)-C(8)	1.531(4)	C(2)-P(1)-C(7)	104.31(14)
C(7)-C(9)	1.532(4)	C(3)-P(1)-C(7)	113.67(13)
C(7)-C(10)	1.534(4)	C(2)-P(1)-Pt(1)	100.50(10)
C(11)-C(12)	1.348(4)	C(3)-P(1)-Pt(1)	114.48(9)
C(13)-C(16)	1.529(4)	C(7)-P(1)-Pt(1)	115.25(10)
C(13)-C(14)	1.530(4)	C(1)-N(1)-C(11)	120.7(2)

C(1)-N(1)-Pt(1)	119.84(19)	C(16)-C(13)-C(15)	109.4(3)
C(11)-N(1)-Pt(1)	119.50(19)	C(14)-C(13)-C(15)	108.3(2)
C(2)-C(1)-N(1)	122.2(3)	C(16)-C(13)-P(2)	113.0(2)
C(2)-C(1)-H(1)	118.9	C(14)-C(13)-P(2)	111.39(19)
N(1)-C(1)-H(1)	118.9	C(15)-C(13)-P(2)	105.22(19)
C(12)-P(2)-C(13)	106.12(14)	C(18)-C(17)-C(19)	110.2(3)
C(12)-P(2)-C(17)	105.41(14)	C(18)-C(17)-C(20)	109.5(3)
C(13)-P(2)-C(17)	113.08(13)	C(19)-C(17)-C(20)	108.0(3)
C(12)-P(2)-Pt(1)	100.09(10)	C(18)-C(17)-P(2)	112.5(2)
C(13)-P(2)-Pt(1)	114.88(9)	C(19)-C(17)-P(2)	104.8(2)
C(17)-P(2)-Pt(1)	115.39(10)	C(20)-C(17)-P(2)	111.7(3)
C(21)-N(2)-Pt(1)	130.76(19)	N(2)-C(21)-C(22)	121.2(2)
C(1)-C(2)-P(1)	115.0(2)	C(27)-C(22)-C(23)	118.3(2)
C(4)-C(3)-C(5)	108.5(3)	C(27)-C(22)-C(21)	120.1(2)
C(4)-C(3)-C(6)	109.5(3)	C(23)-C(22)-C(21)	121.6(2)
C(5)-C(3)-C(6)	109.2(3)	C(24)-C(23)-C(22)	121.2(2)
C(4)-C(3)-P(1)	105.0(2)	C(25)-C(24)-C(23)	119.8(2)
C(5)-C(3)-P(1)	111.1(2)	C(24)-C(25)-C(26)	120.0(2)
C(6)-C(3)-P(1)	113.3(2)	C(24)-C(25)-C(28)	120.6(2)
C(8)-C(7)-C(9)	109.2(3)	C(26)-C(25)-C(28)	119.4(2)
C(8)-C(7)-C(10)	109.4(3)	C(27)-C(26)-C(25)	120.0(3)
C(9)-C(7)-C(10)	108.4(3)	C(26)-C(27)-C(22)	120.8(3)
C(8)-C(7)-P(1)	111.7(2)	F(3)-C(28)-F(2)	106.9(3)
C(9)-C(7)-P(1)	112.9(2)	F(3)-C(28)-F(1)	106.6(3)
C(10)-C(7)-P(1)	105.1(2)	F(2)-C(28)-F(1)	104.9(3)
C(12)-C(11)-N(1)	122.2(2)	F(3)-C(28)-C(25)	112.9(2)
C(11)-C(12)-P(2)	115.5(2)	F(2)-C(28)-C(25)	112.6(3)
C(16)-C(13)-C(14)	109.3(2)	F(1)-C(28)-C(25)	112.3(2)

Symmetry transformations used to generate equivalent atoms:

Table S13: Torsion angles [°] for **3**-CF_3.

C(11)-N(1)-C(1)-C(2)	179.8(3)
Pt(1)-N(1)-C(1)-C(2)	1.2(4)
N(1)-C(1)-C(2)-P(1)	1.6(4)
C(3)-P(1)-C(2)-C(1)	-122.8(3)
C(7)-P(1)-C(2)-C(1)	116.6(3)
Pt(1)-P(1)-C(2)-C(1)	-3.1(3)
C(2)-P(1)-C(3)-C(4)	55.5(2)
C(7)-P(1)-C(3)-C(4)	169.9(2)
Pt(1)-P(1)-C(3)-C(4)	-54.7(2)
C(2)-P(1)-C(3)-C(5)	172.6(2)
C(7)-P(1)-C(3)-C(5)	-73.0(2)
Pt(1)-P(1)-C(3)-C(5)	62.3(2)
C(2)-P(1)-C(3)-C(6)	-64.0(3)
C(7)-P(1)-C(3)-C(6)	50.5(3)
Pt(1)-P(1)-C(3)-C(6)	-174.2(2)
C(2)-P(1)-C(7)-C(8)	170.1(2)
C(3)-P(1)-C(7)-C(8)	54.3(3)
Pt(1)-P(1)-C(7)-C(8)	-80.7(2)
C(2)-P(1)-C(7)-C(9)	46.6(2)
C(3)-P(1)-C(7)-C(9)	-69.2(2)
Pt(1)-P(1)-C(7)-C(9)	155.77(19)
C(2)-P(1)-C(7)-C(10)	-71.3(2)
C(3)-P(1)-C(7)-C(10)	172.8(2)
Pt(1)-P(1)-C(7)-C(10)	37.8(2)
C(1)-N(1)-C(11)-C(12)	-179.3(3)
Pt(1)-N(1)-C(11)-C(12)	-0.7(4)
N(1)-C(11)-C(12)-P(2)	-0.2(4)
C(13)-P(2)-C(12)-C(11)	120.5(3)
C(17)-P(2)-C(12)-C(11)	-119.3(3)
Pt(1)-P(2)-C(12)-C(11)	0.8(3)
C(12)-P(2)-C(13)-C(16)	60.3(2)
C(17)-P(2)-C(13)-C(16)	-54.8(3)
Pt(1)-P(2)-C(13)-C(16)	169.9(2)
C(12)-P(2)-C(13)-C(14)	-176.1(2)
C(17)-P(2)-C(13)-C(14)	68.8(2)
Pt(1)-P(2)-C(13)-C(14)	-66.6(2)
C(12)-P(2)-C(13)-C(15)	-59.0(2)
C(17)-P(2)-C(13)-C(15)	-174.1(2)
Pt(1)-P(2)-C(13)-C(15)	50.6(2)
C(12)-P(2)-C(17)-C(18)	-162.9(3)
C(13)-P(2)-C(17)-C(18)	-47.4(3)
Pt(1)-P(2)-C(17)-C(18)	87.7(3)
C(12)-P(2)-C(17)-C(19)	77.3(3)
C(13)-P(2)-C(17)-C(19)	-167.1(3)
Pt(1)-P(2)-C(17)-C(19)	-32.0(3)
C(12)-P(2)-C(17)-C(20)	-39.3(3)
C(13)-P(2)-C(17)-C(20)	76.2(3)
Pt(1)-P(2)-C(17)-C(20)	-148.7(2)
Pt(1)-N(2)-C(21)-C(22)	179.9(2)
N(2)-C(21)-C(22)-C(27)	-7.2(5)
N(2)-C(21)-C(22)-C(23)	172.8(3)
C(27)-C(22)-C(23)-C(24)	1.1(4)
C(21)-C(22)-C(23)-C(24)	-178.9(3)
C(22)-C(23)-C(24)-C(25)	-0.5(4)
C(23)-C(24)-C(25)-C(26)	-0.5(4)
C(23)-C(24)-C(25)-C(28)	-179.3(3)
C(24)-C(25)-C(26)-C(27)	1.0(5)
C(28)-C(25)-C(26)-C(27)	179.8(3)
C(25)-C(26)-C(27)-C(22)	-0.4(5)
C(23)-C(22)-C(27)-C(26)	-0.6(5)
C(21)-C(22)-C(27)-C(26)	179.4(3)
C(24)-C(25)-C(28)-F(3)	-33.2(4)
C(26)-C(25)-C(28)-F(3)	147.9(3)
C(24)-C(25)-C(28)-F(2)	88.0(3)
C(26)-C(25)-C(28)-F(2)	-90.8(3)
C(24)-C(25)-C(28)-F(1)	-153.8(3)
C(26)-C(25)-C(28)-F(1)	27.4(4)

Symmetry transformations used to generate equivalent atoms:

4.3 Crystal Structure of [(PNP)Pt(NCH$_2$)] (4)

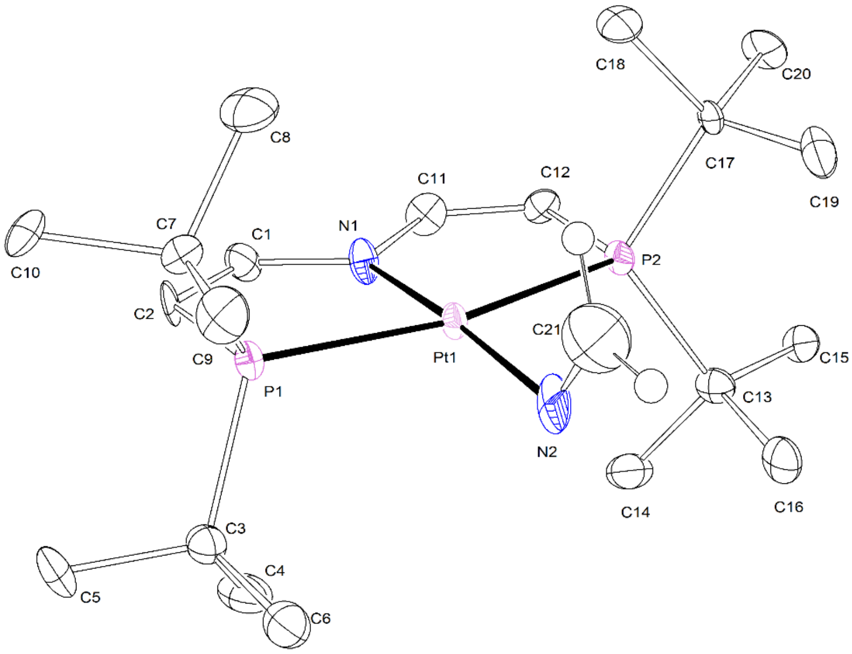

Figure S85: Thermal ellipsoid plot of **4** with the anisotropic displacement parameters drawn at the 50% probability level. The asymmetric unit contains one complex molecule.

Table S14: Crystal data and structure refinement for **4**.

Identification code	mo_CW_JS_220819_0m_a (JS-5-44)	
Empirical formula	$C_{21}H_{42}N_2P_2Pt$	
Formula weight	579.59	
Temperature	100(2) K	
Wavelength	0.71073 Å	
Crystal system	Monoclinic	
Space group	$P2_1/c$	
Unit cell dimensions	a = 11.9178(7) Å	α= 90°
	b = 16.4652(10) Å	β= 104.566(3)°
	c = 12.5288(8) Å	γ = 90°
Volume	2379.5(3) Å^3	
Z	4	
Density (calculated)	1.618 Mg/m^3	
Absorption coefficient	6.039 mm^{-1}	
F(000)	1160	
Crystal size	0.245 x 0.135 x 0.084 mm^3	
Crystal shape and color	Plate, clear light yellow	
Theta range for data collection	2.156 to 27.203°	
Index ranges	-15<=h<=15, -21<=k<=21, -16<=l<=16	
Reflections collected	79650	

Independent reflections	5294 [R(int) = 0.1762]
Completeness to theta = 25.242°	99.9 %
Refinement method	Full-matrix least-squares on F^2
Data / restraints / parameters	5294 / 0 / 247
Goodness-of-fit on F^2	1.182
Final R indices [I>2sigma(I)]	R1 = 0.0546, wR2 = 0.0753
R indices (all data)	R1 = 0.0926, wR2 = 0.0857
Largest diff. peak and hole	1.287 and -1.294 eÅ^{-3}

Table S15: Bond lengths [Å] and angles [°] for **4**.

Pt(1)-N(2)	2.001(7)	N(2)-Pt(1)-N(1)	174.8(3)
Pt(1)-N(1)	2.043(6)	N(2)-Pt(1)-P(1)	97.06(18)
Pt(1)-P(1)	2.2946(17)	N(1)-Pt(1)-P(1)	82.98(17)
Pt(1)-P(2)	2.3046(17)	N(2)-Pt(1)-P(2)	97.18(18)
P(1)-C(2)	1.782(7)	N(1)-Pt(1)-P(2)	82.97(17)
P(1)-C(3)	1.877(8)	P(1)-Pt(1)-P(2)	165.68(6)
P(1)-C(7)	1.881(8)	C(2)-P(1)-C(3)	105.7(3)
P(2)-C(12)	1.792(7)	C(2)-P(1)-C(7)	106.2(4)
P(2)-C(17)	1.866(8)	C(3)-P(1)-C(7)	112.5(3)
P(2)-C(13)	1.874(8)	C(2)-P(1)-Pt(1)	99.9(2)
N(1)-C(1)	1.361(9)	C(3)-P(1)-Pt(1)	114.3(2)
N(1)-C(11)	1.367(9)	C(7)-P(1)-Pt(1)	116.4(3)
N(2)-C(21)	1.208(12)	C(12)-P(2)-C(17)	105.2(3)
C(1)-C(2)	1.345(10)	C(12)-P(2)-C(13)	106.3(3)
C(3)-C(4)	1.522(11)	C(17)-P(2)-C(13)	112.6(3)
C(3)-C(5)	1.539(10)	C(12)-P(2)-Pt(1)	100.0(2)
C(3)-C(6)	1.539(10)	C(17)-P(2)-Pt(1)	116.1(2)
C(7)-C(9)	1.520(10)	C(13)-P(2)-Pt(1)	114.8(2)
C(7)-C(8)	1.529(11)	C(1)-N(1)-C(11)	121.1(6)
C(7)-C(10)	1.531(10)	C(1)-N(1)-Pt(1)	119.2(5)
C(11)-C(12)	1.355(10)	C(11)-N(1)-Pt(1)	119.5(5)
C(13)-C(15)	1.514(10)	C(21)-N(2)-Pt(1)	123.1(7)
C(13)-C(14)	1.533(11)	C(2)-C(1)-N(1)	122.1(7)
C(13)-C(16)	1.550(10)	C(1)-C(2)-P(1)	115.8(6)
C(17)-C(18)	1.518(10)	C(4)-C(3)-C(5)	108.4(7)
C(17)-C(19)	1.532(10)	C(4)-C(3)-C(6)	109.0(6)
C(17)-C(20)	1.541(9)	C(5)-C(3)-C(6)	109.6(6)
		C(4)-C(3)-P(1)	105.4(5)

C(5)-C(3)-P(1)	113.1(5)	C(15)-C(13)-C(16)	110.6(6)
C(6)-C(3)-P(1)	111.1(5)	C(14)-C(13)-C(16)	107.3(6)
C(9)-C(7)-C(8)	108.0(7)	C(15)-C(13)-P(2)	113.7(5)
C(9)-C(7)-C(10)	110.3(7)	C(14)-C(13)-P(2)	104.9(5)
C(8)-C(7)-C(10)	107.7(6)	C(16)-C(13)-P(2)	110.7(5)
C(9)-C(7)-P(1)	112.2(5)	C(18)-C(17)-C(19)	109.0(7)
C(8)-C(7)-P(1)	105.6(5)	C(18)-C(17)-C(20)	107.4(6)
C(10)-C(7)-P(1)	112.8(5)	C(19)-C(17)-C(20)	109.3(6)
C(12)-C(11)-N(1)	122.1(7)	C(18)-C(17)-P(2)	106.1(5)
C(11)-C(12)-P(2)	115.4(6)	C(19)-C(17)-P(2)	111.7(5)
C(15)-C(13)-C(14)	109.3(6)	C(20)-C(17)-P(2)	113.1(5)

Symmetry transformations used to generate equivalent atoms:

Table S16: Torsion angles [°] for **4**.

C(11)-N(1)-C(1)-C(2)	-175.2(8)	Pt(1)-P(1)-C(7)-C(10)	152.7(5)
Pt(1)-N(1)-C(1)-C(2)	-1.4(10)	C(1)-N(1)-C(11)-C(12)	176.1(8)
N(1)-C(1)-C(2)-P(1)	0.1(10)	Pt(1)-N(1)-C(11)-C(12)	2.3(10)
C(3)-P(1)-C(2)-C(1)	-117.9(6)	N(1)-C(11)-C(12)-P(2)	-1.3(10)
C(7)-P(1)-C(2)-C(1)	122.4(6)	C(17)-P(2)-C(12)-C(11)	-120.8(6)
Pt(1)-P(1)-C(2)-C(1)	0.9(7)	C(13)-P(2)-C(12)-C(11)	119.6(6)
C(2)-P(1)-C(3)-C(4)	55.3(6)	Pt(1)-P(2)-C(12)-C(11)	-0.1(6)
C(7)-P(1)-C(3)-C(4)	170.8(5)	C(12)-P(2)-C(13)-C(15)	63.1(6)
Pt(1)-P(1)-C(3)-C(4)	-53.5(6)	C(17)-P(2)-C(13)-C(15)	-51.5(7)
C(2)-P(1)-C(3)-C(5)	-63.0(6)	Pt(1)-P(2)-C(13)-C(15)	172.7(5)
C(7)-P(1)-C(3)-C(5)	52.5(6)	C(12)-P(2)-C(13)-C(14)	-56.3(6)
Pt(1)-P(1)-C(3)-C(5)	-171.8(5)	C(17)-P(2)-C(13)-C(14)	-170.9(5)
C(2)-P(1)-C(3)-C(6)	173.2(5)	Pt(1)-P(2)-C(13)-C(14)	53.3(5)
C(7)-P(1)-C(3)-C(6)	-71.3(6)	C(12)-P(2)-C(13)-C(16)	-171.6(5)
Pt(1)-P(1)-C(3)-C(6)	64.4(6)	C(17)-P(2)-C(13)-C(16)	73.8(6)
C(2)-P(1)-C(7)-C(9)	167.7(5)	Pt(1)-P(2)-C(13)-C(16)	-62.1(6)
C(3)-P(1)-C(7)-C(9)	52.5(6)	C(12)-P(2)-C(17)-C(18)	75.4(6)
Pt(1)-P(1)-C(7)-C(9)	-82.1(6)	C(13)-P(2)-C(17)-C(18)	-169.3(5)
C(2)-P(1)-C(7)-C(8)	-74.9(6)	Pt(1)-P(2)-C(17)-C(18)	-34.1(6)
C(3)-P(1)-C(7)-C(8)	170.0(5)	C(12)-P(2)-C(17)-C(19)	-166.0(5)
Pt(1)-P(1)-C(7)-C(8)	35.3(6)	C(13)-P(2)-C(17)-C(19)	-50.7(6)
C(2)-P(1)-C(7)-C(10)	42.5(6)	Pt(1)-P(2)-C(17)-C(19)	84.5(5)
C(3)-P(1)-C(7)-C(10)	-72.7(6)	C(12)-P(2)-C(17)-C(20)	-42.2(6)

C(13)-P(2)-C(17)-C(20)	73.1(6)	Pt(1)-P(2)-C(17)-C(20)	-151.7(5)

Symmetry transformations used to generate equivalent atoms:

5 Computational Details

Optimization of molecular geometries and Hessian calculations were carried out with the Gaussian 16 program[17] assuming gas phase conditions. The PBE0 hybrid density functional[18,19] and the def2-SVP basis set[20] with the quasi-relativistic 60-electron pseudopotential (ECP60MWB) for platinum[21] were employed together with the D3 empirical dispersion correction with Becke-Johnson damping,[22–26] abbreviated PBE0-D/def2-SVP. Zero-point energies and thermal contributions to Gibbs free energies at 243 K were obtained at this level of DFT within the ideal gas, rigid-rotor, and harmonic oscillator approximations. Single-point calculations with the def2-TZVPP basis set[20] including the corresponding ECP for the metal center were employed to achieve improved energies, PBE0-D/def2-TZVPP for short. TD-DFT simulations of UV-VIS spectra were performed at this level of DFT together with the SMD implicit solvation model[27] for THF. EPR parameters were calculated using the PBE0 hybrid functional as implemented in the AMS 2022.1[28] program and the triple-zeta Slater-type basis set TZ2P-J[29] in combination with the ZORA Hamiltonian[30–33] and a perturbative or self-consistent inclusion of spin-orbit coupling effects.

Additional Figures

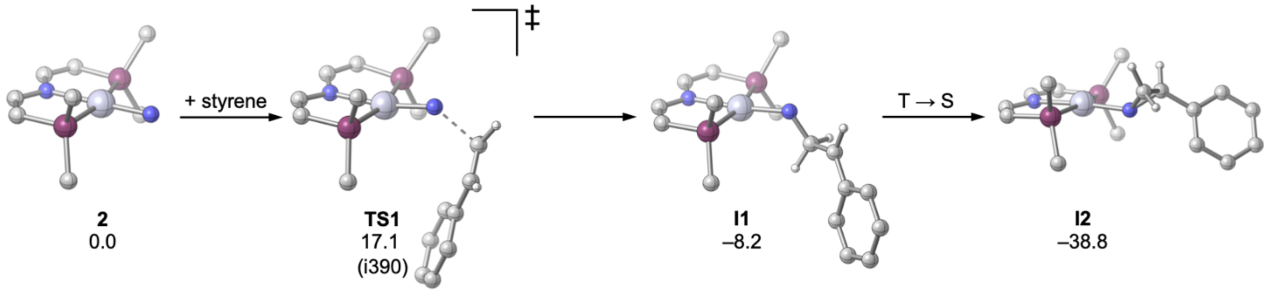

Figure S86: Molecular structures of the intermediates and transition states involved in the reaction of **2** with styrene. H-atoms and CH_3-moieties of ^{t}Bu-groups omitted for clarity; ΔG^{243} in kcal mol^{-1}; transition state imaginary frequencies in cm^{-1}.

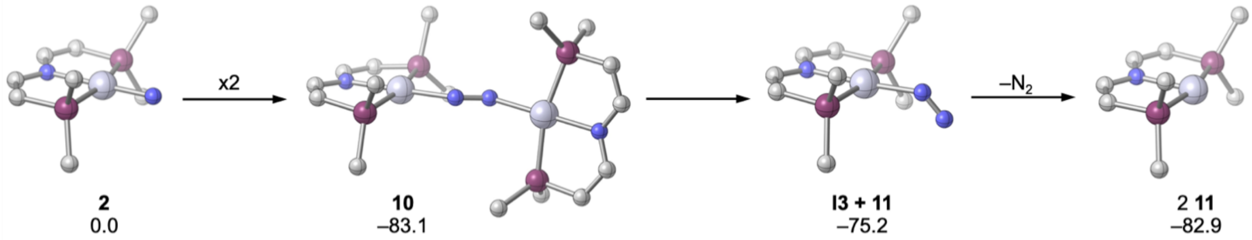

Figure S87: Molecular structures of the intermediates involved in the dimerization of **2** and decay of the dimer. H-atoms and CH_3-moieties of ^{t}Bu-groups omitted for clarity; ΔG^{243} in kcal mol^{-1}.

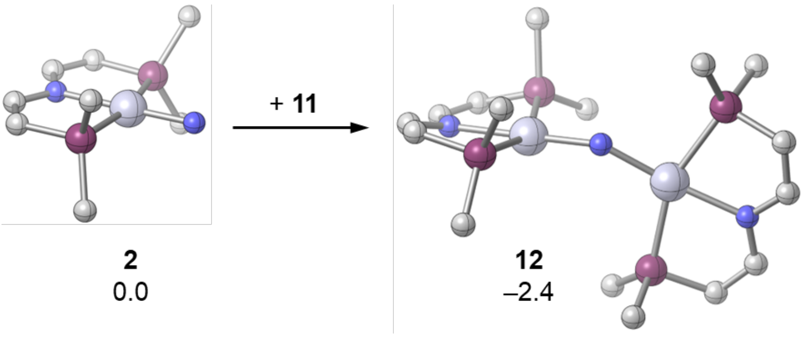

Figure S88: Molecular structures of the **2** and **12,** the product of the radical recombination reaction of **2** and **11**. H-atoms and CH_3-moieties of ^{t}Bu-groups omitted for clarity; ΔG^{243} in kcal mol^{-1}.

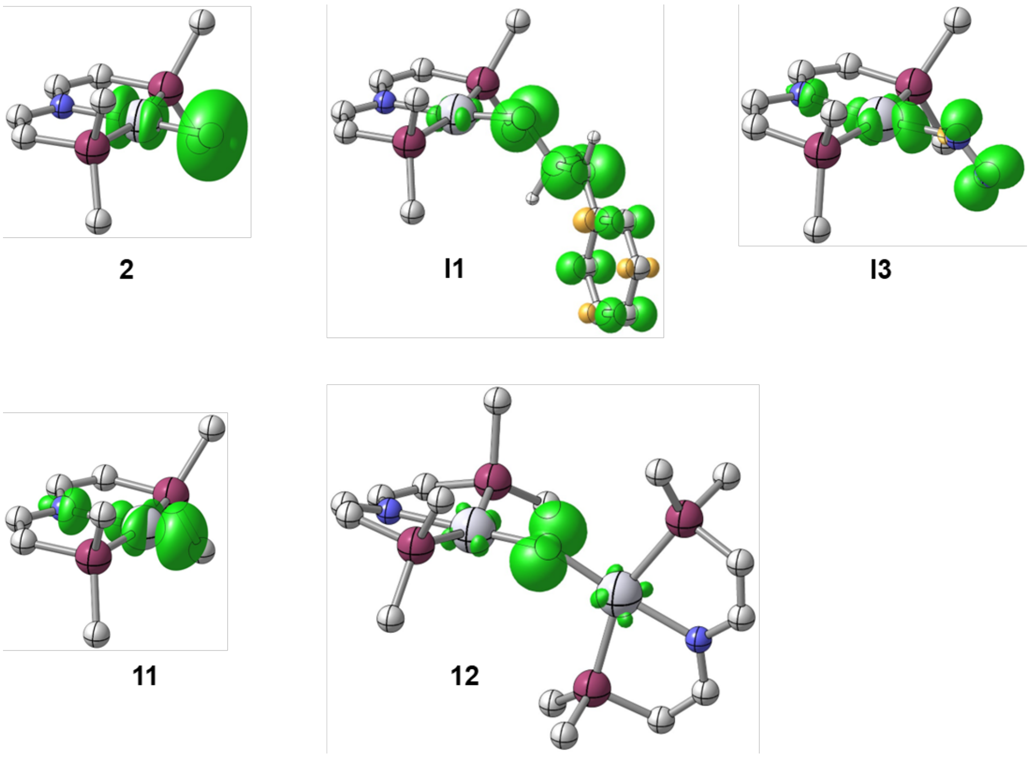

Figure S89: Spin-density plots for **2**, **I1**, **I3**, **11** and **12**. H-atoms and CH_3-moieties of ^{t}Bu-groups omitted for clarity; isosurfaces at 0.01 a_0^{-3}.

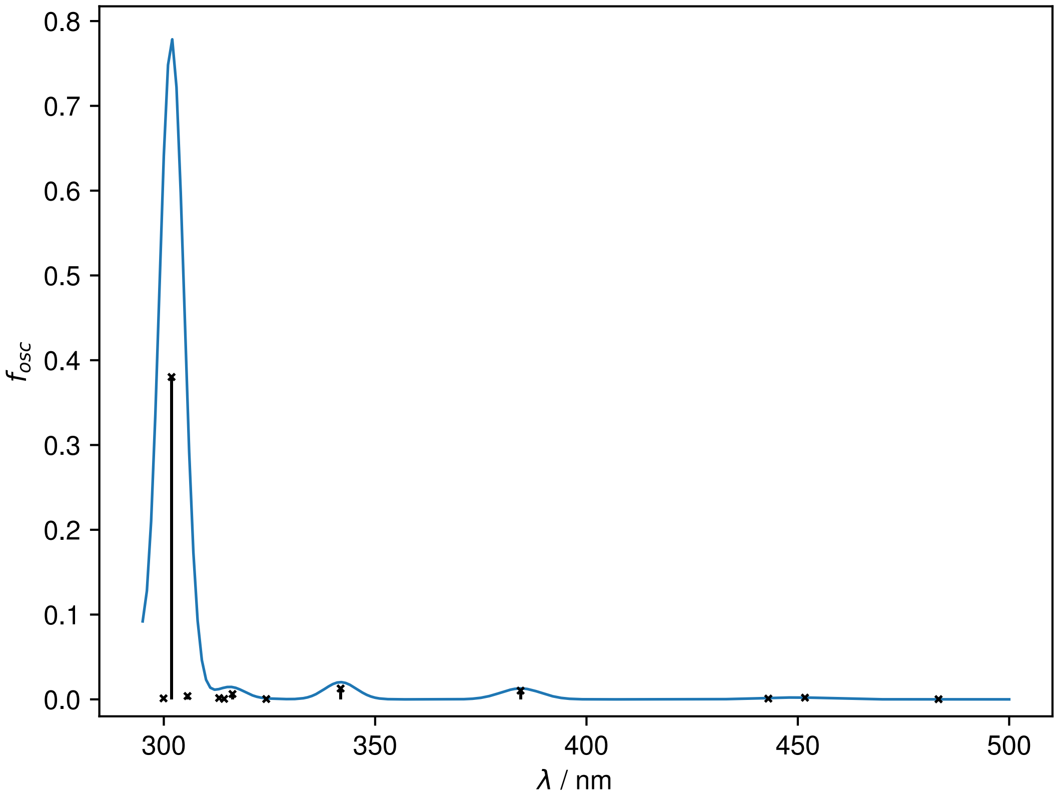

Figure S90: TD-DFT computed UV/Vis spectrum of **2** (black bars and blue envelope curve with σ = 0.04 eV for gaussian line-shape).

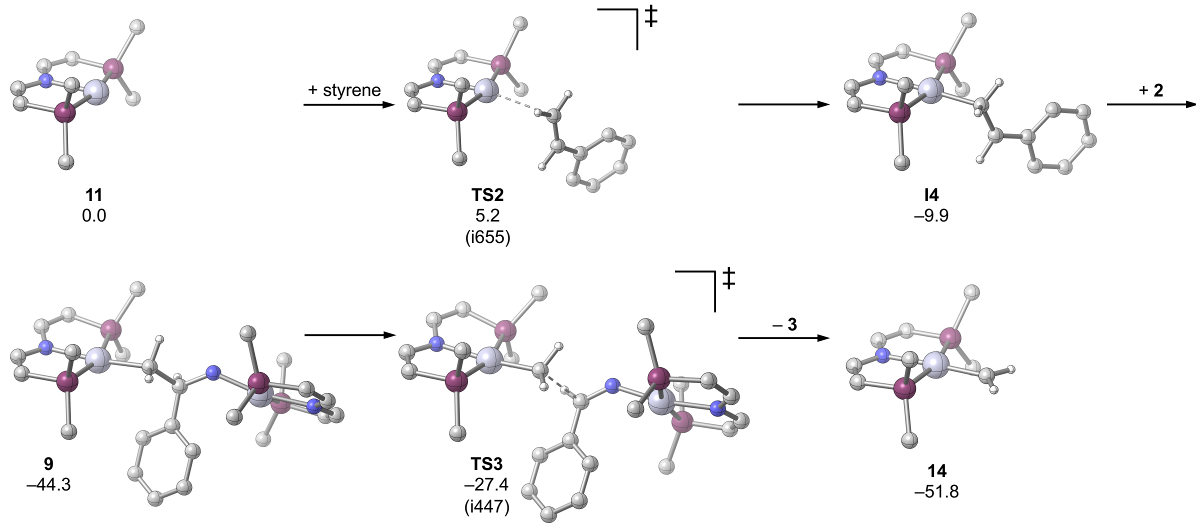

Figure S91: Molecular structures of intermediates and transition states involved in the reaction of **11** with styrene. Intermediate **I4** was referred to as [(PNP)Pt($CH_2C^{\bullet}HPh$)] in the main text. H-atoms and CH_3-moieties of ^{t}Bu-groups omitted for clarity; ΔG^{243} in kcal mol^{-1}; transition state imaginary frequencies in cm^{-1}.

Table S17: Comparison of scalar relativistic ZORA and spin-orbit ZORA computed (PBE0/TZ2P-J) EPR parameters for **9**; *A*-tensors in MHz, Euler angles (*α*, *β*, *γ*) in °.

	Scalar relativistic ZORA			Spin-orbit ZORA		
g	2.126	2.023	2.010	2.106	2.000	1.985
A(Pt_1)	–466	–765	–93	–473	–724	–494
angles	33.5	21.6	82.3	–12.0	9.3	28.0
A(Pt_2)	43	26	34	37	21	24
angles	68.3	55.8	–32.9	–29.8	16.8	60.8
A(P_1)	60	58	78	61	60	80
angles	45.8	24.7	0.0	25.9	29.5	22.0
A(P_2)	75	74	97	76	76	99
angles	–3.3	41.6	50.0	–4.7	29.7	24.3
A(N)	7	1	114	12	6	109
angles	–61.6	21.1	–83.0	46.8	6.2	–56.3
A(C_1)	39	39	50	38	39	50
angles	11.2	143.6	44.3	2.1	49.6	44.5
A(C_2)	–25	–33	–31	–25	–33	–31
angles	–65.5	161.8	–59.8	2.1	49.6	44.5
A(H)	143	125	129	142	125	128
angles	68.5	65.8	–46.3	52.4	55.6	–47.4

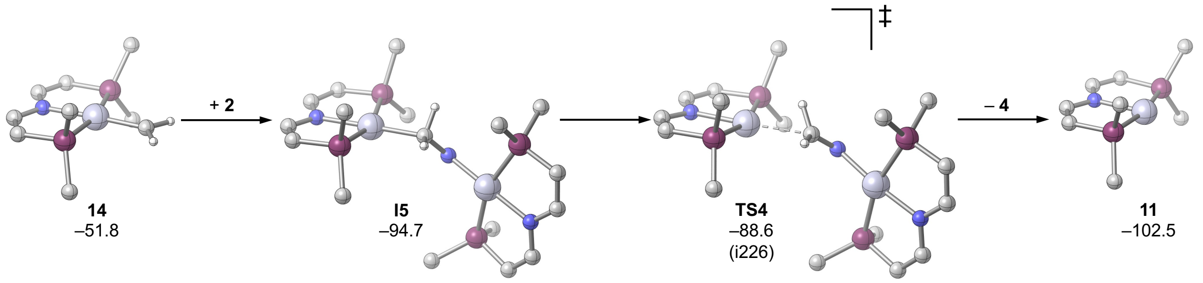

Figure S92: Molecular structures of intermediates and transition states involved in the reaction of **14** with **2**. Intermediate **I5** was referred to as [(CH_2N){Pt(PNP)}$_2$] in the main text. H-atoms and CH_3-moieties of ^{t}Bu-groups omitted for clarity; ΔG^{243} in kcal mol^{-1}; transition state imaginary frequencies in cm^{-1}.

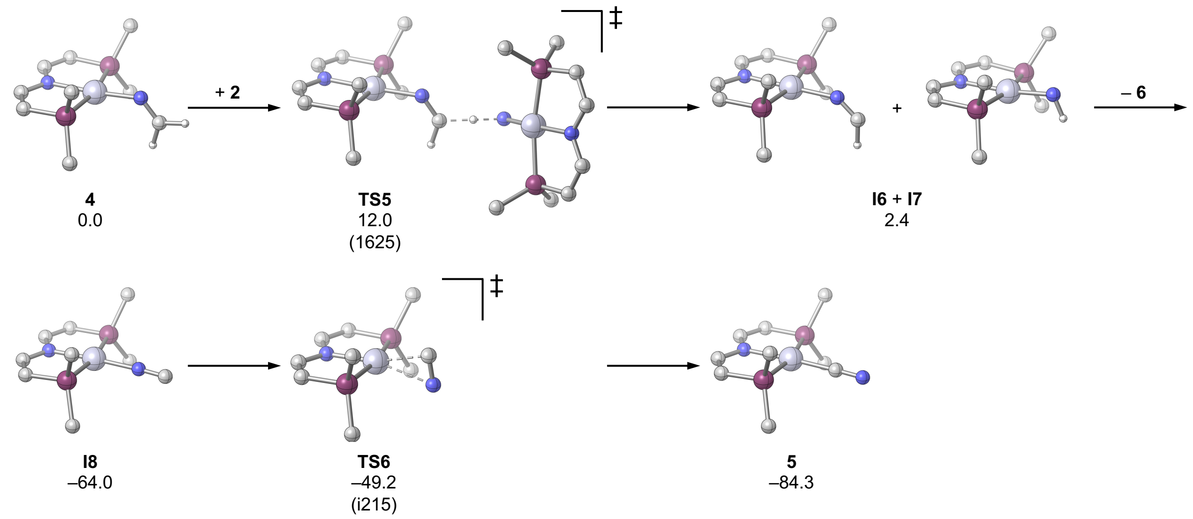

Figure S93: Molecular structures of intermediates and transition states involved in the double HAT from **4** with **2**. H-atoms and CH_3-moieties of ^{t}Bu-groups omitted for clarity; ΔG^{243} in kcal mol^{-1}; transition state imaginary frequencies in cm^{-1}.

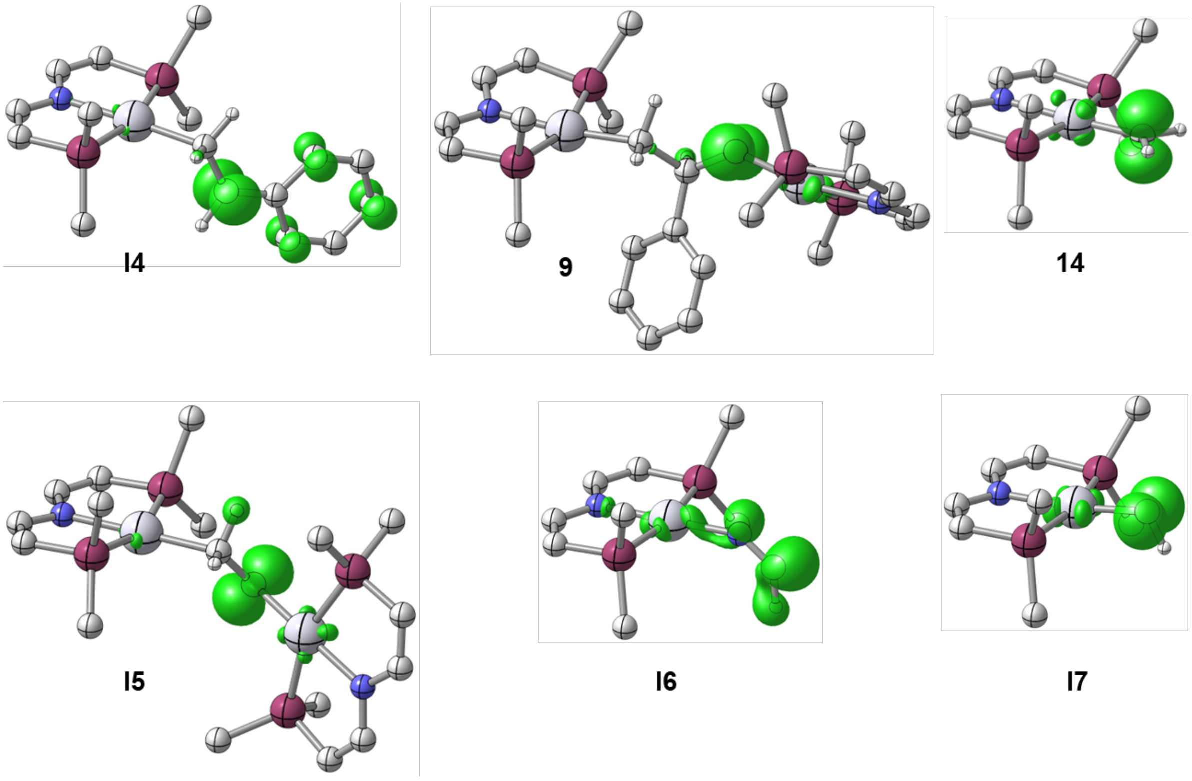

Figure S94: Spin-density plots for **I4**, **9**, **14**, **I5** and **I6** and **I7**. Intermediate **I4** was referred to as [(PNP)Pt($CH_2C^{\bullet}HPh$)] and intermediate **I5** as [(CH_2N){Pt(PNP)}$_2$] in the main text. H-atoms and CH_3-moieties of ^{t}Bu-groups omitted for clarity; isosurfaces at 0.01 a_0^{-3}.

Cartesian Coordinates

Cartesian coordinates of all computed structures are provided as an ASCII text file.

References

(1) Sun, J.; Abbenseth, J.; Verplancke, H.; Diefenbach, M.; Bruin, B. de; Hunger, D.; Würtele, C.; van Slageren, J.; Holthausen, M. C.; Schneider, S. A platinum(II) metallonitrene with a triplet ground state. *Nat. Chem.* **2020**, *12* (11), 1054–1059.

(2) Lv, Z.-J.; Engel, P. D.; Alig, L.; Maji, S.; Holthausen, M. C.; Schneider, S. Stabilizing Doubly Deprotonated Diazomethane: Isolable Complexes with CN22- and CN2- Radical Ligands. *J. Am. Chem. Soc.* **2022**, *144* (48), 21872–21877.

(3) Harris, R. K.; Becker, E. D.; Menezes, S. M. C. de; Granger, P.; Hoffman, R. E.; Zilm, K. W. Further conventions for NMR shielding and chemical shifts (IUPAC Recommendations 2008). *Magn. Reson. Chem.* **2008**, *46* (6), 582–598.

(4) Horino, Y.; Kimura, M.; Tanaka, S.; Okajima, T.; Tamaru, Y. Preparation, structure, and unique thermal 2+2, 4+2, and 3+2 cycloaddition reactions of 4-vinylideneoxazolidin-2-one. *Chem. Eur. J.* **2003**, *9* (11), 2419–2438.

(5) Xu, Y.-H.; Lu, J.; Loh, T.-P. Direct cross-coupling reaction of simple alkenes with acrylates catalyzed by palladium catalyst. *J. Am. Chem. Soc.* **2009**, *131* (4), 1372–1373.

(6) Zhu, Y.; Ehnbom, A.; Fiedler, T.; Shu, Y.; Bhuvanesh, N.; Hall, M. B.; Gladysz, J. A. Platinum(II) alkyl complexes of chelating dibridgehead diphosphines P((CH2)n)3P (n = 14, 18, 22); facile cis/trans isomerizations interconverting gyroscope and parachute like adducts. *Dalton Trans.* **2021**, *50* (36), 12457–12477.

(7) Cavallo, L.; Macchioni, A.; Zuccaccia, C.; Zuccaccia, D.; Orabona, I.; Ruffo, F. Neutral Square-Planar Olefin/Alkyl Platinum(II) Complexes Containing a N , N ‘-Imino−Amide Ligand. Experimental and Theoretical Evidence of Relevant π-Back-Donation in the Platinum−Olefin Bond. *Organometallics* **2004**, *23* (9), 2137–2145.

(8) Bennett, B. L.; Birnbaum, J.; Roddick, D. M. Protonation studies of electrophilic platinum(II) alkyl complexes: synthesis and characterization of (dfepe)Pt(Me)X (X = O2CCF3, OSO2CF3, OSO3H). *Polyhedron* **1995**, *14* (1), 187–195.

(9) Willett, K. L.; Hites, R. A. Chemical Actinometry: Using o-Nitrobenzaldehyde to Measure Lamp Intensity in Photochemical Experiments. *J. Chem. Educ.* **2000**, *77* (7), 900.

(10) Hansch, C.; Leo, A.; Taft, R. W. A survey of Hammett substituent constants and resonance and field parameters. *Chem. Rev.* **1991**, *91* (2), 165–195.

(11) Sur, S. K. Measurement of magnetic susceptibility and magnetic moment of paramagnetic molecules in solution by high-field fourier transform NMR spectroscopy. *J. Magn. Reson.* **1989**, *82* (1), 169–173.

(12) Stoll, S.; Schweiger, A. EasySpin, a comprehensive software package for spectral simulation and analysis in EPR. *J. Magn. Reson.* **2006**, *178* (1), 42–55.

(13) *APEX3 v2016.9-0 (SAINT/SADABS/SHELXT/SHELXL)*; Bruker AXS Inc., 2016.

(14) Sheldrick, G. M. A short history of SHELX. *Acta Crystallogr., Sect. A: Found. Crystallogr* **2008**, *64* (Pt 1), 112–122.

(15) Sheldrick, G. M. Crystal structure refinement with SHELXL. *Acta Crystallogr., Sect. C: Struct. Chem.* **2015**, *C71*, 3–8.

(16) Sheldrick, G. M. SHELXT - integrated space-group and crystal-structure determination. *Acta Crystallogr., Sect. A: Found. Adv.* **2015**, *A71*, 3–8.
(17) Frisch, M. J.; Trucks, G. W.; Schlegel, H. B.; Scuseria, G. E.; Robb, M. A.; Cheeseman, J. R.; Scalmani, G.; Barone, V.; Petersson, G. A.; Nakatsuji, H.; Li, X.; Caricato, M.; Marenich, A. V.; Bloino, J.; Janesko, B. G.; Gomperts, R.; Mennucci, B.; Hratchian, H. P.; Ortiz, J. V.; Izmaylov, A. F.; Sonnenberg, J. L.; Williams-Young, D.; Ding, F.; Lipparini, F.; Egidi, F.; Goings, J.; Peng, B.; Petrone, A.; Henderson, T.; Ranasinghe, D.; Zakrzewski, V. G.; Gao, J.; Rega, N.; Zheng, G.; Liang, W.; Hada, M.; Ehara, M.; Toyota, K.; Fukuda, R.; Hasegawa, J.; Ishida, M.; Nakajima, T.; Honda, Y.; Kitao, O.; Nakai, H.; Vreven, T.; Throssell, K.; Montgomery, Jr., J. A.; Peralta, J. E.; Ogliaro, F.; Bearpark, M. J.; Heyd, J. J.; Brothers, E. N.; Kudin, K. N.; Staroverov, V. N.; Keith, T. A.; Kobayashi, R.; Normand, J.; Raghavachari, K.; Rendell, A. P.; Burant, J. C.; Iyengar, S. S.; Tomasi, J.; Cossi, M.; Millam, J. M.; Klene, M.; Adamo, C.; Cammi, R.; Ochterski, J. W.; Martin, R. L.; Morokuma, K.; Farkas, O.; Foresman, J. B.; Fox, D. J. *Gaussian 16 Revision B.01*, 2016.
(18) Perdew, J. P.; Ernzerhof, M.; Burke, K. Rationale for mixing exact exchange with density functional approximations. *J. Chem. Phys.* **1996**, *105*, 9982–9985.
(19) Adamo, C.; Barone, V. Toward reliable density functional methods without adjustable parameters: The PBE0 model. *J. Chem. Phys.* **1999**, *110*, 6158–6170.
(20) Weigend, F.; Ahlrichs, R. Balanced basis sets of split valence, triple zeta valence and quadruple zeta valence quality for H to Rn: Design and assessment of accuracy. *Phys. Chem. Chem. Phys.* **2005**, *7* (18), 3297–3305.
(21) Andrae, D.; Häußermann, U.; Dolg, M.; Stoll, H.; Preuß, H. Energy-adjusted ab initio pseudopotentials for the second and third row transition elements. *Theor. Chim. Acta* **1990**, *77*, 123–141.
(22) Grimme, S.; Antony, J.; Ehrlich, S.; Krieg, H. A consistent and accurate ab initio parametrization of density functional dispersion correction (DFT-D) for the 94 elements H-Pu. *J. Chem. Phys.* **2010**, *132*, 154104.
(23) Johnson, E. R.; Becke, A. D. A post-Hartree–Fock model of intermolecular interactions. *J. Chem. Phys.* **2005**, *123*, 24101.
(24) Becke, A. D.; Johnson, E. R. A density-functional model of the dispersion interaction. *J. Chem. Phys.* **2005**, *123*, 154101.
(25) Johnson, E. R.; Becke, A. D. A post-Hartree-Fock model of intermolecular interactions: Inclusion of higher-order corrections. *J. Chem. Phys.* **2006**, *124*, 174104.
(26) Grimme, S.; Ehrlich, S.; Goerigk, L. Effect of the damping function in dispersion corrected density functional theory. *J. Comput. Chem.* **2011**, *32*, 1456–1465.
(27) Marenich, A. V.; Cramer, C. J.; Truhlar, D. G. Universal Solvation Model Based on Solute Electron Density and on a Continuum Model of the Solvent Defined by the Bulk Dielectric Constant and Atomic Surface Tensions. *J. Phys. Chem. B* **2009**, *113*, 6378–6396.
(28) te Velde, G.; Bickelhaupt, F. M.; Baerends, E. J.; Fonseca Guerra, C.; van Gisbergen, S. J. A.; Snijders, J. G.; Ziegler, T. Chemistry with ADF. *J. Comput. Chem.* **2001**, *22*, 931–967.
(29) van Lenthe, E.; Baerends, E. J. Optimized Slater-type basis sets for the elements 1–118. *J. Comput. Chem.* **2003**, *24*, 1142–1156.
(30) van Lenthe, E.; Baerends, E. J.; Snijders, J. G. Relativistic regular two-component Hamiltonians. *J. Chem. Phys.* **1993**, *99*, 4597–4610.
(31) van Lenthe, E.; Baerends, E. J.; Snijders, J. G. Relativistic total energy using regular approximations. *J. Chem. Phys.* **1994**, *101*, 9783–9792.
(32) van Lenthe, E.; Snijders, J. G.; Baerends, E. J. The zero-order regular approximation for relativistic effects: The effect of spin–orbit coupling in closed shell molecules. *J. Chem. Phys.* **1996**, *105*, 6505–6516.

(33) Wolff, S. K.; Ziegler, T.; van Lenthe, E.; Baerends, E. J. Density functional calculations of nuclear magnetic shieldings using the zeroth-order regular approximation (ZORA) for relativistic effects: ZORA nuclear magnetic resonance. *J. Chem. Phys.* **1999**, *110*, 7689–7698.
